# Long-term safety and effectiveness of canakinumab therapy in patients with cryopyrin-associated periodic syndrome: results from the β-Confident Registry

**DOI:** 10.1136/rmdopen-2021-001663

**Published:** 2021-05-17

**Authors:** Ulrich A Walker, Hugh H Tilson, Philip N Hawkins, Tom van der Poll, Stephanie Noviello, Jeremy Levy, Eleni Vritzali, Hal M Hoffman, Jasmin B Kuemmerle-Deschner, the CACZ885D2401 Study Investigators

**Affiliations:** 1 Department of Rheumatology, University Hospital Basel, Basel, Switzerland; 2 Gillings School of Global Public Health, University of North Carolina, Chapel Hill, North Carolina, USA; 3 University College London, London, UK; 4 Amsterdam Medical Center, University of Amsterdam, Amsterdam, The Netherlands; 5 Novartis Pharmaceuticals Corporation, East Hanover, New Jersey, USA; 6 Novartis Pharma AG, Basel, Switzerland; 7 University of California San Diego, San Diego, California, USA; 8 Rady Children's Hospital San Diego, San Diego, California, USA; 9 Division of Pediatric Rheumatology, Department of Paediatrics and Autoinflammation Reference Center Tuebingen, University Hospital Tuebingen, Tübingen, Germany

**Keywords:** cryopyrin-associated periodic syndromes, biological therapy, inflammation

## Abstract

**Objective:**

To report the long-term safety and effectiveness of canakinumab, a fully human anti-interleukin 1β monoclonal antibody, in patients with cryopyrin-associated periodic syndromes (CAPS), including familial cold autoinflammatory syndrome (FCAS), Muckle-Wells syndrome (MWS) and neonatal-onset multisystem inflammatory disease (NOMID), in a real-world setting.

**Methods:**

From December 2009 to December 2015, the β-Confident Registry prospectively enrolled patients with CAPS and non-CAPS conditions who received canakinumab per routine care and were prospectively followed for up to 6 years. The registry protocol did not mandate specific visits or procedures; however, all observed adverse events (AEs) and serious adverse events (SAEs) had to be recorded. Canakinumab effectiveness was evaluated by Physician’s Global Assessment (PGA).

**Results:**

Of 288 patients enrolled, 3 were excluded due to missing informed consent. Among the remaining 285 patients, 243 (85.3%) were patients with CAPS and 42 (14.7%) had atypical CAPS (6.3%) or other conditions (8.4%). The median age was 26.6 years. Based on PGA, 58 of 123 (47.2%) patients with CAPS had no disease activity at 48 months, and 65 of 123 (52.8%) experienced mild/moderate disease activity at 48 months. Among CAPS phenotypes, AE incidence rates per 100 patient-years were lowest for FCAS (73.1; 95% CI 60.3 to 87.8) compared with those with MWS (105.0; 95% CI 97.2 to 113.2) or NOMID (104.6; 95% CI 86.6 to 125.2). One hundred twenty-eight SAEs were reported in 68 patients with CAPS (incidence rate/100 patient-years, 14.0; 95% CI 11.6 to 16.6). One death (metastatic rectal adenocarcinoma in a patient with MWS) was reported.

**Conclusions:**

The response to canakinumab was sustained for up to 6 years. Canakinumab demonstrated a favourable safety profile over long-term treatment in patients with CAPS.

**Trial registration number:**

NCT01213641.

**Key messages**

**What is already known about this subject?**

- Cryopyrin-associated periodic syndrome (CAPS) is an autoinflammatory disease encompassing a spectrum of three phenotypes with an estimated prevalence ranging from one to three per million.
- Canakinumab, a fully human anti-interleukin 1β monoclonal antibody, has shown rapid control of disease activity and prevention of disease-related morbidities in patients with CAPS.

**What does this study add?**

- The multinational β-Confident Registry is the largest prospective CAPS cohort in paediatric and adult patients with prior exposure to various biologics and other immunomodulatory treatments.
- The long-term data from the registry demonstrate the favourable safety profile of canakinumab, supporting the long-term use of canakinumab in patients with CAPS, and also show sustained effectiveness of canakinumab in patients with CAPS for up to 6 years.

**How might this impact on clinical practice or further developments?**

- The results of the β-Confident Registry are reassuring for physicians and patients and support the long-term use of canakinumab in clinical practice.

## Introduction

Cryopyrin-associated periodic syndromes (CAPS) represent a continuum of three inherited, autoinflammatory phenotypes ranging by disease severity from mild to severe as familial cold autoinflammatory syndrome (FCAS), Muckle-Wells syndrome (MWS), and neonatal-onset multisystem inflammatory disease /chronic infantile neurological cutaneous and articular syndrome (NOMID/CINCA). These phenotypes share symptoms including recurrent fever, headache, influenza-like musculoskeletal pain, conjunctivitis and fatigue.^1 2^ Patients with FCAS present with recurrent, cold-induced episodes of fever, urticaria-like rash and arthralgia. MWS is complicated by sensorineural hearing loss and systemic amyloidosis, and NOMID additionally presents with early-onset central nervous system inflammation and bone deformities.^1–3^ The prevalence of CAPS is not well characterised, ranging from 1 to 2 per million in the USA to 1 per 360 000 in France.^3^

CAPS are associated with upregulated interleukin (IL) 1β production due to mutations in the nucleotide-binding oligomerisation domain (NOD)-like receptor family, pyrin domain containing 3 (*NLRP3*) gene.^4^ *NLRP3* encodes cryopyrin, a key inflammasome component that triggers caspase-1 enzyme activation, which in turn catalyses the cleavage of pro-IL-1β into active IL-1β, a potent proinflammatory and pyrogenic cytokine. Several studies have shown that blocking IL-1 activity is an effective mechanism for the treatment of CAPS.^5–7^

In pivotal studies canakinumab, a selective, human anti-IL-1β monoclonal antibody, demonstrated rapid control of disease activity and prevention of disease-related morbidities.^8 9^ However, long-term data of canakinumab in patients with CAPS are unavailable. The β-Confident Registry was initiated to provide long-term safety data as well as information regarding disease progression and cognitive and maturational development in children using canakinumab in routine clinical practice for CAPS.

## Methods

### Study design

The β-Confident Registry (Clinical outcomes and safety: a registry study of canakinumab patients) was an open-label, multicentre, long-term, prospective, observational study to monitor the safety and effectiveness of canakinumab administered per local prescribing information^10^ in paediatric (aged ≥2 or ≥4 (depending on local label) to ≤17 years) and adult patients with CAPS for a minimum of 5 years following marketing authorisation. The registry was conducted from December 2009 to December 2015 and prospectively enrolled patients with CAPS and, to a lesser extent, other medical conditions (including atypical CAPS and other autoinflammatory/autoimmune diseases). Patients were followed for a minimum of 1 year. All patients receiving canakinumab as part of their medical care were eligible to participate. There were no exclusion criteria. Written informed consent (and paediatric assent, as applicable) was obtained for each patient.

### Data collection

Although registry participation did not mandate regular visits, study sites were recommended to collect data at baseline and 6-month follow-up intervals until the end of study. An electronic data capture system was used to collect data, but no specific set of clinical, laboratory or procedural data was mandated.^11^ Baseline parameters included demographic and disease characteristics such as CAPS phenotype (FCAS, MWS, NOMID) and genotype (presence of *NLRP3* mutation), disease duration, prior assessments of disease activity, sexual and neurocognitive development, medication history for autoinflammatory disease and vaccination statusonline supplemental file 2.

10.1136/rmdopen-2021-001663.supp2 Supplementary data

Autoinflammatory disease activity was collected at baseline and during the study using Physician’s Global Assessment (PGA) on a Likert scale (absent, mild/moderate, severe). Similarly, organ-specific symptoms (ie, skin disease, arthralgia, myalgia/limb pain, headache/migraine, conjunctivitis, fatigue/malaise, fever/chills, cold-induced symptoms, abdominal pain, oral ulcers) were assessed on a Likert scale. Markers of inflammation including C reactive protein (CRP) and serum amyloid A (SAA) were also reported. Available audiograms, ophthalmological and neurological examinations, brain MRI results and cerebrospinal fluid analysis were collected. In patients between 6 and 18 years, growth and sexual maturation was assessed by Tanner stages, while neurocognitive function was assessed by the local paediatrician.

Safety was assessed by reported adverse events (AEs) and serious adverse events (SAEs) from baseline until the last assessment, with particular focus on serious infections, malignancies, hypersensitivity reactions and vertigo. AEs were coded using Medical Dictionary for Regulatory Activities version 14.1.^12^

### Statistical analysis

The EULAR recommendation for reporting safety data of biologics registers in rheumatology was followed.^13^ As this study was descriptive in nature, no formal hypothesis testing or statistical testing was conducted.

Analyses are based on the set of patients who signed informed consent. The data presented herein focus on the CAPS population (FCAS, MWS, NOMID). Continuous data are presented by median values and corresponding quartiles (Q1–Q3). Categorical data are provided as numbers (n) and proportions (%). Canakinumab dose at baseline is displayed by age categories and distinct phenotypes (FCAS, MWS, NOMID) and ‘Other’. PGA is analysed by phenotypes (including total CAPS) and by subgroups defined by prior exposure to IL-1 treatments including anakinra, rilonacept or canakinumab, as part of a clinical trial. Frequencies of AEs are presented as exposure-adjusted incidence rate per patient-years (IR/PY) and 95% CI.

### Patient and public involvement

Patients and the public were not involved in the design/conduct of the study.

## Results

### Demographics and disease characteristics

Thirty-eight sites in 13 countries in Europe and the USA participated from December 2009 until December 2015. The registry enrolled 288 patients, of whom 3 were excluded due to missing informed consent. Among the remaining 285 patients, 243 (85.3%) were patients with CAPS, including 42 (17.3%) with FCAS, 169 (69.5%) with MWS and 32 (13.2%) with NOMID (table 1). An additional 42 (14.7%) patients were in the ‘Other’ category with atypical CAPS (18, 6.3%) or other autoinflammatory/autoimmune diseases (24, 8.4%).

**Table 1:** Demographic and disease characteristics at baseline

	FCAS (n=42)	MWS (n=169)	NOMID (n=32)	All CAPS (n=243)	Other* (n=42)	All (N=285)
Age <4 years, n (%)	1 (2.4)	6 (3.6)	0	7 (2.9)	2 (4.8)	9 (3.2)
Age 4–<12 years, n (%)	7 (16.7)	25 (14.8)	11 (34.4)	43 (17.7)	10 (23.8)	53 (18.6)
Age 12–<18 years, n (%)	3 (7.1)	25 (14.8)	7 (21.9)	35 (14.4)	10 (23.8)	45 (15.8)
Age of paediatric patients, median (Q1–Q3), years	9.1 (5.8–14.3)	11.5 (6.2–14.2)	8.2 (6.2–15.0)	11.1 (6.2–14.3)	10.4 (7.3–13.4)	10.8 (6.2–14.3)
Age ≥18 years, n (%)	31 (73.8)	113 (66.9)	14 (43.8)	158 (65.0)	20 (47.6)	178 (62.5)
Age of adult patients, median (Q1–Q3), years	43.9 (31.9–60.7)	42.9 (33.1–50.8)	24.1 (20.2–41.0)	42.6 (28.9–51.6)	51.7 (25.3–57.2)	42.8 (28.6–53.4)
Female, n (%)	28 (66.7)	82 (48.5)	17 (53.1)	127 (52.3)	26 (61.9)	153 (53.7)
Caucasian, n (%)	27 (64.3)	132 (78.1)	25 (78.1)	184 (75.7)	24 (57.1)	208 (73.0)
*NLRP3* mutation positive, n (%)	39 (92.9)	158 (93.5)	30 (93.8)	227 (93.4)	10 (23.8)	237 (83.2)
Estimated duration of disease, median (Q1–Q3), years	28.5 (8–50)	17.5 (8–40)	14.0 (6–20)	18.0 (8–40)	14.5 (9.5–33)	18.0 (8–40)
History						
Sensorineural hearing loss, n (%)	3 (7.1)	96 (56.8)	20 (62.5)	119 (49.0)	7 (16.7)	126 (44.2)
Vertigo, n (%)	0.0	6 (3.6)	3 (9.4)	9 (3.7)	3 (7.1)	12 (4.2)
Audiogram, abnormal, n (%)	18 (42.9)	52 (30.8)	7 (21.9)	77 (31.7)	2 (4.8)	79 (27.7)
Uveitis, n (%)	3 (7.1)	16 (9.5)	11 (34.4)	30 (12.3)	4 (9.5)	34 (11.9)
Papillitis/papilloedema, n (%)	0.0	14 (8.3)	18 (56.3)	32 (13.2)	1 (2.4)	33 (11.6)
Meningitis, n (%)	2 (4.8)	10 (5.9)	15 (46.9)	27 (11.1)	1 (2.4)	28 (9.8)
Daily headaches, n (%)	3 (7.1)	27 (16.0)	19 (59.4)	49 (20.2)	5 (11.9)	54 (18.9)
Laboratory						
CRP, median mg/L (Q1–Q3)	1.5 (1.0–8.0)	4.2 (1.0–9.0)	5.4 (2.0–13.0)	4.0 (1.0–9.4)	6.0 (1.0–13.0)	4.9 (1.0–10.0)
CRP >5 mg/L, n (%)	11 (26.2)	48 (28.4)	15 (46.9)	74 (30.5)	19 (45.2)	93 (32.6)
SAA, median mg/L (Q1–Q3)	3.0 (2.0–37.0)	7.0 (3.0–22.0)	12.0 (5.0–77.0)	6.0 (3.0–34.0)	5.0 (3.5–6.5)	6.0 (3.0–23.0)
SAA >10 mg/L, n (%)	6 (14.3)	50 (29.6)	12 (37.5)	68 (28.0)	3 (7.1)	71 (24.9)
Creatinine elevation†, n (%)	3 (7.1)	14 (8.3)	4 (12.5)	21 (8.6)	2 (4.8)	23 (8.1)
Proteinuria by dipstick†, n (%)	2 (4.8)	10 (5.9)	2 (6.3)	14 (5.8)	2 (4.8)	16 (5.6)
Abnormal CSF†, n (%)	0.0	7 (4.1)	11 (34.4)	18 (7.4)	1 (2.4)	19 (6.7)

*Other includes atypical CAPS (patients with a confirmed *NLRP3* mutation and an atypical clinical presentation or patients with typical CAPS symptoms but without confirmed pathogenic mutations in *NLRP3*; n=18) and non-CAPS diagnoses (n=24); non-CAPS diagnoses include systemic juvenile idiopathic arthritis (n=8), unspecified autoinflammatory syndromes (n=5), familial Mediterranean fever (n=3), mevalonate kinase deficiency (n=2), adult-onset Still’s disease (n=2), tumour necrosis factor receptor-associated periodic syndrome (n=1), Erdheim-Chester disease (n=1), Blau syndrome (n=1) and granulomatosis with polyangiitis (n=1).

†Creatinine levels, proteinuria and CSF abnormalities assessed by the investigator as clinically significant.

CAPS, cryopyrin-associated periodic syndrome; CRP, C reactive protein; CSF, cerebrospinal fluid; FCAS, familial cold autoinflammatory syndrome; MWS, Muckle-Wells syndrome; *NLRP3*, nucleotide-binding oligomerisation domain (NOD)-like receptor family, pyrin domain containing 3 gene; NOMID, neonatal-onset multisystem inflammatory disease; SAA, serum amyloid A.

Of the 243 patients with CAPS, 85 (35.0%) were <18 years of age, including 7 (2.9%) patients <4 years old. The remainder were adults with a median age of 42.6 years. Nearly all (93.4%) patients with CAPS had an *NLRP3* mutation. The median symptomatic disease duration was 18.0 years, with variation across the different CAPS indications (median (Q1–Q3): FCAS, 28.5 (8.0–50.0); MWS, 17.5 (8.0–40.0); NOMID, 14.0 (6.0–20.0)). Approximately half of the patients with CAPS had baseline sensorineural hearing loss.

Prior to enrolment, 64 of 243 (26.3%) patients with CAPS had received corticosteroids. Among CAPS phenotypes, a higher proportion of patients with NOMID had received corticosteroids (15 of 32, 46.9%) and conventional synthetic disease-modifying antirheumatic drugs (8 of 32, 25.0%). Two-thirds of patients with CAPS had received either canakinumab in a clinical trial or an anti-IL-1 agent other than canakinumab (online supplemental table S1). Of 243 patients with CAPS, 85 (35.0%) had received canakinumab only as marketed product without participating in a clinical trial. Due to the potential for inappropriate dosing or short-term use of canakinumab, analyses of these patients should be interpreted with caution.

10.1136/rmdopen-2021-001663.supp1 Supplementary data

Based on PGA at baseline, 8 of 218 (3.7%) patients with CAPS had severe disease activity and 104 of 218 (47.7%) had no disease activity. Approximately 80 of 145 (55.2%) patients with CAPS with previous exposure to anti-IL-1 treatment had no disease activity compared with 24 of 73 (32.9%) without previous anti-IL-1 exposure.

### Canakinumab exposure and patient disposition

The majority of patients with CAPS were administered 2–<4 mg/kg of canakinumab (online supplemental table S2). The median (Q1–Q3) exposure duration to marketed canakinumab prior to the start of the registry was 37.7 (4.3–58.1) weeks in patients with CAPS and 34.9 weeks (4.0–58.1) in all registry patients. The median (Q1–Q3) duration of exposure to canakinumab during the course of this registry was 4.4 (2.8–4.7) years in the CAPS population, with a slightly lower duration in children aged 6–<12 years and in patients aged ≥65 years (3.4 (1.2–4.4) and 3.1 (1.3–4.6) years, respectively). The study duration was 4.4 years (Q1–Q3: 2.8–4.8) for the CAPS population. Forty-eight (19.8%) patients with CAPS discontinued the registry before completion. The reasons cited were lost to follow-up (n=20, 41.7%), site requested study close-out (n=8, 16.7%), change of physician (n=4, 8.3%), AEs (n=3, 6.3%), withdrawal of informed consent (n=2, 4.2%), changed therapy (n=2, 4.2%), pregnancy (n=1, 2.1%), death (n=1, 2.1%), lack of efficacy (n=1, 2.1%) and other (n=6, 12.5%). An additional 12 ‘Other’ patients discontinued the study.

### Long-term impact of canakinumab on disease activity

**Figure 1:**
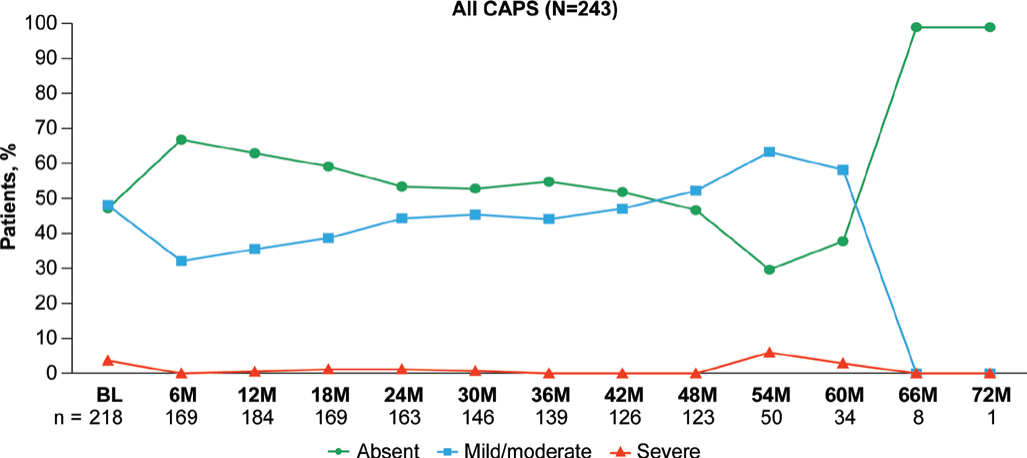
Physician’s Global Assessment of autoinflammatory disease activity in patients with CAPS over time. Note that the number of patients decreased after 48 months. Percentages are based on the number of available patients at the respective time point. BL, baseline; CAPS, cryopyrin-associated periodic syndrome; M, months; N, number of patients in the group; n, number of evaluable patients.

PGA, CRP and SAA were focused on the first 48 months of this registry due to the decrease in the number of evaluable patients after 48 months. Among patients with CAPS, disease activity after 48 months on canakinumab treatment was largely unchanged from baseline (figure 1). The proportion having no disease activity was 47.7% (104 of 218) at baseline and 47.2% (58 of 123) at 48 months, while the proportion of patients with severe disease activity decreased from 3.7% (8 of 218) at baseline to 0% at 48 months. PGA disease activity over the time of treatment with canakinumab by CAPS phenotypes is shown in online supplemental figure S1A. The proportion of patients having no disease activity increased from baseline to 48 months in patients with CAPS without previous exposure to anti-IL-1 treatment (32.9% (24 of 73) to 41.4% (12 of 29)) and slightly decreased in those with previous exposure to IL-1 inhibitors (55.2% (80 of 145) to 48.9% (46 of 94); online supplemental figure S1 A, B).

The median (Q1–Q3) levels of CRP and SAA in patients with CAPS decreased slightly from 4.0 (1.0–9.4) mg/L and 6.0 (3.0–34.0) mg/L, respectively at baseline, to 3.0 (1.0–5.0) mg/L and 5.0 (3.0–9.1) mg/L, respectively, at month 48 (online supplemental figure S1C).

The results of audiograms, ophthalmological examinations and brain MRIs are summarised in online supplemental table S3. At the last available assessment, audiograms were abnormal in 80 of 147 (54.4%) patients with CAPS. Of these, only five had normal assessments at baseline. Conversely, of the 86 patients with abnormal results at baseline, 11 had normal results at the last assessment. Similarly, ophthalmological examinations at the last available assessment showed abnormal results in 26 of 111 (23.4%) patients with CAPS, of whom 8 patients had a normal assessment at baseline. Of the 32 patients who had abnormal ophthalmological examinations at baseline, 9 had normal results at their last available assessment. Brain MRI was abnormal in 9 of 55 (16.4%) patients with CAPS at the last available assessment; of these, 4 patients had normal assessments at baseline.

### Neurocognitive development

Neurocognitive function was to be assessed for 66 patients with CAPS between 6 and <18 years of age (online supplemental table S4). At baseline, neurocognitive dysfunction was reported in 6 of 59 (10.2%) patients with CAPS with available assessments. A delay in neurocognitive function was noted in 6 of 58 (10.3%) patients with CAPS at the last available assessment; 5 of the 6 patients had delayed neurocognitive development at baseline.

### Sexual maturation

Sexual maturation was to be assessed for patients with CAPS between 6 and <18 years old (online supplemental table S4). A delay in sexual development was reported at baseline in 2 of 48 (4.2%) patients with CAPS with available assessments, but in none of the 49 patients with available postbaseline evaluations.

### Adverse events

Of the 285 patients, 223 (78.2%) reported 1114 AEs (IR/100 PY, 105.3; 95% CI 99.2 to 111.6) (online supplemental table S5). Among the 243 patients with CAPS, 187 (77.0%) reported 914 AEs (IR/100 PY, 99.5; 95% CI 93.2 to 106.2). On comparing paediatric with adult patients with CAPS, the IRs of AEs were similar (IR/100 PY, 101.1 and 98.8, respectively). Among the CAPS phenotypes, AEs were lowest for patients with FCAS (IR/100 PY, 73.1; 95% CI 60.3 to 87.8) compared with MWS (IR/100 PY, 105.0; 95% CI 97.2 to 113.2) and NOMID (IR/100 PY, 104.6; 95% CI 86.6 to 125.2). Infections and infestations were the most frequently reported AEs in patients with CAPS (IR/100 PY, 37.3; 95% CI 33.5 to 41.5), with slightly decreasing IRs from patients with NOMID (IR/100 PY, 39.6) to patients with MWS (IR/100 PY, 37.6), to patients with FCAS (IR/100 PY, 34.6). The most common infections and infestations included nasopharyngitis, gastroenteritis and influenza. Ten non-serious AEs reported in seven patients with CAPS were opportunistic infections, including five events of herpes zoster, three events of varicella and two events of herpes virus infection.

Patients experiencing any AE were numerically lower in the CAPS population dosed with <2 mg/kg of canakinumab than patients treated with ≥2 mg/kg of canakinumab (72.1% (44 of 61) vs 80.1% (125 of 156); online supplemental table S6). Among the AEs judged as related to canakinumab in patients with CAPS, infections and infestations were most commonly reported (IR/100 PY, 13.7; 95% CI 11.4 to 16.3; online supplemental table S7). The most common among these were nasopharyngitis, lower respiratory tract infections, urinary tract infections, tonsillitis and influenza.

#### Serious adverse events

In 68 patients with CAPS, 128 SAEs were reported (IR/100 PY, 14.0; 95% CI 11.6 to 16.6) (table 2 and online supplemental table S8). In 15 ‘Other’ patients, 27 SAEs were reported. The IR/100 PY was lowest for patients with FCAS (2.6; 95% CI 0.7 to 6.6) compared with patients with MWS (15.4; 95% CI 12.6 to 18.8) and NOMID (21.1; 95% CI 13.5 to 31.4). Among patients with CAPS, the IRs of SAEs were slightly lower in paediatric patients (IR/100 PY, 12.6) compared with adult patients (IR/100 PY, 14.6). The most commonly reported SAEs in patients with CAPS were pneumonia, pancreatitis, headache, tonsillitis and urinary tract infection. All six SAEs of pancreatitis occurred in one patient with MWS on multiple concomitant medications and with cholelithiasis; the events resolved while continuing on canakinumab and were not considered related to study drug. SAEs occurred with similar frequency in patients with CAPS regardless of canakinumab dose administered (online supplemental table S6).

**Table 2:** Serious adverse events listed in descending order of incidence by patient group*

Preferred term* n (IR/100 PY) (95% CI)	FCAS (n=42)	MWS (n=169)	NOMID (n=32)	All CAPS (n=243)	Other† (n=42)	All (N=285)
Any SAE	4 (2.6) (0.7 to 6.6)	100 (15.4) (12.6 to 18.8)	24 (21.1) (13.5 to 31.4)	128 (13.9) (11.6 to 16.6)	27 (19.3) (12.73 to 28.11)	155 (14.7) (12.4 to 17.2)
Pneumonia	0 (0.0 to 2.4)	5 (0.8) (0.3 to 1.8)	0 (0.0 to 3.2)	5 (0.54) (0.2 to 1.3)	2 (1.43) (0.17 to 5.17)	7 (0.7) (0.3 to 1.4)
Pancreatitis	0 (0.0 to 2.4)	6 (0.9) (0.3 to 2.0)	0 (0.0 to 3.2)	6 (0.7) (0.2 to 1.4)	0 (0.0 to 2.7)	6 (0.6) (0.2 to 1.2)
Headache	0 (0.0 to 2.4)	1 (0.2) (0.0 to 0.9)	3 (2.6) (0.5 to 7.7)	4 (0.4) (0.1 to 1.1)	0 (0.0 to 2.7)	4 (0.4) (0.1 to 1.0)
Tonsillitis	1 (0.6) (0.0 to 3.6)	2 (0.3) (0.0 to 1.1)	0 (0.0 to 3.2)	3 (0.3) (0.1 to 1.0)	1 (0.7) (0.0 to 4.0)	4 (0.4) (0.1 to 1.0)
Urinary tract infection	0 (0.0 to 2.4)	3 (0.5) (0.1 to 1.4)	1 (0.9) (0.0 to 4.9)	4 (0.4) (0.1 to 1.1)	0 (0.0 to 2.7)	4 (0.4) (0.1 to 1.0)
Angina pectoris	0 (0.0 to 2.4)	3 (0.5) (0.1 to 1.4)	0 (0.0 to 3.2)	3 (0.3) (0.1 to 1.0)	0 (0.0 to 2.7)	3 (0.3) (0.1 to 0.8)
Bronchitis	0 (0.0 to 2.4)	3 (0.5) (0.1 to 1.4)	0 (0.0 to 3.2)	3 (0.3) (0.1 to 1.0)	0 (0.0 to 2.7)	3 (0.3) (0.1 to 0.8)
Depression	0 (0.0 to 2.4)	2 (0.3) (0.0 to 1.1)	1 (0.9) (0.0 to 4.9)	3 (0.3) (0.1 to 1.0)	0 (0.0 to 2.7)	3 (0.3) (0.1 to 0.8)
Intracranial pressure increased	0 (0.0 to 2.4)	0 (0.0 to 0.6)	2 (1.8) (0.2 to 6.4)	2 (0.2) (0.0 to 0.8)	1 (0.7) (0.0 to 4.0)	3 (0.3) (0.1 to 0.8)
Rectal cancer	0 (0.0 to 2.4)	3 (0.5) (0.1 to 1.4)	0 (0.0 to 3.2)	3 (0.3) (0.1 to 1.0)	0 (0.0 to 2.7)	3 (0.3) (0.1 to 0.8)
Vaccination site inflammation	0 (0.0 to 2.4)	3 (0.5) (0.1 to 1.4)	0 (0.0 to 3.2)	3 (0.3) (0.1 to 1.0)	0 (0.0 to 2.7)	3 (0.3) (0.1 to 0.8)
Vertigo	0 (0.0 to 2.4)	1 (0.2) (0.0 to 0.9)	2 (1.8) (0.2 to 6.4)	3 (0.3) (0.1 to 1.0)	0 (0.0 to 2.7)	3 (0.3) (0.1 to 0.8)
Abortion spontaneous	0 (0.0 to 2.4)	2 (0.3) (0.0 to 1.1)	0 (0.0 to 3.2)	2 (0.2) (0.0 to 0.8)	0 (0.0 to 2.7)	2 (0.2) (0.0 to 0.7)
Abscess	0 (0.0 to 2.4)	2 (0.3) (0.0 to 1.1)	0 (0.0 to 3.2)	2 (0.2) (0.0 to 0.8)	0 (0.0 to 2.7)	2 (0.2) (0.0 to 0.7)
Cellulitis	0 (0.0 to 2.4)	1 (0.2) (0.0 to 0.9)	1 (0.9) (0.0 to 4.9)	2 (0.2) (0.0 to 0.8)	0 (0.0 to 2.7)	2 (0.2) (0.0 to 0.7)
Cholelithiasis	0 (0.0 to 2.4)	2 (0.3) (0.0 to 1.1)	0 (0.0 to 3.2)	2 (0.2) (0.0 to 0.8)	0 (0.0 to 2.7)	2 (0.2) (0.0 to 0.7)
Condition aggravated	0 (0.0 to 2.4)	1 (0.2) (0.0 to 0.9)	1 (0.9) (0.0 to 4.9)	2 (0.2) (0.0 to 0.8)	0 (0.0 to 2.7)	2 (0.2) (0.0 to 0.7)
Glaucoma	0 (0.0 to 2.4)	1 (0.2) (0.0 to 0.9)	1 (0.9) (0.0 to 4.9)	2 (0.2) (0.0 to 0.8)	0 (0.0 to 2.7)	2 (0.2) (0.0 to 0.7)
Knee deformity	0 (0.0 to 2.4)	0 (0.0 to 0.6)	2 (1.8) (0.2 to 6.4)	2 (0.2) (0.0 to 0.8)	0 (0.0 to 2.7)	2 (0.2) (0.0 to 0.7)
Lower respiratory tract infection	0 (0.0 to 2.4)	1 (0.2) (0.0 to 0.9)	1 (0.9) (0.0 to 4.9)	2 (0.2) (0.0 to 0.8)	0 (0.0 to 2.7)	2 (0.2) (0.0 to 0.7)
Meningitis	0 (0.0 to 2.4)	0 (0.0 to 0.6)	2 (1.8) (0.2 to 6.4)	2 (0.2) (0.0 to 0.8)	0 (0.0 to 2.7)	2 (0.2) (0.0 to 0.7)
Perirectal abscess	0 (0.0 to 2.4)	2 (0.3) (0.0 to 1.1)	0 (0.0 to 3.2)	2 (0.2) (0.0 to 0.8)	0 (0.0 to 2.7)	2 (0.2) (0.0 to 0.7)
Pyrexia	0 (0.0 to 2.4)	1 (0.2) (0.0 to 0.9)	1 (0.9) (0.0 to 4.9)	2 (0.2) (0.0 to 0.8)	0 (0.0 to 2.7)	2 (0.2) (0.0 to 0.7)

*Number of events ≥2 in all registry patients; see online supplemental table S8 for number of events=1 in all registry patients.

†Other includes atypical CAPS (n=18) and non-CAPS diagnoses (n=24); non-CAPS diagnoses include systemic juvenile idiopathic arthritis (n=8), unspecified autoinflammatory syndromes (n=5), familial Mediterranean fever (n=3), mevalonate kinase deficiency (n=2), adult-onset Still’s disease (n=2), tumour necrosis factor receptor-associated periodic syndrome (n=1), Erdheim-Chester disease (n=1), Blau syndrome (n=1) and granulomatosis with polyangiitis (n=1).

CAPS, cryopyrin-associated periodic syndromes; FCAS, familial cold autoinflammatory syndrome; IR, incidence rate; MWS, Muckle-Wells syndrome; n, number of events; NOMID, neonatal-onset multisystem inflammatory disease; PY, patient-years; SAE, serious adverse event.

#### Serious infections

Forty-three serious infections were reported in 32 (13.2%) patients with CAPS (IR/100 PY, 4.7; 95% CI 3.4 to 6.3). Patients with NOMID had a slightly higher IR/100 PY (6.2; 95% CI 2.5 to 12.7) compared with patients with MWS (5.4; 95% CI 3.8 to 7.5), and only one patient with FCAS (0.6; 95% CI 0.0 to 3.6) reported a serious infection. The most common serious infections were pneumonia, urinary tract infection, bronchitis and tonsillitis (table 2).

#### Malignancies

Fourteen events of malignant and benign neoplasms were reported among 11 (4.5%) patients with CAPS (online supplemental table S5). Three events of rectal cancer were reported in one 76-year-old patient with MWS who had a fatal outcome; the events were not considered related to study drug.

#### Hypersensitivity reactions

There were four events (IR/100 PY, 0.4) of drug hypersensitivity among patients with CAPS (online supplemental table S5).

#### Vertigo

Vertigo episodes were more frequently reported in patients with NOMID and MWS (IR/100 PY, 7.9 and 3.2, respectively) compared with patients with FCAS (IR/100 PY, 0.6) (online supplemental table S5).

## Discussion

To our knowledge, this is the largest CAPS registry, remarkably the largest CAPS paediatric cohort study (85 patients aged <18 years) to date, describing the effectiveness and long-term safety of canakinumab in patients with CAPS from 38 centres across 13 countries in Europe and the USA. While patients were followed for up to 6 years in this registry, the total exposure was even longer for over 40% of patients with CAPS already exposed to canakinumab in previous CAPS clinical trials. In this registry, patients with CAPS with long-standing disease, chronic/irreversible organ damage and past and present use of corticosteroids and other cytotoxic and biologic drugs were enrolled, indicating a difficult-to-treat population.

Autoinflammatory disease activity, as measured by PGA, improved or remained stable over time for most patients. Disease activity was absent or mild/moderate in more than 90% of the patients throughout the study. As a substantial proportion of patients (>40%) were already receiving canakinumab prior to enrolment in this registry, these results show the sustained long-term effectiveness of canakinumab in the study population for >48 months. These results are consistent with those reported by Lachmann *et al*,^8^ in which 97% of patients were rated as having no or minimal disease activity at the end of study and the remaining patients had mild disease activity. Patients with previous exposure to anti-IL-1 treatment rarely had severe disease at baseline and over time compared with patients without previous exposure to anti-IL-1 treatment, most probably reflecting the beneficial effect of anti-IL-1 treatment maintained over time. The median level of CRP over 48 months of follow-up was normal and similar to that reported by Lachmann *et al* 8 for 15 patients who received canakinumab throughout the 48-week study, 3.0 mg/L and 1.9 mg/L, respectively. The median level of SAA stabilised within normal limits at 5.0 mg/L early, similar to the previous report of 5.1 mg/L. The maintenance of low SAA levels suggests a potential decline in the long-term risk of AA amyloidosis. Together, these findings support the long-term, sustained efficacy of canakinumab.

Clinical findings suggestive of permanent organ damage, such as hearing and/or visual impairment, were more frequently encountered in patients with long-standing disease but presented as early as the first decade in patients with NOMID, which could be attributed to historically poor control of disease activity in conjunction with the severity of the phenotype.^14^ Audiogram, ophthalmological examination and brain MRI results were similar between baseline and the last available assessment. However, four of nine patients with CAPS developed new brain MRI abnormalities and one of six patients with CAPS developed neurocognitive dysfunction while being treated with canakinumab in this registry. The efficacy of canakinumab in preventing neurological damage in this patient population should be further studied. Canakinumab did not appear to impact sexual maturation based on limited data for patients aged 6 to <18 years.

The profile of AEs and SAEs, including infections and other events of interest, reported during the course of this registry was consistent with the known safety profile for canakinumab.^8 15 16^ The proportion of patients with CAPS reporting AEs in this registry (77.0%) was similar to that observed in the previous pivotal trial (77.4%).^8^ However, about twice as many patients with CAPS experienced SAEs in this registry (28.0%) compared with the published study (12.9%), likely due to the longer duration of exposure to canakinumab (3.6 years on average vs 5.6 months).^8^ Serious infections were reported in 13.2% of patients with CAPS. These patients with CAPS had a slightly higher IR of serious infections (IR/100 PY, 4.7) than patients treated in the CANTOS trial (IR/100 PY, 4.0), which had shown that canakinumab is associated with a higher rate of serious infections compared with placebo.^17^ No new or unexpected safety concerns or previously unrecognised SAEs were identified.

Most malignancies were reported in adults and/or elderly patients. One death was reported in a 76-year-old patient with end-stage metastatic rectal cancer, which was not suspected to be related to the study drug. While the small number of vertigo events among patients with CAPS in this registry specifically occurred among patients with MWS, patients with NOMID had the highest IR. The safety of vaccinations in patients with CAPS from the β-Confident Registry has been reported previously.^18^

As with any registry data collection, this study does not capture all of the clinical information for the patients enrolled. Most of the patients enrolled were from the USA and Europe, which may limit generalisability of the results to a broader population worldwide. While there were a number of older children in the study, there were few young children and therefore safety data in this small sample size should be interpreted with caution.

Long-term data from the β-Confident Registry confirm the effectiveness and safety of canakinumab treatment in paediatric and adult patients with CAPS in routine clinical practice. Results demonstrated that the response of patients with CAPS reported in this registry is consistent with previous studies.^8 15 16^ Given the rarity of the condition, data from this registry play a vital role in expanding the current understanding of the disease in the largest CAPS cohort that may help early diagnosis of patients, providing more effective treatment under real-world circumstances, eventually improving the overall quality of life of patients with CAPS.

## Data Availability

Data are available upon reasonable request. All data relevant to the study are included in the article or uploaded as supplementary information. The datasets generated during the current study are not publicly available. Novartis is committed to sharing with qualified external researchers access to patient-level data and supporting clinical documents from eligible studies. These requests are reviewed and approved on the basis of scientific merit. All data provided are anonymised to respect the privacy of patients who have participated in the study in line with applicable laws and regulations. The data may be requested from the corresponding author of the manuscript.
