# Supplementary material for: Long-term safety and effectiveness of canakinumab therapy in patients with cryopyrin-associated periodic syndrome: results from the β-Confident Registry
Source: RMD Open. 2021 May 17;7(2):e001663. doi: 10.1136/rmdopen-2021-001663 (PMC8130749; doi:10.1136/rmdopen-2021-001663)
Supplement: Supplementary data [file rmdopen-2021-001663supp002.pdf]

NOVARTIS

Clinical Development & Medical Affairs

ILARIS® (canakinumab)

Observational Study Protocol CACZ885D2401

β-Confident
Clinical Outcomes and Safety: A Registry Study of Ilaris® (canakinumab) Patients

An open-label, long-term, prospective, observational study to monitor the safety and effectiveness of Ilaris in CAPS patients

Authors: [redacted]

Document type: Amended Protocol Version

EUDRACT number: (not applicable)

Version number: Version 02 clean

Development phase: IV

Release date: 24-Apr-2013

Property of Novartis
Confidential
May not be used, divulged, published, or otherwise disclosed
without the consent of Novartis

[redacted]

Table of contents

Table of contents 2
List of tables 3
List of abbreviations 4
Amendment 2 6
Summary of previous amendments 6
Protocol synopsis 8
1 Background 10
2 Purpose and Rationale 11
3 Objectives 12
3.1 Primary objective 12
3.2 Secondary objectives 12
4 Study design 12
5 Population and setting 13
5.1 Inclusion criteria 13
5.2 Exclusion criteria 14
6 Exposure and assessments 14
6.1 Data collection schedule 14
6.2 Patient demographics/other baseline characteristics 15
6.3 Clinical outcomes 16
6.4 Treatment exposure 17
6.5 Safety 17
6.6 Effectiveness/Disease Progression 17
7 Safety monitoring 18
7.1 Adverse events 18
7.2 Serious adverse event reporting 19
7.3 Pregnancies 19
7.4 Adjudication Committees 19
7.4.1 MAS Adjudication Committee 19
7.4.2 Infection Adjudication Committee 20
7.4.3 Malignancy Adjudication Committee 20
8 Data acquisition 20
8.1 Site monitoring 21
9 Data analysis 21
9.1 Patient demographics/other baseline characteristics 21

[redacted]

Supplemental material BMJ Publishing Group Limited (BMJ) disclaims all liability and responsibility arising from any reliance placed on this supplemental material which has been supplied by the author(s) RMD Open

9.2 Exposure 22
9.3 Analysis of registry objectives 22
9.3.1 Statistical hypothesis, model, and method of analysis 22
9.3.2 Handling of missing values/censoring/discontinuations 22
9.3.3 Supportive analyses 22
9.4 Sample size/power calculation 23
9.5 Registry reporting 23
10 Ethical considerations 23
10.1 Regulatory and ethical compliance 23
10.2 Informed consent procedures 23
10.3 Registry governance 24
10.4 Responsibilities of the treating physician and IRB/IEC/REB 24
10.5 Publication of results 24
11 Protocol amendments 24
12 References 25

List of tables

Table 6-1 Recommended data collection schedule 14

[redacted]

List of abbreviations

AA	Amyloid A
AE	Adverse event
ANA	Antinuclear antibodies
ATC	Anatomical Therapeutic Chemical classification
CAPS	Cryopyrin-Associated Periodic Syndromes
CHMP	Committee for Medicinal Products for Human Use
CI	Confidence interval
CINCA	Chronic Infantile Neurologic Cutaneous Articular
eCRF/CRF	electronic Case Report Form/Case Report Form
CRO	Contract Research Organization
CRP	c-reactive protein
Ds-DNA	Double-stranded deoxyribonucleic acid
DS&E	Drug Safety & Epidemiology
EDC	Electronic Data Capture
eGFR	Estimated glomerular filtration rate
ESR	Erythrocyte sedimentation rate
FCAS	Familial Cold Autoinflammatory Syndrome
IAC	Infection Adjudication Committee
ICH	International Conference on Harmonization
ICMJE	International Committee of Medical Journal Editors
IEC	Independent Ethics Committee
IL-1	Interleukin 1
IRB	Institutional Review Board
MAC	Malignancy Adjudication Committee
MAS	Macrophage Activation Syndrome
MASAC	Macrophage Activation Syndrome Adjudication Committee
MedDRA	Medical Dictionary for Regulatory Affairs
MWS	Muckle-Wells Syndrome
NOMID	Neonatal Onset Multisystem Inflammatory Disease

[redacted]

NSAIDs	Non-steroidal anti-inflammatory drugs
RMP	Risk Management Plan
REB	Research Ethics Board
SAE	Serious adverse event
SAP	Statistical Analysis Plan
SJIA	Systemic Juvenile Idiopathic Arthritis

[redacted]

Supplemental material BMJ Publishing Group Limited (BMJ) disclaims all liability and responsibility arising from any reliance placed on this supplemental material which has been supplied by the author(s) RMD Open

Amendment 2

Amendment rationale

As part of the review of the Ilaris CAPS label extension dossier, the Committee for Medicinal Products for Human Use (CHMP) proposed to modify the protocol of the registry study CACZ885D2401 to possibly increase the proportion of pediatric patients with CAPS and further adjust the anticipated number of patients to be enrolled.

The purpose of this amendment is to address this CHMP proposal: After implementation of this amended protocol, enrolment of adult patients will be stopped and restricted to pediatric CAPS patients aged ≥2 or ≥4 (depending on local label) to ≤17 years of age. The collection of safety data of patients with other autoimmune diseases who were so far allowed to enter this registry study (e.g. gout, systemic juvenile idiopathic arthritis [SJIA]) can be better addressed by enrolling those patients in dedicated registries. Follow-up of all patients in this registry study will continue until one year after the last of a total of 260 expected patients will have been recruited.

Adverse event collecting and reporting have been adapted to meet the requirement of EMA guideline on good pharmacovigilance practices (GVP).

Three independent adjudication committees (Macrophage Activation Syndrome Adjudication Committee (MASAC), Infection Adjudication Committee (IAC) and a Malignancy Adjudication Committee (MAC)) are added to comply with the current Ilaris (canakinumab) Risk Management Plan (RMP). Please see Section 7.4 for additional details.

Information provided in the Section 1 was updated.

Changes to the protocol

Changes of specific sections of the protocol are shown in the track changes version of the protocol using ~~strike through red font for deletions~~ and <u>red underlined for insertions.</u>

Regulatory approval

A copy of this amended protocol will be sent to the Institutional Review Board (IRBs)/Independent Ethics Committee (IECs) and Health Authorities.

The changes described in this amended protocol require IRB/IEC approval prior to implementation. In addition, if the changes herein affect the Informed Consent, sites are required to update and submit for approval a revised Informed Consent that takes into account the changes described in this amended protocol.

Summary of previous amendments

Amendment 1

Amended protocol version 01, released on 22-Jul-2010, was prepared to fulfill a request by the Health Authority in Spain to only include patients using Ilaris as per label in order for the study design to comply with being a prospective observational study. Therefore this

[redacted]

amendment allowed only CAPS patients above the age of 4 to be included in the study in Spain and in other countries with a similar Health Authority request.

The amendment additionally provided clarification to the data collection schedule and timing of data entry. It included the addition of vaccination record and data on growth and development in children to the data collection plan.

Two versions of the original protocol existed – one for Switzerland and one for all other countries (i.e. rest of world [ROW]). The versions did not differ significantly as they included the same study design and population. Both protocol versions were amended and consolidated into one global version (CACZ885D2401 Amended Protocol Version 01) to be implemented in all countries.

[redacted]

Supplemental material BMJ Publishing Group Limited (BMJ) disclaims all liability and responsibility arising from any reliance placed on this supplemental material which has been supplied by the author(s) RMD Open

Protocol synopsis

Title of study: β-CONFIDENT-Clinical Outcomes and Safety: A Registry Study of Ilaris® (canakinumab) Patients

Purpose and rationale: As with all very rare diseases, the clinical development program with canakinumab included a very limited number of patients; therefore, the post-approval period is a critical phase in which to gather more knowledge regarding the short- and long-term safety, effectiveness and treatment patterns associated with use of the product by clinicians and patients.

Availability of anti-interleukin-1β therapies is an important medical breakthrough to fulfill an unmet medical need in the treatment of auto-inflammatory diseases and specifically patients with cryopyrin-associated periodic syndromes (CAPS). Other treatment to date has included NSAIDs and corticosteroids however, only mild improvement in symptoms has been reported. Ilaris® (canakinumab; Novartis) is an anti-interleukin-1β therapy indicated for treatment of CAPS (Familial Cold Autoinflammatory Syndrome [FCAS], Muckle-Wells Syndrome [MWS], and Neonatal Onset Multisystem Inflammatory Disease [NOMID]) in adults and children 2 or 4 years of age (depending on local label) and older with body weight of 7.5 kg or above. Canakinumab specifically blocks IL-1β, the form of the IL-1 that causes disease flares in these auto-inflammatory diseases.

The purpose of this observational study (registry) is to gather additional information with respect to long-term safety and effectiveness of Ilaris® (canakinumab) used to treat patients with CAPS in routine clinical practice.

Objectives:

The primary objective of the β-CONFIDENT Registry is to:

- Monitor the overall safety of Ilaris® in CAPS through the incidence of serious infections, malignancies, hypersensitivity reactions and other selected events.

The secondary objectives of the β-CONFIDENT Registry are to:

- Describe the long-term impact of Ilaris® on disease progression (including systemic AA amyloidosis as evidenced by renal function, neurologic and ophthalmologic symptoms, and sensorineural deafness);
- Explore growth and development patterns of children aged ≥2 or ≥4 (depending on local label) to ≤17 years of age exposed to Ilaris®;
- Identify previously unrecognized serious adverse drug reactions in the treated population;
- Describe the usage and patterns of dosing of Ilaris® in routine clinical practice.

Population: The Registry population will consist of CAPS patients treated with Ilaris®. At study start it was estimated that at least 200 patients treated at approximately 80 sites in 35 countries worldwide would be enrolled. The recruitment of adults is terminated after implementation of this amended protocol version 02 and restricted to children aged ≥2 or ≥4 (depending on local label) to ≤17 years of age with CAPS, to achieve an approximate sample size of 260 patients. The intent of the Registry is to capture data for patients exposed to Ilaris® following marketing authorization; therefore, patient and site numbers are estimates and are expected to change.

Inclusion/Exclusion criteria: CAPS patients aged ≥2 or ≥4 (depending on local label) to ≤17 years of age are eligible for inclusion in this Registry if they receive Ilaris® treatment as part of their medical care.

Adult patients enrolled before the implementation of this amended protocol version 02 are allowed to remain in the Registry until the study is completed.

Study design: The β-CONFIDENT Registry is a multi-center, long-term, prospective, observational study to monitor and further describe the long-term safety and effectiveness of Ilaris® in CAPS. The Registry is planned to run for a minimum of 5 years during which eligible patients can be enrolled.

[redacted]

After completion of enrollment the patients will be followed up for at least one year. There are no required visits associated with the Registry. Data collected for the registry is captured during routine clinical care. Registry data is expected to result from routine medical assessments performed at the initiation and follow-up of Ilaris® treatment, including safety and clinical outcomes. It is expected that in routine clinical care most patients will receive treatment approximately every 8 weeks at their treating physician's office, although it is recognized that variation may occur in the real-world setting. The planned registry data-entry time points are designed to accommodate the pattern of care that most patients will receive in routine clinical practice. All treatment and evaluations for their disease will be determined by the treating physicians according to standard of care and local clinical practice. Baseline data regarding patient characteristics (including genotype and phenotype), disease course, family and medical history, Ilaris® treatment and previous and concomitant treatments will be collected at baseline if available from patient records. Information about treatment outcomes, including effectiveness and safety, and patterns of Ilaris® treatment will be collected as part of follow-up. The information is collected in a standardized and structured way and will be entered if available.

Safety assessments: Any patient who receives at least one dose of Ilaris® will be included in the evaluation for safety. Data regarding the occurrence of the following selected adverse events will be specifically solicited:

- Serious infections
- Malignancies
- Hypersensitivity reactions
- Development of autoantibodies
- Clinical manifestations of an autoimmune disease
- Vertigo
- Any suspected adverse events

All serious adverse events (SAEs) that are reported during follow-up will also be collected.

In addition, ALL non-serious adverse events which have been reported since 02-Jul-2012 will also be collected during follow-up.

Clinical assessments: Additional assessments of effectiveness will include data collection related to global subjective physician assessment of disease response, signs of disease progression [i.e. amyloid A (AA) amyloidosis, renal dysfunction, sensorineural deafness and blindness/vision loss], relevant laboratory assessments (if performed in routine clinical care), vaccination record and outcome, and evaluation of paediatric growth and development (e.g. height, weight, sexual development status, neurocognitive status) if assessed and available in routine clinical care.

Data analysis: No formal sample size calculation was used to manage enrolment in the Registry since the goal is to obtain participation by all eligible patients. No formal hypothesis testing or statistical significance testing is planned. All analyses will be descriptive in nature and fully described in the Statistical Analysis Plan (SAP).

[redacted]

1 Background

Cryopyrin-Associated Periodic Syndromes (CAPS), specifically Familial Cold Autoinflammatory Syndrome (FCAS), Muckle-Wells Syndrome (MWS), and Neonatal Onset Multisystem Inflammatory Disease (NOMID), are a group of rare hereditary autoinflammatory diseases. The prevalence and incidence of these very rare conditions are not well-characterized. These syndromes are typically a result of an autosomal dominant or *de novo* mutation of the cold-induced auto-inflammatory 1 (CIAS1)/nod-like receptor protein 3 (NLRP3) gene on chromosome 1 (Neven 2008). Although it remains poorly understood precisely how CIAS/NLRP-3 mutations cause inflammatory diseases, it is known that the protein encoded by this gene, NALP3 or cryopyrin, interacts with other intracellular proteins to form an intracellular complex called the inflammasome. This complex is important for innate immunity as it detects intracellular pathogens and other danger signals. Mutations in the CIAS/NLRP-3 gene cause up-regulation of NALP3 and activation of the inflammasome, resulting in an overproduction of interleukin 1 (IL-1), a proinflammatory cytokine (Neven 2008, Hawkins 2004).

These conditions were categorized as CAPS once it was recognized that they are generally associated with mutations in the single CIAS1/NLRP-3 gene and are part of a spectrum of disease with overlapping traits and differences in severity, rather than distinct genetic disorders (Lequerre 2007). The conditions may be generally described as life-long, recurrent fever episodes accompanied by differing degrees of neutrophil-mediated systemic inflammation. Intermittent, disruptive exacerbations or flares can be triggered at any time by exposure to cool temperatures, stress, exercise, or other stimuli. Flares can include fever, fatigue, rashes, arthralgia, myalgia headaches, and in more severe cases, lead to severe complications such as hearing loss, blindness, and amyloidosis resulting in kidney failure.

FCAS, on the less severe end of the spectrum, most commonly manifests as fever, cold-induced skin rashes, eye pain and redness, and arthralgia, while MWS classically presents with fever, chills, urticaria, swollen and painful joints, and in many cases progressive deafness and AA reactive systemic amyloidosis. Both FCAS and MWS are typically first noted in infancy, early childhood or adolescence.

NOMID, also known as Chronic Infantile Neurologic Cutaneous Articular (CINCA) syndrome is a severe, sporadic form of the condition presenting in the neonatal period with multi-organ system inflammatory involvement, including significant central nervous system manifestations, such as chronic meningitis, cognitive and mental disabilities, seizures, and sensory organ dysfunction, not seen in other forms of CAPS. These patients may also experience abnormal growth development, hepatomegaly, splenomegaly, and joint and bone deformities, including an enlarged patella.

With all three phenotypes, but especially with MWS, there is substantial variability and unpredictability in symptom type and severity. The symptoms associated with even the milder forms of CAPS can be debilitating. Various symptomatic treatments are used to alleviate the pain and discomfort associated with the inflammatory flares, and patients and their families can become consumed by efforts to avoid potential stimuli (including cold temperatures and stress).

[redacted]

Until recently, treatment has been limited to non-specific symptomatic anti-inflammatory therapy with limited success. With the identification of the genetic basis for the disease and the common pathway of IL-1, new approaches, to treating these conditions have been identified.

IL-1 plays an important role in innate immunity and the normal inflammatory response. The mechanism of action of canakinumab (a fully human monoclonal antibody, Ilaris®, Novartis) is such that the blockade of IL-1 inhibits this inflammatory response therefore increasing susceptibility to infection. Similarly, the use of monoclonal antibodies within humans more generally has demonstrated that hypersensitivity reactions are also a potential risk, although production of specific anti-canakinumab antibodies has not been observed. Among the numerous roles that IL-1 plays, it is up-regulated in many tumor types and has been implicated in tumor progression and cell proliferation, but it is unknown what the effects of long-term immunosuppression via blockade of IL-1β will have on the overall balance of its systemic effects. Ilaris® is indicated for the treatment of CAPS (Familial Cold Autoinflammatory Syndrome [FCAS], Muckle-Wells Syndrome [MWS], and Neonatal Onset Multisystem Inflammatory Disease [NOMID] in adults and children 2 or 4 years of age (depending on local label) and older with body weight of 7.5 kg or above. Ilaris® is approved for the treatment of CAPS in over 60 countries to date. It was first approved in the USA in June 2009 (for the phenotypes FCAS and MWS only), and in the EU in October 2009.

Ilaris® is currently being investigated in several other diseases, for which inflammation and pain is also expected to be caused by over-production of IL-1β (e.g. acute gouty arthritis or systemic juvenile idiopathic arthritis [SJIA]), and in which Ilaris® reduces inflammation.

Ilaris® indicated for the treatment of gouty arthritis flares has been approved in the Philippines and Russia. In the European Union, Ilaris® has further been approved for the symptomatic treatment of adult patients with frequent gouty arthritis attacks (at least 3 attacks in the previous 12 months) in whom NSAIDs and colchicine are contraindicated, are not tolerated, or do not provide an adequate response, and in whom repeated courses of corticosteroids are not appropriate.

Ilaris® indicated for the treatment of SJIA has been approved in Russia.

A summary of CAPS studies performed with Ilaris® so far and their results is provided in the Investigator Brochure.

2 Purpose and Rationale

As with all very rare (orphan) diseases, the clinical development program for canakinumab included a very limited number of patients; therefore, the post-approval period is a critical phase in which to gather more knowledge regarding the short- and long-term safety, effectiveness and treatment patterns associated with use of the product by clinicians and patients.

Availability of anti-interleukin-1β therapies is an important medical breakthrough to fulfill an unmet medical need in the treatment of auto-inflammatory diseases and specifically patients with CAPS. Other treatment to date has included NSAIDs and corticosteroids however, only

[redacted]

mild improvement in symptoms has been reported. Ilaris® (canakinumab; Novartis) is an anti-interleukin-1β therapy indicated for use within patients with MWS, FCAS and NOMID. Unlike other IL-1 agents like Anakinra or Rilonacept, canakinumab specifically blocks only IL-1β, the form of the IL-1 that causes disease flares in these auto-inflammatory diseases.

The β-CONFIDENT Registry is intended to provide additional information regarding the long-term safety of Ilaris® use in paediatric and adult patients, as well as information regarding the long-term effectiveness and use of Ilaris® in routine clinical practice.

For the purpose of ease, this observational study is also referred to in the text as 'Registry'.

3 Objectives

3.1 Primary objective

The primary objective of the β-CONFIDENT Registry is to monitor and further explore the overall safety of Ilaris® in patients with CAPS focusing on serious infections, malignancies, hypersensitivity reactions, vertigo and other selected events.

3.2 Secondary objectives

The secondary objectives of the β-CONFIDENT Registry are to:

- Describe the long-term impact of Ilaris® on disease progression (including systemic AA amyloidosis as evidenced by renal function, neurologic and ophthalmologic symptoms, and sensorineural deafness);
- Explore growth and development patterns in children aged ≥2 or ≥4 (depending on local label) to ≤17 years of age exposed to Ilaris®;
- Identify previously unrecognized serious adverse drug reactions in the treated population;
- Describe the usage and patterns of dosing of Ilaris® in routine clinical practice.

4 Study design

The β-CONFIDENT Registry is a multi-center, long-term, prospective, observational study to monitor and further describe the long-term safety and effectiveness of Ilaris® in CAPS. This Registry will be conducted in compliance with Volume 9a of The Rules Governing Medicinal Products in the European Union (EudraLex Volume 12a, version September 2008).

The Registry is scheduled to proceed for a minimum of five years, during which patients can be enrolled. Patients receiving Ilaris as part of their routine medical care for CAPS are eligible for participation to this Registry. First patient enrolled occurred in Nov-2009, with follow-up estimated to be completed in 2014. Follow-up of all patients in this registry study will continue until one year after the last of a total of 260 expected patients will have been recruited.

At study start it was estimated that a minimum of 200 patients treated at approximately 80 sites in 35 countries worldwide will be included in the registry. The recruitment of adults is terminated after implementation of this amended protocol version 02 and restricted to children aged ≥2 or ≥4 (depending on local label) to ≤17 years of age with CAPS, to achieve an

[redacted]

approximate sample size of 260 patients. The intent of the Registry is to capture data for CAPS patients exposed to Ilaris® as part of their routine clinical care following marketing authorization; therefore, patient and site numbers are estimates and are expected to change.

There are no protocol–mandated visits or procedures associated with the Registry. Data collection is aligned with standard medical practice and is captured during routine clinic visits. Registry data will result from routine medical assessments performed during the initiation and follow-up of Ilaris® treatment, including safety and other clinical outcomes. Any treatment and evaluation decisions regarding the patient's disease will be determined by the treating physician according to standard of care and local clinical practice. Data regarding baseline patient characteristics (including genotype and phenotype) and disease course, non-auto-inflammatory related medical history, Ilaris® treatment and previous and concomitant treatments will be collected at baseline. Treatment outcomes, including effectiveness and safety, and patterns of Ilaris® treatment will be collected as part of follow-up. Follow-up data collection will accommodate the frequency of routine visits. Patients will be followed until study end or early discontinuation, due to withdrawal of consent or loss to follow-up. Patients who discontinue Ilaris® during Registry participation will continue, whenever possible, to be followed for safety-related information until the end of the Registry.

The design of the study is intended to allow assessment of safety and effectiveness in patients with CAPS who are exposed to Ilaris® in the routine clinical practice. There is no internal comparator group; however, descriptive analyses may include relevant subgroups (e.g., by phenotype, duration of exposure).

5 Population and setting

The Registry population will consist of CAPS patients treated with Ilaris®.

5.1 Inclusion criteria

Patients eligible for inclusion in this Registry must fulfill the following criteria:

- CAPS patients of aged ≥2 or ≥4 (depending on local label) to ≤17 years of age receiving Ilaris® treatment at enrolment as part of their routine clinical care;
- Patients or parent(s)/legal representative of the patient willing and able to provide written informed consent.

Adult patients enrolled before the implementation of this amended protocol version 02 are allowed to remain in the study until the study is completed.

If required by local regulations, an independent ethics committee (IEC), institutional review board (IRB) or Research Ethics Board (REB) must review and approve the protocol, informed consent forms and paediatric assent forms before any patients are consented. Each patient and parent/legal representative must participate in the informed consent process and sign and date an informed consent form for this protocol before any data are collected and accessible to the sponsor. As required, paediatric assent must be obtained. Throughout this document, the requirements for informed consent also apply to assent. Documentation of informed consent and assent must be recorded in the source documents for each patient.

[redacted]

Requirements will be adjusted by country and site to comply fully with local regulations and ethics committee requirements, as needed, such as in countries where older adolescents (e.g., > 15 years) are expected to provide written informed consent similar to adults, rather than assent. In some countries, written informed consent from both parents may be required. If a paediatric patient reaches adult age during registry follow-up, he/she will be asked to sign a current IRB/IEC/REB-approved informed consent form at that time.

5.2 Exclusion criteria

- Patients receiving Ilaris treatment for other autoimmune diseases than CAPS

6 Exposure and assessments

Ilaris® may be discontinued at any time at the discretion of the treating physician. Discontinuation of treatment with Ilaris® does not imply that the patient must be removed from the Registry. Whenever possible, patients exposed to Ilaris® should continue to be followed for safety-related outcomes (refer to the Schedule of Assessments).

Patients and/or their respective parents/legal representative (if minors) have the right to withdraw from the Registry at any time, without prejudice, and are not obliged to state their reasons for Registry discontinuation. If a patient or parent/legal representative declares his or her wish to discontinue from the Registry, the treating physician will complete the Disposition CRF. Before considering a patient lost to follow-up, the site staff will make all reasonable attempts to contact the patient or the parent/legal representative to ensure that a Registry outcome of interest is not the underlying reason for the non-response of the patient.

6.1 Data collection schedule

There are no required visits associated with the Registry. The data collection schedule will accommodate that of the patient's routine clinical care. A recommended data collection schedule is provided below in Table 6-1.

It is expected that in routine clinical care most patients will receive treatment approximately every 8 weeks at their physician's office although it is recognized that variation may occur in the real-world setting. During follow-up, the treating physician or other staff will ideally collect data during routine clinical visits. The timing though will depend on the individual patients' healthcare management and subsequent availability of relevant information. It is recommended to enter data into the Registry every 6 months. However, it is important, during follow-up, that data entry be retrospective to the previous data collection time point in order to capture a complete record of a patient's Ilaris® treatment and outcomes.

Table 6-1 Recommended data collection schedule

**Period**	**Baseline**	**Follow-up (until study end or premature discontinuation)***
**Timing**	**Enrollment**	**Every 6 months**
Informed consent	x	

[redacted]

**Period**	**Baseline**	**Follow-up (until study end or premature discontinuation)***
**Timing**	**Enrollment**	**Every 6 months**
CAPS phenotype	x	
CAPS genotype (NLRP-3 mutation),if available	x	
Non-CAPS medical history	x	
Vital signs (height, weight, blood pressure)	x	x
Ilaris® dosing/status	x	x
Historical and concomitant medications for auto-inflammatory disease*	x	x
Selected AEs*		x
Other serious AEs and non-serious adverse events*		x
CAPS clinical assessment *	x	x
CAPS-related clinical testing results, if performed	x	x
Selected local laboratory testing	x	x
Sexual development status (paediatric only)	x	x
Neurocognitive status (paediatric only)	x	x
Cerebrospinal fluid analysis	x	x
Vaccination record and outcome	x	x
Pregnancy status (females of child-bearing potential only)		x
Registry disposition		x

* These assessments may also be conducted during follow-up for patients who discontinue Ilaris® but remain in the Registry

6.2 Patient demographics/other baseline characteristics

It is recommended that the following data elements be collected at baseline:

- Informed consent/paediatric assent
- Demographics, including date of birth, race (where permitted by local regulations)
- Vital signs (current height and weight, blood pressure)
- Indication for treatment with Ilaris®
    - NLRP-3 mutation (if available)
    - CAPS: date of symptom onset and subtype
    - Treatment history
- Clinical assessment of autoinflammatory disease activity (e.g., physician’s global assessment, presence and intensity of fever, rash, myalgia, arthralgia, conjunctivitis, headaches)
- Overall results of recent CAPS-related clinical testing, if performed (audiogram, ophthalmologic exam, brain MRI)

[redacted]

Supplemental material BMJ Publishing Group Limited (BMJ) disclaims all liability and responsibility arising from any reliance placed on this supplemental material which has been supplied by the author(s) RMD Open

- Non-CAPS medical history (by medical record, recent physical exam or diagnostic testing), including:
  1. Auditory (sensorineural hearing loss)
  2. Cardiovascular history and risk factors (i.e., hyperlipidemia, hypertension)
  3. Ophthalmic (nystagmus, uveitis, papillitis/papilledema)
  4. Neurologic (chronic meningitis, headaches, vertigo)
  5. Immune, including severe and/or recurrent infections, immunization history and rheumatoid arthritis or other autoimmune disease
  6. Haematologic (anemia or neutropenia)
  7. Malignancy
  8. Other relevant history
- Ilaris® dosing (date of administration and dose)
- Selected laboratory test results, if performed [serum amyloid A (SAA), , c-reactive protein (CRP), erythrocyte sedimentation rate (ESR); any abnormal and clinically significant selected haematology, creatinine, lipids, liver function tests, urinalysis (proteinuria), anti-nuclear antibodies (ANA) and ds-DNA values]
- Cerebrospinal fluid analysis results (if evaluated)
- Paediatric patients age 6 to ≤17 years only: sexual development status (if evaluated), neurocognitive development status (if evaluated),

6.3 Clinical outcomes

The following data elements will be collected as part of clinical follow-up:

- Date of assessment
- Treatment status: discontinuation, interruption or changes in Ilaris® dosing and reason for discontinuation, interruption or change
- Vital signs [current height (paediatric patients only) and weight, blood pressure]
- Changes in concomitant medications related to auto-inflammatory disease
- Changes in medical status, including disease progression:
  - AA amyloidosis and renal dysfunction
  - Hearing and vision loss
  - Blindness/vision loss
- Clinical assessment of autoinflammatory disease activity (symptom presence/intensity)
- Overall results of CAPS-related clinical testing, if performed (audiogram, ophthalmology, brain MRI)
- Selected laboratory test results, if performed [SAA , CRP, ESR, creatinine, lipids, liver function tests, urinalysis (proteinuria), ANA and ds-DNA values]
- Cerebrospinal fluid analysis (if collected)
- Paediatric patients age 6 to ≤17 years only: sexual development status (if evaluated), neurocognitive development status (if evaluated),

[redacted]

- Adverse events and serious adverse reactions (refer to Section 6.5)
- Vaccination record and outcome: including vaccinations within 3 months of baseline and throughout follow-up
- Pregnancy status (female patients of child-bearing potential only)

6.4 Treatment exposure

Although it is expected that most patients will receive treatment every 8 weeks per local labeling, it is recognized that in the clinical setting that dosing may be delayed or interrupted due to personal reasons, physical condition or co-morbidities. In addition to prescribed treatment schedule, actual treatment dates will be recorded, when available.

6.5 Safety

Any patient who receives at least one dose of Ilaris® will be included in the evaluation for safety. Data regarding the occurrence of the following selected adverse events will be specifically solicited:

- Serious infections

Serious infections are defined as any infections that meet serious criteria (refer to Section 7.1).

- Malignancies
- Hypersensitivity reactions to Ilaris®
- Development of autoantibodies (ANA, ds-DNA)
- Clinical manifestations of an autoimmune disease (e.g., lupus, vasculitis, rheumatoid arthritis)
- Vertigo
- Any other Serious Adverse Events (SAEs) *(refer to Section 7.1)*

Reports of any other adverse events (AEs) potentially related to Ilaris® therapy that are reported during follow-up will also be collected.

In addition, ALL non-serious adverse events which have been reported since 02-Jul-2012 will also be collected during follow-up.

6.6 Effectiveness/Disease Progression

Information regarding the following aspects of long-term effectiveness (disease progression) will be collected:

- Amyloid A (AA) amyloidosis
- Renal dysfunction
- Sensoneural deafness
- Blindness/vision loss
- Neurologic symptoms
- Growth and development (for children)

[redacted]

Supplemental material BMJ Publishing Group Limited (BMJ) disclaims all liability and responsibility arising from any reliance placed on this supplemental material which has been supplied by the author(s) RMD Open

7 Safety monitoring

All AEs directly observed or reported by the patient or caregiver to the treating physician or other site personnel, from the time of providing informed consent, should be evaluated by the site and assessed for seriousness and relatedness to Ilaris®, as defined below in Section 7.1. All selected AEs (including suspected AEs) described in Section 6.5 and serious AEs will be recorded within the CRF.

7.1 Adverse events

An AE is the appearance or worsening of any undesirable sign, symptom, or medical condition occurring after starting Ilaris® even if the event is not considered to be related to Ilaris®. Medical conditions/diseases present before starting Ilaris® are only considered AEs if they worsen after starting Ilaris®. Abnormal laboratory values or test results constitute AEs only if they induce clinical signs or symptoms, are considered clinically significant, or require therapy. All selected events (serious infections, malignancies, hypersensitivity reactions and vertigo) and ALL non-serious AEs (if reported after 02-Jul-2012) and serious AEs are to be recorded regardless of whether considered related to Ilaris®.

All AEs recorded on the Adverse Events CRF should include the following information:

- The severity grade (mild, moderate, severe)
- Relationship to Ilaris® (suspected/not suspected)
- Duration (start and end dates or if continuing at Registry discontinuation)
- Whether it constitutes a serious adverse event (SAE)

Any treatment of any adverse event should be recorded on the Adverse Event CRF. Some examples of treatment to be recorded are: no action taken (i.e., further observation only); drug of interest dosage adjusted/temporarily interrupted; drug of interest permanently discontinued due to this adverse event; treatment medication adjusted; non-drug therapy given; patient hospitalized/patient's hospitalization prolonged.

Information on all AEs is included in the individual patient eCRFs which must be updated and committed in the study database on a periodic basis but not later than once a month.

An SAE is defined as an event which:

- Is fatal
- Is life-threatening as it occurred
- Results in persistent or significant disability/incapacity
- Constitutes a congenital anomaly/birth defect
- Requires inpatient hospitalization or prolongation of existing hospitalization
- Is medically significant, i.e., defined as an event that jeopardizes the patient or may require medical or surgical intervention to prevent one of the outcomes listed above
- Transmission of infectious agent via medicinal product

Information about adverse reactions observed in relation to Ilaris® can be found in the local product labeling.

[redacted]

7.2 Serious adverse event reporting

To ensure patient safety, all SAEs regardless of relationship to Ilaris® occurring after the patient has provided informed consent and until 30 days after the patient has stopped Registry participation should be reported to Novartis within 24 hours of learning of its occurrence.

SAEs experienced after this 30 day period, should be reported to Novartis at the physician's discretion and in accordance with the physician's normal post-marketing AE spontaneous reporting practices.

Information about all SAEs is collected and recorded on the Adverse Event CRF. The treating physician must also complete an SAE Report Form and send the completed, signed form by fax within 24 hours to the local Novartis Drug Safety and Epidemiology (DS&E) (DS&E). The telephone and fax number of the contact persons in the local DS&E will be provided to each site. Follow-up information should be similarly provided and describe whether the event resolved or continues, if and how it was treated and whether the patient continued or withdrew from the Registry. An SAE that is considered completely unrelated to a previously reported one should be reported separately as a new event. A DS&E associate may require further information from the treating physician for regulatory reporting purposes. SAEs will be reported to the competent authorities and relevant IRB/IEC/REBs in accordance with national and local regulatory requirements in participating countries.

7.3 Pregnancies

To ensure patient safety, each pregnancy in a patient exposed to Ilaris® must be reported to Novartis within 24 hours of learning of its occurrence. The pregnancy should be followed up to determine outcome, including spontaneous or voluntary termination, details of the birth, and the presence or absence of any congenital abnormalities or maternal and/or newborn complications.

Pregnancy should be recorded on a Pregnancy Form and reported by the treating physician to the local Novartis DS&E. Pregnancy follow-up should be recorded on the same form and should include an assessment of the possible relationship to Ilaris of any pregnancy outcome. Any SAE(s) experienced during pregnancy must be reported on an SAE Report Form.

7.4 Adjudication Committees

7.4.1 MAS Adjudication Committee

This section is only applicable for patients already enrolled in this registry study and who are treated with Ilaris® due to a confirmed diagnosis of SJIA.

An independent Adjudication Committee will review and adjudicate information on all suspected cases of macrophage activation syndrome (MAS) in SJIA patients. Cases may be reported by investigators or through systematic database search of specified terms. The MAS Adjudication Committee (MASAC) will review cases as they are identified. A report of the adjudication outcome will be provided to the Sponsor.

As part of the adjudication process, a request for supplemental data collection will be sent to the investigator. The supplemental package will include standardized follow-up questions. If biological specimens (e.g. bone marrow aspirate, bronchoaveolar lavage, etc.) were collected

[redacted]

as part of customary diagnostic workup, samples, such as tissue slides, may be requested by the MASAC for their review and/or for specialized analysis such as immunohistochemical staining for biomarkers associated with MAS.

The MASAC Charter provides detail on the committee composition, adjudication process, database search terms and supplemental data package.

7.4.2 Infection Adjudication Committee

An independent Infection Adjudication Committee (IAC) has been formed on a program level and will review pertinent data from this trial.

The mission of the IAC is to independently review, evaluate and categorize new reports of pre-defined infections during the trial.

The members, detailed mission and procedures of the IAC are detailed in the IAC charter.

7.4.3 Malignancy Adjudication Committee

An independent Malignancy Adjudication Committee (MAC) has been formed on a program level and will review pertinent data from this trial.

The mission of the MAC is to independently review, evaluate and categorize reports of malignancy events across all potential indications and therapeutic areas in the canakinumab development program.

The members, detailed mission and procedures of the MAC are detailed in the MAC Charter.

8 Data acquisition

All data will be collected and entered by the sites directly into the electronic data capture system (EDC). All sites will be fully trained for using the on-line data capture system, including eCRF completion guidelines. Data collected by the treating physician, including data collected from the medical record or directly from patients or their parents/legal representative will be entered into a validated database. All data entry will be conducted so that errors in data completeness, data validity, and data consistency are minimised. There is also an option to collect data on paper case report forms (CRFs) for those sites that cannot complete the forms online. This data will be entered into the database by a designated CRO.

It is the treating physician's responsibility to ensure the accuracy of the data provided to the Registry by any site staff who are trained for Registry data collection.

Initiation and selective monitoring of the participating sites will be performed by a designated CRO. Sites enrolling patients in this study will record data on eCRFs provided by a designated CRO which will capture, check, store and analyze the data. Patient confidentiality will be strictly maintained. CROs will follow their own internal standard operating procedures that have been reviewed and approved by Novartis.

Safety data will be transferred to Novartis as defined in the CRO contract. On a regular basis, when analysis is planned and after quality assurance procedures have been completed, the database will be frozen and a dataset created so that no further changes take place and analysis can be implemented. Clinical data will be transferred to Novartis after closure of the Registry.

[redacted]

8.1 Site monitoring

During the study, a field monitor will visit the site regularly to check the completeness of patient records, the accuracy of entries on the CRFs, the adherence to the protocol and to Good Clinical Practice, and the progress of enrollment. Key study personnel must be available to assist the field monitor during these visits.

The investigator must maintain source documents for each patient in the study, consisting of case and visit notes (hospital or clinic medical records) containing demographic and medical information, and the results of any other tests or assessments. All information on CRFs must be traceable to these source documents in the patient's file. The investigator must also keep the original informed consent form signed by the patient (a signed copy is given to the patient).

The investigator must give the field monitor access to all relevant source documents to confirm their consistency with the CRF entries. Novartis monitoring standards require full verification for the presence of informed consent, adherence to the inclusion/exclusion criteria, documentation of SAEs, and of data that will be used for all primary variables. Additional checks of the consistency of the source data with the CRFs are performed according to the study-specific monitoring plan. No information in source documents about the identity of the patients will be disclosed.

9 Data analysis

The primary purpose of this Registry is to monitor long-term safety of Ilaris®. Additional objectives are to assess the treatment patterns and associated long-term outcomes of Ilaris® therapy prescribed within routine clinical practice. Descriptive statistics will be generated annually after one year of enrolment has been completed. A final analysis will be performed once Registry follow-up is completed.

Concomitant medications entered into the database will be coded using the WHO Drug Reference List, which employs the Anatomical Therapeutic Chemical (ATC) classification system. Medical history/current medical conditions and adverse events will be coded using the Medical dictionary for regulatory activities (MedDRA) terminology.

Statistical analysis of all data will be performed using SAS® statistical software (SAS Institute, Cary, NC, USA). Full analysis details will be provided as part of the statistical analysis plan (SAP).

9.1 Patient demographics/other baseline characteristics

All demographic and disease baseline characteristics (i.e., phenotype/indication for treatment) will be presented using descriptive statistics. Continuous variables will be reported using appropriate measures of dispersion and central tendency (means, medians, ranges and standard deviations) while categorical variables (i.e., gender) will be summarized as number and percentage [proportions and 95% confidence intervals (CI)] of the total Registry population.

[redacted]

9.2 Exposure

Descriptive statistics will be used to describe the number of patients treated with Ilaris throughout the Registry. The number and proportion of patients treated with various initial and subsequent treatments, changes in treatment patterns over time, and discontinuations will be summarized. If possible, events of interest may be stratified by severity, dose, duration of treatment and patient phenotype/indication, dependent upon the completeness of the data.

9.3 Analysis of registry objectives

Descriptive statistics will be used to summarize all safety and effectiveness criteria. No formal hypothesis testing or statistical significance testing is planned. This approach follows the Guidelines for Good Pharmacoepidemiology Practices, Section D, point 10.

The primary analyses will include evaluating the frequency (proportions or rates and 95% CI) of serious infections, malignancies, hypersensitivity reactions and other selected events. Time at risk (in person-years) for select safety outcomes, such as serious infections and malignancies, where possible, will correspond to the induction period associated with the event (if known) and the temporal relationship to treatment with Ilaris, and will be further described in the SAP.

Additional analyses will explore the frequency of markers of disease progression, such as systemic AA amyloidosis, neurologic and ophthalmologic symptoms, and sensorineural deafness, patterns of paediatric growth and development and the frequency of other SAEs considered potentially related to Ilaris®.

9.3.1 Statistical hypothesis, model, and method of analysis

All analyses will be descriptive in nature. There is no predefined hypothesis regarding the magnitude of the effectiveness of Ilaris or the incidence of specific safety outcomes.

9.3.2 Handling of missing values/censoring/discontinuations

Reasonable attempts should be made to limit the amount of missing data related to safety and patient outcomes to ensure that important information related to the primary objective of the registry, evaluation of the long-term safety of Ilaris®, is captured. No imputation will be made on the eCRFs for missing data and further details of how missing data will be handled will be outlined in the SAP. Patients will be censored at Registry termination or early patient discontinuation.

9.3.3 Supportive analyses

As in any product registry, lack of an internal 'untreated' contemporaneous comparator group may pose some challenges for the interpretation of data. In the evaluation of the risk of serious infections, an attempt will be made to make comparisons within the registry population based on treatment status (i.e., timing of exposure in relation to events). However, such an approach is unfeasible when assessing risk of malignancies and appropriate external data sources will be used to aid in the interpretation of the registry data.

[redacted]

9.4 Sample size/power calculation

No formal sample size calculation was used to manage enrolment in the Registry since the goal is to obtain participation by all eligible patients. The estimate over the 5 years of enrolment was a minimum of 200 patients. This estimate has been updated to 260 patients.

9.5 Registry reporting

A final registry report will be prepared at closure of the Registry database, when all data collection procedures are completed. The final report will encompass all planned analyses, including a description of the complete registry population and estimates of events rates, as described above and in the SAP.

In addition, interim annual reports will be generated starting approximately one year after start of enrolment (i.e., Nov-2010). The baseline characteristics of patients will be described. Demographic characteristics (e.g., age, gender) and disease characteristics will be presented using descriptive statistics for continuous variables or proportions for categorical variables. The median and mean follow-up time in the registry will be presented. Analyses of the outcomes of interest will be performed, dependent on the quantity of data available.

10 Ethical considerations

10.1 Regulatory and ethical compliance

The Registry will comply with Volume 9A of The Rules Governing Medicinal Products in the European Union - Guidelines on Pharmacovigilance for Medicinal Products for Human Use. To ensure the quality and integrity of research, this Registry will be conducted under the *Guidelines for Good Pharmacoepidemiology Practices* issued by the International Society for Pharmacoepidemiology (ISPE), the principles outlined in the Declaration of Helsinki, and any applicable national guidelines. Each patient's data collected in the Registry will be stored under an assigned unique Registry number. Data protection and privacy regulations will be observed in capturing, forwarding, processing, and storing patient data. Patients must be informed accordingly, and will be asked to give their consent on data-handling procedures in accordance with national regulations in place in each of the Registry countries.

10.2 Informed consent procedures

Eligible patients may only be included in the Registry after providing written (witnessed, where required by law or regulation), IRB/IEC/REB approved informed consent, or, if incapable of doing so, after such consent has been provided by a legally acceptable representative of the patient. In cases where the patient's representative gives consent, the patient should be informed about the Registry to the extent possible given his/her understanding. If the patient is capable of doing so, he/she should indicate assent by personally signing and dating the written informed consent document or a separate assent form. Informed consent must be obtained before conducting any data collection. The process of obtaining informed consent should be documented in the patient source documents.

Treating physicians will be provided with an informed consent form template that complies with the relevant guidelines and regulatory requirements. Any changes to the proposed

[redacted]

consent form suggested by the treating physician must be agreed to by Novartis/CRO before submission to the IRB/IEC/REB and a copy of the approved version must be provided to Novartis/CRO after IRB/IEC/REB approval.

Women of child bearing potential should be informed that taking Ilaris may involve unknown risks to the fetus if pregnant, and any pregnancy should be reported to her physician as soon as possible.

10.3 Registry governance

A Steering Committee consisting of auto-inflammatory disease/CAPS specialists, an epidemiologist, an infectious disease specialist and a rheumatologist will be assembled to provide scientific guidance on the set-up, conduct, enrolment and analysis of the Registry. The Steering Committee will meet and review data from the Registry at pre-determined intervals as defined by a committee charter. Further, they will review interim data on a regular basis to assess whether any developing safety signals are potentially associated with the use of Ilaris®.

10.4 Responsibilities of the treating physician and IRB/IEC/REB

The protocol and the proposed informed consent form will be reviewed and approved by a properly constituted IRB/IEC/REB before Registry initiation, if required by local law/regulations. Prior to Registry start, the treating physician will be asked to sign a protocol signature page confirming his/her agreement to conduct the study in accordance with the protocol and to give access to all relevant data and records to Novartis and their representatives, IRBs/IEC/REBs, and regulatory authorities, as required. Should the Registry be terminated early for any unanticipated reason, the treating physician will be responsible for informing the IRB/IEC/REB of the early termination.

10.5 Publication of results

Upon Registry completion and finalization of the study report, the results of the Registry may be either submitted for publication and/or posted in a publicly accessible database of results. Publications will follow the International Committee of Medical Journal Editors (ICMJE) guidelines.

Throughout participation in the Registry, individual physicians will have access to their own patients' data and select reports via the EDC system. By written request, physicians will be permitted to publish their data with the consent of the Steering Committee. All participating physicians will be acknowledged in the publication of the final Registry data.

11 Protocol amendments

Changes to the protocol will be documented in written protocol amendments. Major (substantial, significant) amendments will usually require submission to the competent authorities and to the relevant IRB/IEC/REBs for approval or favorable opinion. In such cases, the amendment will be implemented only after approval or favorable opinion has been obtained. Minor (non-substantial) protocol amendments, including administrative changes,

[redacted]

will be filed by Novartis/CRO and at each participating site and submitted to the relevant IRB/IEC/REB or to competent authorities where requested by pertinent regulations.

Any amendment that could have an impact on the patient's agreement to participate in the Registry, e.g., changing the nature of the data collected, requires the patient's informed consent prior to implementation.

12 References

[Agostini L, Martinon F, Burns K, et al (2004)] NALP3 forms an IL-1beta-processing inflammasome with increased activity in Muckle-Wells autoinflammatory disorder. Immunity.; 20(3):319-325.

[Hawkins PN, Lachmann HJ, Aganna E, et al (2004)] Spectrum of clinical features in Muckle-Wells syndrome and response to anakinra. Arthritis Rheum.; 50(2):607-612.

[Hawkins PN, Lachmann HJ, McDermott MF (2003)] Interleukin-1-receptor antagonist in the Muckle-Wells syndrome. N.Engl.J.Med.; 348(25):2583-2584.

[Lequerre T, Vittecoq O, Saugier-Veber P, et al (2007)] A cryopyrin-associated periodic syndrome with joint destruction. Rheumatol.; 46: 709-714.

[Neven B, Prieur A, Quartier dit Maire P (2008)] Cryopyrinopathies: update on pathogenesis and treatment. Nature; 4(9): 481-489.

Declaration of Helsinki 59th WMA General Assembly, Seoul, October 2008. Available at www.wma.net/e/policy/b3.htm

EudraLex VOLUME 9A of The Rules Governing Medicinal Products in the European Union - Guidelines on Pharmacovigilance for Medicinal Products for Human Use. September 2008. Available at http://ec.europa.eu/enterprise/pharmaceuticals/eudralex/vol9_en.htm. Last accessed May 2009.

Guideline on good pharmacovigilance practices (GVP). Module VIII – Post-authorisation safety studies. EMA/330405/2012; 9 July 2012.
Available at: http://www.ema.europa.eu/ema. Last accessed March 2013.

International Committee of medical Journal Editors. Uniform requirements for manuscripts submitted to biomedical journals: writing and editing for biomedical publication. Updated October 2008. Available at: http://www.icmje.org/. Last accessed January 2009.

International Society for Pharmacoepidemiology (ISPE). Guidelines for good pharmacoepidemiology practices (GPP), revision 2. April 2007. Available at http://www.pharmacoepi.org/resources/guidelines_08027.cfm. Last accessed January 2009.

[redacted]
