# Supplementary material for: Long-term safety and effectiveness of canakinumab therapy in patients with cryopyrin-associated periodic syndrome: results from the β-Confident Registry
Source: RMD Open. 2021 May 17;7(2):e001663. doi: 10.1136/rmdopen-2021-001663 (PMC8130749; doi:10.1136/rmdopen-2021-001663)
Supplement: Supplementary data [file rmdopen-2021-001663supp001.pdf]

# ONLINE SUPPLEMENTAL MATERIALS

## Long-term safety and effectiveness of canakinumab therapy in patients with cryopyrin-associated periodic syndrome: results from the β-Confident Registry

Ulrich A Walker[1], Hugh H Tilson[2], Philip N Hawkins[3], Tom van der Poll[4], Stephanie Noviello[5], Jeremy Levy[6], Eleni Vritzali[6], Hal M Hoffman[7,8], Jasmin B Kuemmerle-Deschner[9], on behalf of the CACZ885D2401 Study Investigators

[1]Department of Rheumatology, University Hospital Basel, Basel, Switzerland

[2]Gillings School of Global Public Health, University of North Carolina, Chapel Hill, NC, USA

[3]University College London, London, UK

[4] Amsterdam Medical Center, University of Amsterdam, Amsterdam, The Netherlands

[5]Novartis Pharmaceuticals Corporation, East Hanover, NJ, USA

[6]Novartis Pharma AG, Basel, Switzerland

[7]University of California San Diego, San Diego, CA, USA

[8]Rady Children's Hospital San Diego, San Diego, CA, USA

[9]Division of Paediatric Rheumatology, Department of Paediatrics and Autoinflammation Reference Center Tuebingen, University Hospital Tuebingen, Tuebingen, Germany

**Corresponding author:**

Ulrich A. Walker

Department of Rheumatology, University Hospital Basel,

Basel, Switzerland

Tel:+41612652525

Fax:+41612659021

Email: Ulrich.Walker@usb.ch

**Table S1** Treatment history at enrolment into registry

| | FCAS (N=42) | MWS (N=169) | NOMID (N=32) | All CAPS (N=243) | Other* (N=42) | All (N=285) |
|---|---|---|---|---|---|---|
| IL-1 inhibitor including canakinumab (clinical trial or marketed) | 35 (83.3) | 152 (89.9) | 29 (90.6) | 216 (88.9) | 36 (85.7) | 252 (88.4) |
| IL-1 inhibitor including canakinumab in a clinical trial | 28 (66.7) | 112 (66.3) | 25 (78.1) | 165 (67.9) | 24 (57.1) | 189 (66.3) |
| Canakinumab prior to enrolment | 34 (81.0) | 134 (79.3) | 21 (65.6) | 189 (77.8) | 27 (64.3) | 216 (75.8) |
| Canakinumab in a clinical trial | 24 (57.1) | 71 (42.0) | 9 (28.1) | 104 (42.8) | 0 | 104 (36.5) |
| Marketed canakinumab only | 10 (23.8) | 63 (37.3) | 12 (37.5) | 85 (35.0) | 27 (64.3) | 112 (39.3) |
| Other IL-1 inhibitor | 16 (38.1) | 77 (45.6) | 24 (75.0) | 117 (48.1) | 24 (57.1) | 141 (49.5) |
| Other auto-inflammatory disease medication (excluding IL-1 inhibitor) | 19 (45.2) | 70 (41.4) | 19 (59.4) | 108 (44.4) | 29 (69.0) | 137 (48.1) |
| Corticosteroids | 7 (16.7) | 42 (24.9) | 15 (46.9) | 64 (26.3) | 24 (57.1) | 88 (30.9) |
| NSAIDs | 14 (33.3) | 49 (29.0) | 10 (31.3) | 73 (30.0) | 14 (33.3) | 87 (30.5) |
| csDMARD | 1 (2.4) | 15 (8.9) | 8 (25.0) | 24 (9.9) | 14 (33.3) | 38 (13.3) |
| Anti-TNF agent | 0 | 6 (3.6) | 5 (15.6) | 11 (4.5) | 9 (21.4) | 20 (7.0) |

| | | | | | | |
|---|---|---|---|---|---|---|
| IL-6 inhibitor | 0 | 1 (0.6) | 0 | 1 (0.4) | 1 (2.4) | 2 (0.7) |
| Other | 3 (7.1) | 9 (5.3) | 1 (3.1) | 13 (5.3) | 12 (28.6) | 25 (8.8) |

Values are n (%) unless stated otherwise

*Other includes atypical CAPS (n=18) and non-CAPS diagnoses (n=24); non-CAPS diagnoses include systemic juvenile idiopathic arthritis, unspecified autoinflammatory syndromes, familial Mediterranean fever, mevalonate kinase deficiency, adult-onset Still's disease, tumour necrosis factor receptor-associated periodic syndrome, Erdheim–Chester disease, Blau syndrome and granulomatosis with polyangiitis.

Canakinumab prior to enrolment refers to canakinumab use at least 28 days before baseline (date of inform consent); other inflammatory inhibitors includes antihistamine, colchicine, dapsone, gold, mesalazine, mycophenolate, tegeline and thalidomide.

csDMARD, conventional synthetic disease modifying rheumatic drugs encompassing methotrexate, azathioprine, cyclophosphamide, and mycophenolic acid/mycophenolate mofetil.

CAPS, cryopyrin-associated periodic syndromes; csDMARD, conventional synthetic disease-modifying antirheumatic drug; FCAS, familial cold autoinflammatory syndrome; IL, interleukin; MWS, Muckle–Wells syndrome; NOMID, neonatal-onset multisystem inflammatory disease; NSAID, non-steroidal anti-inflammatory drug; TNF, tumour necrosis factor.

**Table S2** Baseline canakinumab dose by phenotype

| Dose categories (mg/kg) | FCAS (N=42) | MWS (N=169) | NOMID (N=32) | All CAPS (N=243) | Other* (N=42) | All (N=285) |
|---|---|---|---|---|---|---|
| **<2 mg/kg** | 16 (38.1) | 43 (25.4) | 2 (6.3) | 61 (25.1) | 9 (21.4) | 70 (24.6) |
| **2–<4 mg/kg** | 22 (52.4) | 83 (49.1) | 18 (56.3) | 123 (50.6) | 24 (57.1) | 147 (51.6) |
| **4–<8 mg/kg** | 0 | 21 (12.4) | 8 (25.0) | 29 (11.9) | 4 (9.5) | 33 (11.6) |
| **>=8 mg/kg** | 0 | 1 (0.6) | 3 (9.4) | 4 (1.6) | 0 | 4 (1.4) |

Values are n (%) unless stated otherwise

*Other includes atypical CAPS (n=18) and non-CAPS diagnoses (n=24); non-CAPS diagnoses include systemic juvenile idiopathic arthritis, unspecified autoinflammatory syndromes, familial Mediterranean fever, mevalonate kinase deficiency, adult-onset Still's disease, tumour necrosis factor receptor-associated periodic syndrome, Erdheim–Chester disease, Blau syndrome and granulomatosis with polyangiitis.

CAPS, cryopyrin-associated periodic syndromes; FCAS, familial cold autoinflammatory syndrome; MWS, Muckle–Wells syndrome; NOMID, neonatal-onset multisystem inflammatory disease.

**Table S3** Changes in audiogram, ophthalmological assessments, and brain MRI from baseline to last available assessment

| | **FCAS (N=42)** | **MWS (N=169)** | **NOMID (N=32)** | **All CAPS (N=243)** | **Other* (N=42)** | **All (N=285)** |
|---|---|---|---|---|---|---|
| **Abnormal audiogram, % (m/n)** | | | | | | |
| Baseline | 14.3 (3/21) | 57.0 (69/121) | 66.7 (14/21) | 52.8 (86/163) | 50.0 (2/4) | 52.7 (88/167) |
| Last available assessment | 0.0 (0/11) | 57.4 (66/115) | 66.7 (14/21) | 54.4 (80/147) | 25.0 (2/8) | 52.9 (82/155) |
| **Abnormal ophthalmologic assessment, % (m/n)** | | | | | | |
| Baseline | 11.1 (2/18) | 24.7 (20/81) | 50.0 (10/20) | 26.9 (32/119) | 25.0 (2/8) | 26.8 (34/127) |
| Last available assessment | 0.0 (0/2) | 15.9 (14/88) | 57.1 (12/21) | 23.4 (26/111) | 15.4 (2/13) | 22.6 (28/124) |
| **Abnormal brain MRI assessment, % (m/n)** | | | | | | |
| Baseline | 20.0 (3/15) | 14.0 (6/43) | 37.5 (6/16) | 20.3 (15/74) | 0 (0/2) | 19.7 (15/76) |
| Last available assessment | 0.0 (0/6) | 11.4 (4/35) | 35.7 (5/14) | 16.4 (9/55) | 0 (0/6) | 14.8 (9/61) |

% values are calculated based on the number of patients with available data at the time of assessment (m/n)

*Other includes atypical CAPS (n=18) and non-CAPS diagnoses (n=24); non-CAPS diagnoses include systemic juvenile idiopathic arthritis, unspecified autoinflammatory syndromes, familial Mediterranean fever, mevalonate kinase deficiency, adult-onset Still's disease, tumour necrosis factor receptor-associated periodic syndrome, Erdheim–Chester disease, Blau syndrome and granulomatosis with polyangiitis.

CAPS, cryopyrin-associated periodic syndromes; FCAS, familial cold autoinflammatory syndrome; m, number of patients who reported disease characteristic; MRI, magnetic resonance imaging; MWS, Muckle–Wells syndrome; n, number of patients with available data in subgroup; NOMID, neonatal-onset multisystem inflammatory disease.

**Table S4** Delay in sexual maturation and neurocognitive function at baseline and last available assessment

| | FCAS (N=8) | MWS (N=44) | NOMID (N=14) | All CAPS (N=66) | Other* (N=17) | All (N=83) |
|---|---|---|---|---|---|---|
| **Delay in sexual maturation, % (m/n)** | | | | | | |
| Baseline | 0.0 (0/6) | 2.9 (1/34) | 12.5 (1/8) | 4.2 (2/48) | 16.7 (2/12) | 6.7 (4/60) |
| Last available assessment | 0.0 (0/5) | 0.0 (0/35) | 0.0 (0/9) | 0.0 (0/49) | 14.3 (2/14) | 3.2 (2/63) |
| **Delay in neurocognitive function, % (m/n)** | | | | | | |
| Baseline | 0.0 (0/7) | 2.4 (1/41) | 45.5 (5/11) | 10.2 (6/59) | 0.0 (0/15) | 8.1 (6/74) |
| Last available assessment | 0.0 (0/6) | 5.1 (2/39) | 30.8 (4/13) | 10.3 (6/58) | 0.0 (0/16) | 8.1 (6/74) |

% values are calculated based on the number of patients with available data at the time of assessment (m/n); data were collected only from patients aged ≥6 and <18 years

m = number of patients who reported disease characteristic; n = number of patients with available data in subgroup

*Other includes atypical CAPS (n=18) and non-CAPS diagnoses (n=24); non-CAPS diagnoses include systemic juvenile idiopathic arthritis, unspecified autoinflammatory syndromes, familial Mediterranean fever, mevalonate kinase deficiency, adult-onset Still's disease, tumour necrosis factor-receptor associated periodic syndrome, Erdheim-Chester disease, Blau syndrome, and granulomatosis with polyangiitis.

CAPS, cryopyrin-associated periodic syndromes; FCAS, familial cold autoinflammatory syndrome; MWS, Muckle–Wells syndrome; NOMID, neonatal-onset multisystem inflammatory disease.

**Table S5** Adverse events by phenotypes

| SOC, Preferred term, n (IR/ 100 PY) (95% CI) | FCAS N= 42 | MWS N=169 | NOMID N= 32 | All CAPS N=243 | Other* N= 42 | ALL N=285 |
|---|---|---|---|---|---|---|
| **All events** | 114 (73.1) [60.3, 87.8] | 681 (105.0) [97.2, 113.2] | 119 (104.6) [86.6, 125.2] | 914 (99.5) [93.2, 106.2] | 200 (143.1) [124.0-164.4] | 1114 (105.3) [99.2, 111.6] |
| **Infections and Infestations** | 54 (34.6) [26.0, 45.2] | 244 (37.6) [33.0, 42.6] | 45 (39.6) [28.8, 52.9] | 343 (37.3) [33.5, 41.5] | 59 (42.2) [32.1, 54.5] | 402 (38.0) [34.4- 41.9] |
| Nasopharyngitis | 13 (8.3) [4.4, 14.3] | 69 (10.6) [8.3, 13.5] | 11 (9.7) [4.8, 17.3] | 93 (10.1) [8.2, 12.4] | 7 (5.0) [2.0, 10.3] | 100 (9.4) [7.7, 11.5] |
| Gastroenteritis | 6 (3.8) [1.4, 8.4] | 13 (2.0) [1.1, 3.4] | 1 (0.9) [0.0, 4.9] | 20 (2.2) [1.3, 3.4] | 6 (4.3) [1.6, 9.3] | 26 (2.5) [1.6, 3.6] |
| Influenza | 6 (3.8) [1.4, 8.4] | 8 (1.2) [0.5, 2.4] | 2 (1.8) [0.2, 6.3] | 16 (1.7) [1.0, 2.8] | 4 (2.9) [0.8, 7.3] | 20 (1.9) [1.2, 2.9] |
| Tonsillitis | 2 (1.3) [0.2, 4.6] | 11 (1.7) [0.8, 3.0] | 1 (0.9) [0.0, 4.9] | 14 (1.5) [0.8, 2.6] | 5 (3.6) [1.3, 8.3] | 19 (1.8) [1.2, 2.8] |
| Urinary tract infection | 3 (1.9) [0.4, 5.6] | 13 (2.0) [1.1, 3.4] | 2 (1.8) [0.2, 6.3] | 18 (2.0) [1.2, 3.1] | 0 [0.0, 2.7] | 18 (1.7) [1.0, 2.7] |
| Pharyngitis | 0 [0.0, 2.4] | 12 (1.8) [1.0, 3.2] | 2 (1.8) [0.2, 6.3] | 14 (1.5) [0.8, 2.6] | 2 (1.4) [0.2, 5.2] | 16 (1.5) [0.9, 2.5] |
| Bronchitis | 1 (0.6) [0.0, 3.6] | 8 (1.2) [0.5, 2.4] | 2 (1.8) [0.2, 6.3] | 11 (1.2) [0.6, 2.1] | 3 (2.1) [0.4, 6.3] | 14 (1.3) [0.7, 2.2] |
| Lower respiratory tract infection | 5 (3.2) [1.0, 7.5] | 4 (0.6) [0.2, 1.6] | 5 (4.4) [1.4,10.3] | 14 (1.5) [0.8, 2.6] | 0 [0.0, 2.7] | 14 (1.3) [0.7, 2.2] |
| Viral infection | 2 (1.3) [0.2, 4.6] | 6 (0.9) [0.3, 2.0] | 1 (0.9) [0.0, 4.9] | 9 (1.0) [0.4, 1.9] | 2 (1.4) [0.2, 5.2] | 11 (1.0) [0.5, 1.9] |
| Ear infection | 3 (1.9) [0.4, 5.6] | 5 (0.8) [0.3, 1.8] | 0 [0.0, 3.2] | 8 (0.9) [0.4, 1.7] | 1 (0.7) [0.0, 4.0] | 9 (0.9) [0.4, 1.6] |
| Oral herpes | 5 (3.2) [1.0, 7.5] | 3 (0.5) [0.1, 1.4] | 0 [0.0, 3.2] | 8 (0.9) [0.4, 1.7] | 0 [0.0, 2.7] | 8 (0.8) [0.3, 1.5] |
| Rhinitis | 0 [0.0, 2.4] | 5 (0.8) [0.3, 1.8] | 2 (1.8) [0.2, 6.3] | 7 (0.8) [0.3, 1.6] | 1 (0.7) [0.0, 4.0] | 8 (0.8) [0.3, 1.5] |

| SOC, Preferred term, n (IR/ 100 PY) (95% CI) | FCAS N= 42 | MWS N=169 | NOMID N= 32 | All CAPS N=243 | Other* N= 42 | ALL N=285 |
|---|---|---|---|---|---|---|
| Pneumonia | 0 [0.0, 2.4] | 5 (0.8) [0.3, 1.8] | 0 [0.0, 3.2] | 5 (0.5) [0.2, 1.3] | 2 (1.4) [0.2, 5.2] | 7 (0.7) [0.3, 1.4] |
| Sinusitis | 0 [0.0, 2.4] | 3 (0.5) [0.1, 1.4] | 1 (0.9) [0.0, 5.0] | 4 (0.4) [0.1, 1.1] | 3 (2.2) [0.4, 6.3] | 7 (0.7) [0.3, 1.4] |
| Herpes zoster | 0 [0.0, 2.4] | 5 (0.8) [0.3, 1.8] | 0 [0.0, 3.2] | 5 (0.5) [0.2, 1.3] | 1 (0.7) [0.0, 4.0] | 6 (0.6) [0.2, 1.2] |
| Upper respiratory tract infection | 0 [0.0, 2.4] | 3 (0.5) [0.1, 1.4] | 0 [0.0, 3.2] | 3 (0.3) [0.1, 1.0] | 3 (2.1) [0.4, 6.3] | 6 (0.6) [0.2, 1.2] |
| Laryngitis | 1 (0.6) [0.0, 3.6] | 4 (0.6) [0.2, 1.6] | 0 [0.0, 3.2] | 5 (0.5) [0.2, 1.3] | 0 [0.0, 2.7] | 5 (0.5) [0.2, 1.1] |
| Respiratory tract infection | 0 [0.0, 2.4] | 2 (0.3) [0.0, 1.1] | 1 (0.9) [0.0, 4.9] | 3 (0.3) [0.1, 1.0] | 2 (1.4) [0.2, 5.2] | 5 (0.5) [0.2, 1.1] |
| Viral upper respiratory tract infection | 1 (0.6) [0.0, 3.6] | 2 (0.3) [0.0, 1.1] | 2 (1.8) [0.2, 6.3] | 5 (0.5) [0.2, 1.3] | 0 [0.0, 2.7] | 5 (0.5) [0.2, 1.1] |
| Folliculitis | 0 [0.0, 2.4] | 4 (0.6) [0.2, 1.6] | 0 [0.0, 3.2] | 4 (0.4) [0.1, 1.1] | 0 [0.0, 2.7] | 4 (0.4) [0.1, 1.0] |
| Cellulitis | 0 [0.0, 2.4] | 2 (0.3) [0.0, 1.1] | 1 (0.9) [0.0, 4.9] | 3 (0.3) [0.1, 1.0] | 0 [0.0, 2.7] | 3 (0.3) [0.1, 0.8] |
| Conjunctivitis | 0 [0.0, 2.4] | 2 (0.3) [0.0, 1.1] | 1 (0.9) [0.0, 4.9] | 3 (0.3) [0.1, 1.0] | 0 [0.0, 2.7] | 3 (0.3) [0.1, 0.8] |
| Cystitis | 0 [0.0, 2.4] | 1 (0.2) [0.0, 0.9] | 2 (1.8) [0.2, 6.3] | 3 (0.3) [0.1, 1.0] | 0 [0.0, 2.7] | 3 (0.3) [0.1, 0.8] |
| Herpes virus infection | 0 [0.0, 2.4] | 2 (0.3) [0.0, 1.1] | 1 (0.9) [0.2, 4.9] | 3 (0.3) [0.1, 1.0] | 0 [0.0, 2.7] | 3 (0.3) [0.1, 0.8] |
| Hordeolum | 0 [0.0, 2.4] | 3 (0.5) [0.1, 1.4] | 0 [0.0, 3.2] | 3 (0.3) [0.1, 1.0] | 0 [0.0, 2.7] | 3 (0.3) [0.1, 0.8] |
| Onychomycosis | 0 [0.0, 2.4] | 1 (0.2) [0.0, 0.9] | 0 [0.0, 3.2] | 1 (0.1) [0.0, 0.6] | 2 (1.4) [0.2, 5.2] | 3 (0.3) [0.1, 0.8] |
| Otitis externa | 0 [0.0, 2.4] | 3 (0.5) [0.1, 1.4] | 0 [0.0, 3.2] | 3 (0.3) [0.1, 1.0] | 0 [0.0, 2.7] | 3 (0.3) [0.1, 0.8] |

| SOC, Preferred term, n (IR/ 100 PY) (95% CI) | FCAS N= 42 | MWS N=169 | NOMID N= 32 | All CAPS N=243 | Other* N= 42 | ALL N=285 |
|---|---|---|---|---|---|---|
| Pharyngitis streptococcal | 0 [0.0, 2.4] | 3 (0.5) [0.1, 1.4] | 0 [0.0, 3.2] | 3 (0.3) [0.1, 1.0] | 0 [0.0, 2.7] | 3 (0.3) [0.1, 0.8] |
| Pyelonephritis | 0 [0.0, 2.4] | 2 (0.3) [0.0, 1.1] | 0 [0.0, 3.2] | 2 (0.2) [0.0, 0.8] | 1(0.7) [0.0, 4.0] | 3 (0.3) [0.1, 0.8] |
| Varicella | 0 [0.0, 2.4] | 2 (0.3) [0.0, 1.1] | 0 [0.0, 3.2] | 2 (0.2) [0.0, 0.8] | 1 (0.7) [0.0, 4.0] | 3 (0.3) [0.1, 0.8] |
| Abscess | 0 [0.0, 2.4] | 2 (0.3) [0.0, 1.1] | 0 [0.0, 3.2] | 2 (0.2) [0.0, 0.8] | 0 [0.0, 2.7] | 2 (0.2) [0.0, 0.7] |
| Campylobacter infection | 2 (1.3) [0.2, 4.6] | 0 [0.0, 0.6] | 0 [0.0, 3.2] | 2 (0.2) [0.0, 0.8] | 0 [0.0, 2.7] | 2 (0.2) [0.0, 0.7] |
| Gastroenteritis viral | 0 [0.0, 2.4] | 2 (0.3) [0.0, 1.1] | 0 [0.0, 3.2] | 2 (0.2) [0.0, 0.8] | 0 [0.0, 2.7] | 2 (0.2) [0.0, 0.7] |
| Gingivitis | 0 [0.0, 2.4] | 2 (0.3) [0.0, 1.1] | 0 [0.0, 3.2] | 2 (0.2) [0.0, 0.8] | 0 [0.0, 2.7] | 2 (0.2) [0.0, 0.7] |
| Impetigo | 0 [0.0, 2.4] | 1 (0.2) [0.0, 0.9] | 0 [0.0, 3.2] | 1 (0.1) [0.0, 0.6] | 1 (0.7) [0.0, 4.0] | 2 (0.2) [0.0, 0.7] |
| Infection susceptibility increased | 0 [0.0, 2.4] | 2 (0.3) [0.0, 1.1] | 0 [0.0, 3.2] | 2 (0.2) [0.0, 0.8] | 0 [0.0, 2.7] | 2 (0.2) [0.0, 0.7] |
| Meningitis | 0 [0.0, 2.4] | 0 [0.0, 0.6] | 2 (1.8) [0.1, 6.4] | 2 (0.2) [0.0, 0.8] | 0 [0.0, 2.7] | 2 (0.2) [0.0, 0.7] |
| Perirectal abscess | 0 [0.0, 2.4] | 2 (0.3) [0.0, 1.1] | 0 [0.0, 3.2] | 2 (0.2) [0.0, 0.8] | 0 [0.0, 2.7] | 2 (0.2) [0.0, 0.7] |
| Skin infection | 0 [0.0, 2.4] | 2 (0.3) [0.0, 1.1] | 0 [0.0, 3.2] | 2 (0.2) [0.0, 0.8] | 0 [0.0, 2.7] | 2 (0.2) [0.0, 0.7] |
| Tooth abscess | 2 (1.3) [0.2, 4.6] | 0 [0.0, 0.6] | 0 [0.0, 3.2] | 2 (0.2) [0.0, 0.8] | 0 [0.0, 2.7] | 2 (0.2) [0.0, 0.7] |
| Tooth infection | 0 [0.0, 2.4] | 2 (0.3) [0.0, 1.1] | 0 [0.0, 3.2] | 2 (0.2) [0.0, 0.8] | 0 [0.0, 2.7] | 2 (0.2) [0.0, 0.7] |
| Viral rash | 0 [0.0, 2.4] | 2 (0.3) [0.0, 1.1] | 0 [0.0, 3.2] | 2 (0.2) [0.0, 0.8] | 0 [0.0, 2.7] | 2 (0.2) [0.0, 0.7] |

| SOC, Preferred term, n (IR/ 100 PY) (95% CI) | FCAS N= 42 | MWS N=169 | NOMID N= 32 | All CAPS N=243 | Other* N= 42 | ALL N=285 |
|---|---|---|---|---|---|---|
| Vulvovaginal candidiasis | 1 (0.6) [0.0, 3.6] | 1 (0.2) [0.0, 0.9] | 0 [0.0, 3.2] | 2 (0.2) [0.0, 0.8] | 0 [0.0, 2.7] | 2 (0.2) [0.0, 0.7] |
| Abscess jaw | 0 [0.0, 2.4] | 1 (0.2) [0.0, 0.9] | 0 [0.0, 3.2] | 1 (0.1) [0.0, 0.6] | 0 [0.0, 2.7] | 1 (0.1) [0.0, 0.5] |
| Abscess limb | 0 [0.0, 2.4] | 1 (0.2) [0.0, 0.9] | 0 [0.0, 3.2] | 1 (0.1) [0.0, 0.6] | 0 [0.0, 2.7] | 1 (0.1) [0.0, 0.5] |
| Acne pustular | 0 [0.0, 2.4] | 1 (0.2) [0.0, 0.9] | 0 [0.0, 3.2] | 1 (0.1) [0.0, 0.6] | 0 [0.0, 2.7] | 1 (0.1) [0.0, 0.5] |
| Anal abscess | 0 [0.0, 2.4] | 1 (0.2) [0.0, 0.9] | 0 [0.0, 3.2] | 1 (0.1) [0.0, 0.6] | 0 [0.0, 2.7] | 1 (0.1) [0.0, 0.5] |
| Appendicitis | 0 [0.0, 2.4] | 1 (0.2) [0.0, 0.9] | 0 [0.0, 3.2] | 1 (0.1) [0.0, 0.6] | 0 [0.0, 2.7] | 1 (0.1) [0.0, 0.5] |
| Bacterial infection | 0 [0.0, 2.4] | 1 (0.2) [0.0, 0.9] | 0 [0.0, 3.2] | 1 (0.1) [0.0, 0.6] | 0 [0.0, 2.7] | 1 (0.1) [0.0, 0.5] |
| Bacterial tracheitis | 0 [0.0, 2.4] | 1 (0.2) [0.0, 0.9] | 0 [0.0, 3.2] | 1 (0.1) [0.0, 0.6] | 0 [0.0, 2.7] | 1 (0.1) [0.0, 0.5] |
| Cellulitis staphylococcal | 0 [0.0, 2.4] | 0 [0.0, 0.6] | 0 [0.0, 3.2] | 0 [0.0, 0.4] | 1 (0.7) [0.0, 4.0] | 1 (0.1) [0.0, 0.5] |
| Chronic sinusitis | 0 [0.0, 2.4] | 1 (0.2) [0.0, 0.9] | 0 [0.0, 3.2] | 1 (0.1) [0.0, 0.6] | 0 [0.0, 2.7] | 1 (0.1) [0.0, 0.5] |
| Diverticulitis | 0 [0.0, 2.4] | 1 (0.2) [0.0, 0.9] | 0 [0.0, 3.2] | 1 (0.1) [0.0, 0.6] | 0 [0.0, 2.7] | 1 (0.1) [0.0, 0.5] |
| Epstein-Barr virus infection | 0 [0.0, 2.4] | 0 [0.0, 0.6] | 0 [0.0, 3.2] | 0 (0) [0.0, 0.4] | 1 (0.7) [0.0, 4.0] | 1 (0.1) [0.0, 0.5] |
| Erysipelas | 0 [0.0, 2.4] | 0 [0.0, 0.6] | 1 (0.9) [0.0, 4.9] | 1 (0.1) [0.0, 0.6] | 0 [0.0, 2.7] | 1 (0.1) [0.0, 0.5] |
| Eyelid infection | 0 [0.0, 2.4] | 0 [0.0, 0.6] | 0 [0.0, 3.2] | 0 (0) [0.0, 0.4] | 1 (0.7) [0.0, 4.0] | 1 (0.1) [0.0, 0.5] |
| Furuncle | 0 [0.0, 2.4] | 1 (0.2) [0.0, 0.9] | 0 [0.0, 3.2] | 1 (0.1) [0.0, 0.6] | 0 [0.0, 2.7] | 1 (0.1) [0.0, 0.5] |
| Gastroenteritis rotavirus | 0 [0.0, 2.4] | 0 [0.0, 0.6] | 0 [0.0, 3.2] | 0 [0.0, 0.4] | 1 (0.7) [0.0, 4.0] | 1 (0.1) [0.0, 0.5] |

| SOC, Preferred term, n (IR/ 100 PY) (95% CI) | FCAS N= 42 | MWS N=169 | NOMID N= 32 | All CAPS N=243 | Other* N= 42 | ALL N=285 |
|---|---|---|---|---|---|---|
| Latent tuberculosis | 0 [0.0, 2.4] | 0 [0.0, 0.6] | 0 [0.0, 3.2] | 0 [0.0, 0.4] | 1 (0.7) [0.0, 4.0] | 1 (0.1) [0.0, 0.5] |
| Localized infection | 0 [0.0, 2.4] | 0 [0.0, 0.6] | 1 (0.9) [0.0, 4.9] | 1 (0.1) [0.0, 0.6] | 0 [0.0, 2.7] | 1 (0.1) [0.0, 0.5] |
| Lung infection | 0 [0.0, 2.4] | 1 (0.2) [0.0, 0.9] | 0 [0.0, 3.2] | 1 (0.1) [0.0, 0.6] | 0 [0.0, 2.7] | 1 (0.1) [0.0, 0.5] |
| Mastitis | 1 (0.6) [0.0, 3.6] | 0 [0.0, 0.6] | 0 [0.0, 3.2] | 1 (0.1) [0.0, 0.6] | 0 [0.0, 2.7] | 1 (0.1) [0.0, 0.5] |
| Meningitis aseptic | 0 [0.0, 2.4] | 0 [0.0, 0.6] | 1 (0.9) [0.0, 4.9] | 1 (0.1) [0.0, 0.6] | 0 [0.0, 2.7] | 1 (0.1) [0.0, 0.5] |
| Meningitis bacterial | 0 [0.0, 2.4] | 1 (0.2) [0.0, 0.9] | 0 [0.0, 3.2] | 1 (0.1) [0.0, 0.6] | 0 [0.0, 2.7] | 1 (0.1) [0.0, 0.5] |
| Molluscum contagiosum | 0 [0.0, 2.4] | 1 (0.2) [0.0, 0.9] | 0 [0.0, 3.2] | 1 (0.1) [0.0, 0.6] | 0 [0.0, 2.7] | 1 (0.1) [0.0, 0.5] |
| Otitis media | 0 [0.0, 2.4] | 1 (0.2) [0.0, 0.9] | 0 [0.0, 3.2] | 1 (0.1) [0.0, 0.6] | 0 [0.0, 2.7] | 1 (0.1) [0.0, 0.5] |
| Parvovirus B19 infection | 0 [0.0, 2.4] | 0 [0.0, 0.6] | 0 [0.0, 3.2] | 0 [0.0, 0.4] | 1 (0.7) [0.0, 4.0] | 1 (0.1) [0.0, 0.5] |
| Periorbital cellulitis | 0 [0.0, 2.4] | 1 (0.2) [0.0, 0.9] | 0 [0.0, 3.2] | 1 (0.1) [0.0, 0.6] | 0 [0.0, 2.7] | 1 (0.1) [0.0, 0.5] |
| Pilonidal cyst | 0 [0.0, 2.4] | 0 [0.0, 0.6] | 1 (0.9) [0.0, 4.9] | 1 (0.1) [0.0, 0.6] | 0 [0.0, 2.7] | 1 (0.1) [0.0, 0.5] |
| Postoperative wound infection | 0 [0.0, 2.4] | 0 [0.0, 0.6] | 0 [0.0, 3.2] | 0 [0.0, 0.4] | 1 (0.7) [0.0, 4.0] | 1 (0.1) [0.0, 0.5] |
| Pyelonephritis acute | 0 [0.0, 2.4] | 0 [0.0, 0.6] | 0 [0.0, 3.2] | 0 [0.0, 0.4] | 1 (0.7) [0.0, 4.0] | 1 (0.1) [0.0, 0.5] |
| Rhinovirus infection | 0 [0.0, 2.4] | 1 (0.2) [0.0, 0.9] | 0 [0.0, 3.2] | 1 (0.1) [0.0, 0.6] | 0 [0.0, 2.7] | 1 (0.1) [0.0, 0.5] |
| Scarlet fever | 0 [0.0, 2.4] | 0 [0.0, 0.6] | 0 [0.0, 3.2] | 0 [0.0, 0.4] | 1 (0.7) [0.0, 4.0] | 1 (0.1) [0.0, 0.5] |
| Staphylococcal infection | 0 [0.0, 2.4] | 1 (0.2) [0.0, 0.9] | 0 [0.0, 3.2] | 1 (0.1) [0.0, 0.6] | 0 [0.0, 2.7] | 1 (0.1) [0.0, 0.5] |

| SOC, Preferred term, n (IR/ 100 PY) (95% CI) | FCAS N= 42 | MWS N=169 | NOMID N= 32 | All CAPS N=243 | Other* N= 42 | ALL N=285 |
|---|---|---|---|---|---|---|
| Subcutaneous abscess | 0 [0.0, 2.4] | 1 (0.2) [0.0, 0.9] | 0 [0.0, 3.2] | 1 (0.1) [0.0, 0.6] | 0 [0.0, 2.7] | 1 (0.1) [0.0, 0.5] |
| Tinea cruris | 0 [0.0, 2.4] | 0 [0.0, 0.6] | 1 (0.9) [0.0, 4.9] | 1 (0.1) [0.0, 0.6] | 0 [0.0, 2.7] | 1 (0.1) [0.0, 0.5] |
| Tracheitis | 0 [0.0, 2.4] | 0 [0.0, 0.6] | 0 [0.0, 3.2] | 0 [0.0, 0.4] | 1 (0.7) [0.0, 4.0] | 1 (0.1) [0.0, 0.5] |
| Vaccination site cellulitis | 0 [0.0, 2.4] | 1 (0.2) [0.0, 0.9] | 0 [0.0, 3.2] | 1 (0.1) [0.0, 0.6] | 0 [0.0, 2.7] | 1 (0.1) [0.0, 0.5] |
| Viral diarrhoea | 0 [0.0, 2.4] | 0 [0.0, 0.6] | 0 [0.0, 3.2] | 0 [0.0, 0.4] | 1 (0.7) [0.0, 4.0] | 1 (0.1) [0.0, 0.5] |
| Viral myositis | 0 [0.0, 2.4] | 0 [0.0, 0.6] | 0 [0.0, 3.2] | 0 [0.0, 0.4] | 1 (0.7) [0.0, 4.0] | 1 (0.1) [0.0, 0.5] |
| Wound infection | 0 [0.0, 2.4] | 1 (0.2) [0.0, 0.9] | 0 [0.0, 3.2] | 1 (0.1) [0.0, 0.6] | 0 [0.0, 2.7] | 1 (0.1) [0.0, 0.5] |
| **General disorders and administration site conditions** | 4 (2.6) [0.7, 6.6] | 62 (9.6) [7.3, 12.3] | 10 (8.8) [4.2, 16.2] | 76 (8.3) [6.5, 10.4] | 20 (14.3) [8.7, 22.1] | 96 (9.1) [ 7.4, 11.1] |
| Pyrexia | 2 (1.3) [0.2, 4.6] | 8 (1.2) [0.5, 2.4] | 2 (1.8) [0.2, 6.3] | 12 (1.3) [0.7, 2.3] | 12 (8.6) [4.4, 15.0] | 24 (2.3) [1.5, 3.4] |
| Fatigue | 1 (0.6) [0.0, 3.6] | 15 (2.3) [1.3, 3.8] | 5 (4.4) [1.4,10.3] | 21 (2.3) [1.4, 3.5] | 2 (1.4) [0.2, 5.2] | 23 (2.2) [1.4, 3.3] |
| Malaise | 0 [0.0, 2.4] | 4 (0.6) [0.2, 1.6] | 0 [0.0, 3.2] | 4 (0.4) [0.1, 1.1] | 2 (1.4) [0.2, 5.2] | 6 (0.6) [0.2, 1.2] |
| Vaccination site inflammation | 0 [0.0, 2.4] | 5 (0.8) [0.3, 1.8] | 0 [0.0, 3.2] | 5 (0.5) [0.2, 1.3] | 1 (0.7) [0.0, 4.0] | 6 (0.6) [0.2, 1.2] |
| Condition aggravated | 0 [0.0, 2.4] | 2 (0.3) [0.0, 1.1] | 1 (0.9) [0.0, 4.9] | 3 (0.3) [0.1, 1.0] | 0 [0.0, 2.7] | 3 (0.3) [0.1, 0.8] |
| Cyst | 0 [0.0, 2.4] | 3 (0.5) [0.1, 1.4] | 0 [0.0, 3.2] | 3 (0.3) [0.1, 1.0] | 0 [0.0, 2.7] | 3 (0.3) [0.1, 0.8] |
| Influenza like illness | 1 (0.6) [0.0, 3.4] | 2 (0.3) [0.0, 1.1] | 0 [0.0, 3.2] | 3 (0.3) [0.0, 1.0] | 0 [0.0, 2.7] | 3 (0.3) [0.1, 1.0] |

| SOC, Preferred term, n (IR/ 100 PY) (95% CI) | FCAS N= 42 | MWS N=169 | NOMID N= 32 | All CAPS N=243 | Other* N= 42 | ALL N=285 |
|---|---|---|---|---|---|---|
| Injection site pain | 0 [0.0, 2.4] | 2 (0.3) [0.0, 1.1] | 0 [0.0, 3.2] | 2 (0.2) [0.0, 0.8] | 1 (0.7) [0.0, 4.0 | 3 (0.3) [0.1, 0.8] |
| Injection site reaction | 0 [0.0, 2.4] | 3 (0.5) [0.1, 1.4] | 0 [0.0, 3.2] | 3 (0.3) [0.1, 1.0] | 0 [0.0, 2.7] | 3 (0.3) [0.1, 0.8] |
| Vaccination site reaction | 0 [0.0, 2.4] | 2 (0.3) [0.0, 1.1] | 1 ( 0.9) [0.0, 4.9] | 3 (0.3) [0.1, 1.0] | 0 [0.0, 2.7] | 3 (0.3) [0.1, 0.8] |
| Drug ineffective | 0 [0.0, 2.4] | 2 (0.3) [0.0, 1.1] | 0 [0.0, 3.2] | 2 (0.2) [0.0, 0.8] | 0 [0.0, 2.7] | 2 (0.2) [0.0 0.7] |
| Vaccination site erythema | 0 [0.0, 2.4] | 2 (0.3) [0.0, 1.1] | 0 [0.0, 3.2] | 2 (0.2) [0.0, 0.8] | 0 [0.0, 2.7] | 2 (1.0) [0.0, 0.7] |
| Adverse drug reaction | 0 [0.0, 2.4] | 1 (0.2) [0.0, 0.9] | 0 [0.0, 3.2] | 1 (0.1) [0.0, 0.6] | 0 [0.0, 2.7] | 1 (1.0) [0.0, 0.5] |
| Asthenia | 0 [0.0, 2.4] | 1 (0.2) [0.0, 0.9] | 0 [0.0, 3.2] | 1 (0.1) [0.0, 0.6] | 0 [0.0, 2.7] | 1 (0.1) [0.0, 0.5] |
| Chest pain | 0 [0.0, 2.4] | 1 (0.2) [0.0, 0.9] | 0 [0.0, 3.2] | 1 (0.1) [0.0, 0.6] | 0 [0.0, 2.7] | 1 (0.1) [0.0, 0.5] |
| Feeling cold | 0 [0.0, 2.4] | 1 (0.2) [0.0, 0.9] | 0 [0.0, 3.2] | 1 (0.1) [0.0, 0.6] | 0 [0.0, 2.7] | 1 (0.1) [0.0, 0.5] |
| Hunger | 0 [0.0, 2.4] | 1 (0.2) [0.0, 0.9] | 0 [0.0, 3.2] | 1 (0.1) [0.0, 0.6] | 0 [0.0, 2.7] | 1 (0.1) [0.0, 0.5] |
| Immediate post-injection reaction | 0 [0.0, 2.4] | 1 (0.2) [0.0, 0.9] | 0 (0) [0.0, 3.2] | 1 (0.1) [0.0, 0.6] | 0 (0) [0.0, 2.7] | 1 (0.1) [0.0, 0.5] |
| Impaired healing | 0 [0.0, 2.4] | 0 [0.0, 0.6] | 1 (0.9) [0.0, 4.9] | 1 (0.1) [0.0, 0.6] | 0 (0) [0.0, 2.7] | 1 (0.1) [0.0, 0.5] |
| Injection site erythema | 0 [0.0, 2.4] | 1 (0.2) [0.0, 0.9] | 0 [0.0, 3.2] | 1 (0.1) [0.0, 0.6] | 0 [0.0, 2.7] | 1 (0.1) [0.0, 0.5] |
| Injection site hematoma | 0 [0.0, 2.4] | 1 (0.2) [0.0, 0.9] | 0 [0.0, 3.2] | 1 (0.1) [0.0, 0.6] | 0 [0.0, 2.7] | 1 (0.1) [0.0, 0.5] |
| Injection site irritation | 0 [0.0, 2.4] | 1 (0.2) [0.0, 0.9] | 0 [0.0, 3.2] | 1 (0.1) [0.0, 0.6] | 0 [0.0, 2.7] | 1 (0.1) [0.0, 0.5] |

| SOC, Preferred term, n (IR/ 100 PY) (95% CI) | FCAS N= 42 | MWS N=169 | NOMID N= 32 | All CAPS N=243 | Other* N= 42 | ALL N=285 |
|---|---|---|---|---|---|---|
| Localized edema | 0 [0.0, 2.4] | 0 [0.0, 0.6] | 0 [0.0, 3.2] | 0 [0.0, 0.4] | 1 (0.7) [0.0, 4.0] | 1 (0.1) [0.0, 0.5] |
| Nodule | 0 [0.0, 2.4] | 0 [0.0, 0.6] | 0 [0.0, 3.2] | 0 [0.0, 0.4] | 1 (0.7) [0.0, 4.0] | 1 (0.1) [0.0, 0.5] |
| Non-cardiac chest pain | 0 [0.0, 2.4] | 1 (0.2) [0.0, 0.9] | 0 [0.0, 3.2] | 1 (0.1) [0.0, 0.6] | 0 [0.0, 2.7] | 1 (0.1) [0.0, 0.5] |
| Peripheral swelling | 0 [0.0, 2.4] | 1 (0.2) [0.0, 0.9] | 0 [0.0, 3.2] | 1 (0.1) [0.0, 0.6] | 0 [0.0, 2.7] | 1 (0.1) [0.0, 0.5] |
| Therapeutic product ineffective | 0 [0.0, 2.4] | 1 (0.2) [0.0, 0.9] | 0 [0.0, 3.2] | 1 (0.1) [0.0, 0.6] | 0 [0.0, 2.7] | 1 (0.1) [0.0, 0.5] |
| **Gastrointestinal disorders** | 8 (5.1) [2.2, 10.1] | 51 (7.9) [5.9, 10.3] | 6 (5.3) [1.9, 11.5] | 65 (7.1) [5.5, 9.0] | 24 (17.2) [11.0, 25.6] | 89 (8.4) [6.8, 10.3] |
| Diarrhea | 1 (0.6) [0.0, 3.6] | 9 (1.4) [0.6, 2.6) | 3 (2.6) [0.5 7.7] | 13 (1.4) [0.8, 2.4] | 3 (2.1) [0.4, 6.3] | 16 (1.5) [0.9, 2.5] |
| Abdominal pain | 0 [0.0, 2.4] | 3 (0.5) [0.1, 1.4] | 3 (2.6) [0.5, 7.7] | 6 (0.7) [0.24, 1.4] | 5 (3.6) [1.2, 8.3] | 11 (1.0) [0.5, 1.9] |
| Abdominal pain upper | 0 [0.0, 2.4] | 6 ( 0.9) [0.3, 2.0] | 0 [0.0, 3.2] | 6 (0.7) [0.2, 1.4] | 1 (0.7) [0.0, 4.0] | 7 (0.7) [0.3, 1.4] |
| Vomiting | 0 [0.0, 2.4] | 3 (0.5) [0.1, 1.4] | 0 [0.0, 3.2] | 3 (0.3) [0.1, 1.0] | 4 (2.9) [0.8, 7.3] | 7 (0.7) [0.3, 1.4] |
| Constipation | 1 (0.6) [0.0, 3.6] | 4 (0.6) [0.2, 1.6] | 0 [0.0, 3.2] | 5 (0.5) [0.2, 1.3] | 1 (0.7) [0.0, 4.0] | 6 (0.6) [0.2, 1.2] |
| Pancreatitis | 0 [0.0, 2.4] | 6 (0.9) [0.3, 2.0] | 0 [0.0, 3.2] | 6 (0.7) [0.2, 1.4] | 0 [0.0, 2.7] | 6 (0.6) [0.2, 1.2] |
| Anal fissure | 0 [0.0, 2.4] | 2 (0.3) [0.0, 1.1] | 0 [0.0, 3.2] | 2 (0.2) [0.0, 0.8] | 2 (1.4) [0.2, 5.2] | 4 (0.4) [0.1, 1.0] |
| Aphthous ulcer | 2 (1.3) [0.2, 4.6] | 2 (0.3) [0.0, 1.1] | 0 [0.0, 3.2] | 4 (0.4) [0.1, 1.1] | 0 [0.0, 2.7] | 4 (0.4) [0.1, 1.0] |
| Gastroesophageal | 1 (0.6) [0.0, 3.4] | 3 (0.5) [0.1, 1.4] | 0 [0.0, 3.2] | 4 (0.4) [0.1, 1.1] | 0 [0.0, 2.7] | 4 (0.4) [0.1, 1.0] |

| SOC, Preferred term, n (IR/ 100 PY) (95% CI) | FCAS N= 42 | MWS N=169 | NOMID N= 32 | All CAPS N=243 | Other* N= 42 | ALL N=285 |
|---|---|---|---|---|---|---|
| reflux disease | | | | | | |
| Gastritis | 0 [0.0, 2.4] | 1 (0.2) [0.0, 0.9] | 0 [0.0, 3.2] | 1 (0.1) [0.0, 0.6] | 2 (1.4) [0.2, 5.2] | 3 (0.3) [0.1, 1.0] |
| Dental necrosis | 0 [0.0, 2.4] | 2 (0.3) [0.0, 1.1] | 0 [0.0, 3.2] | 2 (0.2) [0.0, 0.8] | 0 [0.0, 2.7] | 2 (0.2) [0.0, 0.7] |
| Gingival pain | 1 (0.6) [0.0, 3.6] | 1 (0.2) [0.0, 0.9] | 0 [0.0, 3.2] | 2 (0.2) [0.0, 0.8] | 0 [0.0, 2.7] | 2 (0.2) [0.0, 0.7] |
| Mouth ulceration | 0 [0.0, 2.4] | 1 (0.2) [0.0, 0.9] | 0 [0.0, 3.2] | 1 (0.1) [0.0, 0.6] | 1 (0.7) [0.0, 4.0] | 2 (0.2) [0.0, 0.7] |
| Abdominal hernia | 1 (0.6) [0.0, 3.6] | 0 [0.0, 0.6] | 0 [0.0, 3.2] | 1 (0.1) [0.0, 0.6] | 0 [0.0, 2.7] | 1 (0.1) [0.0, 0.5] |
| Abdominal pain lower | 0 [0.0, 2.4] | 0 [0.0, 0.6] | 0 [0.0, 3.2] | 0 [0.0, 0.4] | 1 (0.7) [0.0, 4.0] | 1 (0.1) [0.0, 0.5] |
| Dental caries | 1 (0.6) [0.0, 3.6] | 0 [0.0, 0.6] | 0 [0.0, 3.2] | 1 (0.1) [0.0, 0.6] | 0 [0.0, 2.7] | 1 (0.1) [0.0, 0.5] |
| Dyspepsia | 0 [0.0, 2.4] | 1 (0.2) [0.0, 0.9] | 0 [0.0, 3.2] | 1 (0.1) [0.0, 0.6] | 0 [0.0, 2.7] | 1 (0.1) [0.0, 0.5] |
| Enterocolitis | 0 [0.0, 2.4] | 0 [0.0, 0.6] | 0 [0.0, 3.2] | 0 [0.0, 0.4] | 1 (0.7) [0.0, 4.0] | 1 (0.1) [0.0, 0.5] |
| Flatulence | 0 [0.0, 2.4] | 1 (0.2) [0.0, 0.9] | 0 [0.0, 3.2] | 1 [0.0, 0.6] | 0 [0.0, 2.7] | 1 (0.1) [0.0, 0.5] |
| Gastrointestinal toxicity | 0 [0.0, 2.4] | 0 (0) [0.0, 0.6] | 0 [0.0, 3.2] | 0 [0.0, 0.4] | 1 (0.7) [0.0, 4.0] | 1 (0.1) [0.0, 0.5] |
| Haemorrhoids | 0 [0.0, 2.4] | 1 (0.2) [0.0, 0.9] | 0 [0.0, 3.2] | 1 (0.1) [0.0, 0.6] | 0 [0.0, 2.7] | 1 (0.1) [0.0, 0.5] |
| Inguinal hernia | 0 [0.0, 2.4] | 0 [0.0, 0.6] | 0 [0.0, 3.2] | 0 [0.0, 0.4] | 1 (0.7) [0.0, 4.0] | 1 (0.1) [0.0, 0.5] |
| Large intestine polyp | 0 [0.0, 2.4] | 1 (0.2) [0.0, 0.9] | 0 [0.0, 3.2] | 1 (0.1) [0.0, 0.6] | 0 [0.0, 2.7] | 1 (0.1) [0.0, 0.5] |
| Lip blister | 0 [0.0, 2.4] | 1 (0.2) [0.0, 0.9] | 0 [0.0, 3.2] | 1 (0.1) [0.0, 0.6] | 0 [0.0, 2.7] | 1 (0.1) [0.0, 0.5] |

| SOC, Preferred term, n (IR/ 100 PY) (95% CI) | FCAS N= 42 | MWS N=169 | NOMID N= 32 | All CAPS N=243 | Other* N= 42 | ALL N=285 |
|---|---|---|---|---|---|---|
| Nausea | 0 [0.0, 2.4] | 1 (0.2) [0.0, 0.9] | 0 [0.0, 3.2] | 1 (0.1) [0.0, 0.6] | 0 [0.0, 2.7] | 1 (0.1) [0.0, 0.5] |
| Noninfective gingivitis | 0 [0.0, 2.4] | 1 (0.2) [0.0, 0.9] | 0 [0.0, 3.2] | 1 (0.1) [0.0, 0.6] | 0 [0.0, 2.7] | 1 (0.1) [0.0, 0.5] |
| Odynophagia | 0 [0.0, 2.4] | 0 (0) [0.0, 0.6] | 0 [0.0, 3.2] | 0 [0.0, 0.4] | 1 (0.7) [0.0, 4.0] | 1 (0.1) [0.0, 0.5] |
| Umbilical hernia | 0 [0.0, 2.4] | 1 (0.2) [0.0, 0.9] | 0 [0.0, 3.2] | 1 (0.1) [0.0, 0.6] | 0 [0.0, 2.7] | 1 (0.1) [0.0, 0.5] |
| **Musculoskeletal and connective tissue disorders** | 8 (5.1) [2.2, 10.1] | 35 (5.4) [3.8, 7.5] | 4 (3.5) [1.0, 9.0] | 47 (5.1) [3.8, 6.8] | 21 (15.0) [9.3, 23.0] | 68 (6.4) [5.0, 8.2] |
| Arthralgia | 2(1.3) [0.2, 4.6] | 6 (0.9) [0.3, 2.0] | 0 [0.0, 3.2] | 8 (0.9) [0.4, 1.7] | 3 (2.1) [0.4, 6.3] | 11 (1.0) [0.5, 1.9] |
| Back pain | 1 (0.6) [0.0, 3.6] | 5 (0.8) [0.3, 1.8] | 0 [0.0, 3.2] | 6 (0.7) [0.2, 1.4] | 2 (1.4) [0.2, 5.2] | 8 (0.8) [0.3, 1.5] |
| Pain in extremity | 1(0.6) [0.0, 3.6] | 3 (0.5) [0.1, 1.4] | 0 [0.0, 3.2] | 4 (0.4) [0.1, 1.2] | 3 (2.2) [0.4, 6.3] | 7 (0.7) [0.3, 1.4] |
| Synovitis | 0 [0.0, 2.4] | 4 (0.6) [0.2, 1.6] | 0 [0.0, 3.2] | 4 (0.4) [0.1, 1.1] | 0 [0.0, 2.7] | 4 (0.4) [0.1, 1.0] |
| Muscle spasms | 0 [0.0, 2.4] | 3 (0.5) [0.1, 1.4] | 0 [0.0, 3.2] | 3 (0.3) [0.1, 1.0] | 0 [0.0, 2.7] | 3 (0.3) [0.1, 0.8] |
| Musculoskeletal pain | 1 (0.6) [0.0, 3.6] | 1(0.2) [0.0, 0.9] | 0 [0.0, 3.2] | 2 (0.2) [0.0, 0.8] | 1 (0.7) [0.0, 4.0] | 3 (0.3) [0.1, 0.8] |
| Neck pain | 1 (0.6) [0.0, 3.6] | 1(0.2) [0.0, 0.9] | 0 [0.0, 3.2] | 2 (0.2) [0.0, 0.8] | 1 (0.7) [0.0, 4.0] | 3 (0.3) [0.1, 0.8] |
| Coccydynia | 1 (0.6) [0.0, 3.6] | 0 [0.0, 0.6] | 0 [0.0, 3.2] | 1 (0.1) [0.0, 0.6] | 1 (0.7) [0.0, 4.0] | 2 (0.2) [0.0, 0.7] |
| Knee deformity | 0 [0.0, 2.4] | 0 [0.0, 0.6] | 2 (1.8) [0.2, 6.3] | 2 (0.2) [0.0, 0.8] | 0 [0.0, 2.7] | 2 (0.2) [0.0, 0.7] |
| Myalgia | 0 [0.0, 2.4] | 1 (0.2) [0.0, 0.9] | 0 [0.0, 3.2] | 1 (0.2) [0.0, 0.6] | 1 (0.7) [0.0, 4.0] | 2 (0.2) [0.0, 0.7] |

| SOC, Preferred term, n (IR/ 100 PY) (95% CI) | FCAS N= 42 | MWS N=169 | NOMID N= 32 | All CAPS N=243 | Other* N= 42 | ALL N=285 |
|---|---|---|---|---|---|---|
| Plantar fasciitis | 0 [0.0, 2.4] | 2 (0.3) [0.0, 1.1] | 0 [0.0, 3.2] | 2 (0.2) [0.0, 0.8] | 0 [0.0, 2.7] | 2 (0.2) [0.0, 0.7] |
| Synovial cyst | 0 [0.0, 2.4] | 1 (0.2) [0.0, 0.9] | 0 [0.0, 3.2] | 1 (0.1) [0.0, 0.6] | 1 (0.7) [0.0, 4.0] | 2 (0.2) [0.0, 0.7] |
| Tendonitis | 1 (0.6) [0.0, 3.6] | 0 [0.0, 0.6] | 0 [0.0, 3.2] | 1 (0.1) [0.0, 0.6] | 1 (0.7) [0.0, 4.0] | 2 (0.2) [0.0, 0.7] |
| Acquired claw toe | 0 [0.0, 2.4] | 0 [0.0, 0.6] | 0 [0.0, 3.2] | 0 [0.0, 0.4] | 1 (0.7) [0.0, 4.0] | 1 (0.1) [0.0, 0.5] |
| Arthropathy | 0 [0.0, 2.4] | 0 [0.0, 0.6] | 0 [0.0, 3.2] | 0 [0.0, 0.4] | 1 (0.7) [0.0, 4.0] | 1 (0.1) [0.0, 0.5] |
| Bursitis | 0 [0.0, 2.4] | 0 [0.0, 0.6] | 0 [0.0, 3.2] | 0 [0.0, 0.4] | 1 (0.7) [0.0, 4.0] | 1 (0.1) [0.0, 0.5] |
| Chondropathy | 0 [0.0, 2.4] | 1 (0.2) [0.0, 0.9] | 0 (0) [0.0, 3.2] | 1 (0.1) [0.0, 0.6] | 0 [0.0, 2.7] | 1 (0.1) [0.0, 0.5] |
| Costochondritis | 0 [0.0, 2.4] | 1 (0.2) [0.0, 0.9] | 0 (0) [0.0, 3.2] | 1 (0.1) [0.0, 0.6] | 0 [0.0, 2.7] | 1 (0.1) [0.0, 0.5] |
| Fibromyalgia | 0 [0.0, 2.4] | 1 (0.2) [0.0, 0.9] | 0 (0) [0.0, 3.2] | 1 (0.1) [0.0, 0.6] | 0 [0.0, 2.7] | 1 (0.1) [0.0, 0.5] |
| Interventional disc protrusion | 0 [0.0, 2.4] | 1 (0.2) [0.0, 0.9] | 0 [0.0, 3.2] | 1 (0.1) [0.0, 0.6] | 0 [0.0, 2.7] | 1 (0.1) [0.0, 0.5] |
| Joint swelling | 0 [0.0, 2.4] | 0 [0.0, 0.6] | 1 (0.9) [0.0, 4.9] | 1 (0.1) [0.0, 0.6] | 0 [0.0, 2.7] | 1 (0.1) [0.0, 0.5] |
| Joint warmth | 0 [0.0, 2.4] | 0 [0.0, 0.6] | 1 (0.9) [0.0, 4.9] | 1 (0.1) [0.0, 0.6] | 0 [0.0, 2.7] | 1 (0.1) [0.0, 0.5] |
| Limb discomfort | 0 [0.0, 2.4] | 0 [0.0, 0.6] | 0 [0.0, 3.2] | 0 [0.0, 0.4] | 1 (0.7) [0.0, 4.0] | 1 (0.1) [0.0, 0.5] |
| Lumbar spinal stenosis | 0 [0.0, 2.4] | 1 (0.2) [0.0, 0.9] | 0 [0.0, 3.2] | 1 (0.1) [0.0, 0.6] | 0 [0.0, 2.7] | 1 (0.1) [0.0, 0.5] |
| Musculoskeletal chest pain | 0 [0.0, 2.4] | 1 (0.2) [0.0, 0.9] | 0 [0.0, 3.2] | 1 (0.1) [0.0, 0.6] | 0 [0.0, 2.7] | 1 (0.1) [0.0, 0.5] |

| SOC, Preferred term, n (IR/ 100 PY) (95% CI) | FCAS N= 42 | MWS N=169 | NOMID N= 32 | All CAPS N=243 | Other* N= 42 | ALL N=285 |
|---|---|---|---|---|---|---|
| Musculoskeletal stiffness | 0 [0.0, 2.4] | 0 [0.0, 0.6] | 0 [0.0, 3.2] | 0 [0.0, 0.4] | 1 (0.7) [0.0, 4.0] | 1 (0.1) [0.0, 0.5] |
| Osteoporosis | 0 [0.0, 2.4] | 0 [0.0, 0.6] | 0 [0.0, 3.2] | 0 [0.0, 0.4] | 1 (0.7) [0.0, 4.0] | 1 (0.1) [0.0, 0.5] |
| Sacroiliitis | 0 [0.0, 2.4] | 1 (0.2) [0.0, 0.9] | 0 [0.0, 3.2] | 1 (0.1) [0.0, 0.6] | 0 [0.0, 2.7] | 1 (0.1) [0.0, 0.5] |
| Scoliosis | 0 [0.0, 2.4] | 0 [0.0, 0.6] | 0 [0.0, 3.2] | 0 [0.0, 0.4] | 1 (0.7) [0.0, 4.0] | 1 (0.1) [0.0, 0.5] |
| Tendon pain | 0 [0.0, 2.4] | 1 (0.2) [0.0, 0.9] | 0 [0.0, 3.2] | 1 (0.1) [0.0, 0.6] | 0 [0.0, 2.7] | 1 (0.1) [0.0, 0.5] |
| **Nervous system disorders** | 5 (3.2) [1.0, 7.5] | 33 (5.1) [3.5, 7.1] | 17 (15.0) [8.7, 23.9] | 55 (6.0) [4.5, 7.8] | 11 (7.9) [3.9, 14.1] | 66 (6.2) [4.8, 7.9] |
| Headache | 2 (1.3) [0.2, 4.6] | 12 (1.9) [1.0, 3.2] | 10 ( 8.8) [4.2,16.2] | 24 (2.6) [1.7, 3.9] | 7 (5.0) [2.0,10.3] | 31 (2.9) [2.0, 4.2] |
| Dizziness | 1(0.6) [0.0, 3.6] | 4 (0.6) [0.2, 1.6] | 1 ( 0.9) [0.0, 4.9] | 6 (0.7) [0.2, 1.4] | 1 (0.7) [0.0, 4.0] | 7 (0.7) [0.3, 1.4] |
| Syncope | 0 [0.0, 2.4] | 3 (0.5) [0.1, 1.4] | 2 (1.8) [0.2, 6.4] | 5 (0.5) [0.2, 1.3] | 0 [0.0, 2.7] | 5 (0.5) [0.2, 1.1] |
| Intracranial pressure increased | 0 [0.0, 2.4] | 0 [0.0, 0.6] | 2 (1.8) [0.2, 6.4] | 2 (0.2) [0.0, 0.8] | 1 (0.7) [0.0, 4.0] | 3 (0.3) [0.1, 0.8] |
| Paraesthesia | 0 [0.0, 2.4] | 3 (0.5) [0.1, 1.4] | 0 [0.0, 3.2] | 3 (0.3) [0.1, 1.0] | 0 [0.0, 2.7] | 3 (0.3) [0.1, 0.8] |
| Migraine | 1 (0.6) [0.0, 3.6] | 1 (0.2) [0.0, 0.9] | 0 [0.0, 3.2] | 2 (0.2) [0.0, 0.8] | 0 [0.0, 2.7] | 2 (0.2) [0.0, 0.7] |
| Sciatica | 0 [0.0, 2.4] | 2 (0.3) [0.0, 1.1] | 0 [0.0, 3.2] | 2 (0.2) [0.0, 0.8] | 0 [0.0, 2.7] | 2 (0.2) [0.0, 0.7] |
| Amnesia | 0 [0.0, 2.4] | 0 [0.0, 0.6] | 0 [0.0, 3.2] | 0 [0.0, 0.4] | 1 (0.7) [0.0, 4.0] | 1 (0.1) [0.0, 0.5] |
| Brain mass | 0 [0.0, 2.4] | 1 (0.2) [0.0, 0.9] | 0 [0.0, 3.2] | 1 (0.1) [0.0, 0.6] | 0 [0.0, 2.7] | 1 (0.1) [0.0, 0.5] |

| SOC, Preferred term, n (IR/ 100 PY) (95% CI) | FCAS N= 42 | MWS N=169 | NOMID N= 32 | All CAPS N=243 | Other* N= 42 | ALL N=285 |
|---|---|---|---|---|---|---|
| Carpal tunnel syndrome | 0 [0.0, 2.4] | 0 [0.0, 0.6] | 1 (0.8) [0.0, 4.9] | 1 (0.1) [0.0, 0.6] | 0 [0.0, 2.7] | 1 (0.1) [0.0, 0.5] |
| Cerebral infarction | 1 (0.6) [0.0, 3.6] | 0 [0.0, 0.6] | 0 [0.0, 3.2] | 1 (0.1) [0.0, 0.6] | 0 [0.0, 2.7] | 1 (0.1) [0.0, 0.5] |
| Demyelination | 0 [0.0, 2.4] | 1 (0.2) [0.0, 0.9] | 0 [0.0, 3.2] | 1 (0.1) [0.0, 0.6] | 0 [0.0, 2.7] | 1 (0.1) [0.0, 0.5] |
| Epilepsy | 0 [0.0, 2.4] | 0 [0.0, 0.6] | 1 (0.9) [0.0, 4.9] | 1 (0.1) [0.0, 0.6] | 0 [0.0, 2.7] | 1 (0.1) [0.0, 0.5] |
| Hypoaesthesia | 0 [0.0, 2.4] | 1 (0.2) [0.0, 0.9] | 0 [0.0, 3.2] | 1 (0.1) [0.0, 0.6] | 0 [0.0, 2.7] | 1 (0.1) [0.0, 0.5] |
| Nervous system disorder | 0 [0.0, 2.4] | 1 (0.2) [0.0, 0.9] | 0 [0.0, 3.2] | 1 (0.1) [0.0, 0.6] | 0 [0.0, 2.7] | 1 (0.1) [0.0, 0.53] |
| Psychomotor hyperactivity | 0 [0.0, 2.4] | 1 (0.2) [0.0, 0.9] | 0 [0.0, 3.2] | 1 (0.1) [0.0, 0.6] | 0 [0.0, 2.7] | 1 (0.1) [0.0, 0.5] |
| Seizure | 0 [0.0, 2.4] | 1 (0.2) [0.0, 0.9] | 0 [0.0, 3.2] | 1 (0.1) [0.0, 0.6] | 0 [0.0, 2.7] | 1 (0.1) [0.0, 0.5] |
| Sensory loss | 0 [0.0, 2.4] | 1 (0.2) [0.0, 0.9] | 0 [0.0, 3.2] | 1 (0.1) [0.0, 0.6] | 0 [0.0, 2.7] | 1 (0.1) [0.0, 0.5] |
| Facial nerve paralysis | 0 [0.0, 2.4] | 1 (0.2) [0.0, 0.9] | 0 [0.0, 3.2] | 1 (0.1) [0.0, 0.6] | 0 [0.0, 2.7] | 1 (0.1) [0.0, 0.5] |
| Visual field defect | 0 [0.0, 2.4] | 0 [0.0, 0.6] | 0 [0.0, 3.2] | 0 [0.0, 0.4] | 1 (0.7) [0.0, 4.0] | 1 (0.1) [0.0, 0.5] |
| **Respiratory, thoracic and mediastinal disorders** | 3 (1.9) [0.4, 5.6] | 32 (4.9) [3.4, 7.0] | 2 (1.8) [0.2, 6.3] | 37 (4.0) [2.8, 5.6] | 17 (12.2) [7.1, 19.5] | 54 (5.1) [3.8, 6.7] |
| Cough | 1 (0.6) [0.0, 3.6] | 7 (1.1) [0.4, 2.2] | 1 ( 0.9) [0.0, 4.9] | 8 (0.9) [0.5, 1.9] | 7 (5.0) [2.0, 10.3] | 16 (1.5) [0.9, 2.5] |
| Oropharyngeal pain | 2 (1.3) [0.2, 4.6] | 5 (0.8) [0.3, 1.8] | 1 ( 0.9) [0.0, 4.9] | 8 (0.9) [0.4, 1.7] | 4 (2.9) [0.8, 7.3] | 12 (1.1) [0.6, 2.0] |

| SOC, Preferred term, n (IR/ 100 PY) (95% CI) | FCAS N= 42 | MWS N=169 | NOMID N= 32 | All CAPS N=243 | Other* N= 42 | ALL N=285 |
|---|---|---|---|---|---|---|
| Dyspnea | 0 [0.0, 2.4] | 4 (0.6) [0.2, 1.6] | 0 [0.0, 3.2] | 4 (0.4) [0.1, 1.1] | 2 (1.4) [0.2, 5.2] | 6 (0.6) [0.2, 1.2] |
| Asthma | 0 [0.0, 2.4] | 4 (0.6) [0.2, 1.6] | 0 [0.0, 3.2] | 4 (0.4) [0.1, 1.1] | 1 (0.7) [0.0, 4.0] | 5 (0.5) [0.2, 1.1] |
| Rhinitis allergic | 0 [0.0, 2.4] | 3 (0.5) [0.1, 1.4] | 0 [0.0, 3.2] | 3 (0.3) [0.1, 1.0] | 0 [0.0, 2.7] | 3 (0.3) [0.1, 0.9] |
| Sleep apnoea syndrome | 0 [0.0, 2.4] | 2 (0.3) [0.0, 1.1] | 0 [0.0, 3.2] | 2 (0.2) [0.0, 0.8] | 0 [0.0, 2.7] | 2 (0.2) [0.0, 0.7] |
| Acute respiratory distress syndrome | 0 [0.0, 2.4] | 0 [0.0, 0.6] | 0 [0.0, 3.2] | 0 [0.0, 0.4] | 1 (0.7) [0.0, 4.0] | 1 (0.1) [0.0, 0.5] |
| Adenoidal hypertrophy | 0 [0.0, 2.4] | 1 (0.2) [0.0, 0.9] | 0 [0.0, 3.2] | 1 (0.1) [0.0, 0.6] | 0 [0.0, 2.7] | 1 (0.1) [0.0, 0.5] |
| Bronchial disorder | 0 [0.0, 2.4] | 1 (0.2) [0.0, 0.9] | 0 [0.0, 3.2] | 1 (0.1) [0.0, 0.6) | 0 [0.0, 2.7] | 1 (0.1) [0.0, 0.5] |
| Bronchitis chronic | 0 [0.0, 2.4] | 1 (0.2) [0.0, 0.9] | 0 [0.0, 3.2] | 1 (0.1) [0.0, 0.6] | 0 [0.0, 2.7] | 1 (0.1) [0.0, 0.5] |
| Dysphonia | 0 [0.0, 2.4] | 1 (0.2) [0.0, 0.9] | 0 [0.0, 3.2] | 1 (0.1) [0.0, 0.6] | 0 [0.0, 2.7] | 1 (0.1) [0.0, 0.5] |
| Nasal discharge discolouration | 0 [0.0, 2.4] | 0 [0.0, 0.6] | 0 [0.0, 3.2] | 0 [0.0, 0.4] | 1 (0.7) [0.0, 4.0] | 1 (0.1) [0.0, 0.5] |
| Nasal turbinate hypertrophy | 0 [0.0, 2.4] | 1 (0.2) [0.0, 0.9] | 0 [0.0, 3.2] | 1 (0.1) [0.0, 0.6] | 0 [0.0, 2.7] | 1 (0.1) [0.0, 0.5] |
| Pleural effusion | 0 [0.0, 2.4] | 1 (0.2) [0.0, 0.9] | 0 [0.0, 3.2] | 1 (0.1) [0.0, 0.6] | 0 [0.0, 2.7] | 1 (0.1) [0.0, 0.5] |
| Rhinorrhoea | 0 [0.0, 2.4] | 0 [0.0, 0.6] | 0 [0.0, 3.2] | 0 [0.0, 0.4] | 1 (0.7) [0.0, 4.0] | 1 (0.1) [0.0, 0.5] |
| Sinus congestion | 0 [0.0, 2.4] | 1 (0.2) [0.0, 0.9] | 0 [0.0, 3.2] | 1 (0.1) [0.0, 0.6] | 0 [0.0, 2.7] | 1 (0.1) [0.0, 0.5] |
| **Investigations** | 9 (5.8) [2.6, 11.0] | 30 (4.6) [3.1, 6.6] | 7 (6.2) [2.5, 12.7] | 46 (5.0) [3.7, 6.7] | 1 (0.7) [0.0, 4.0] | 47 (4.4) [3.3, 5.9] |

| SOC, Preferred term, n (IR/ 100 PY) (95% CI) | FCAS N= 42 | MWS N=169 | NOMID N= 32 | All CAPS N=243 | Other* N= 42 | ALL N=285 |
|---|---|---|---|---|---|---|
| Protein urine present | 4 (2.6) [0.7, 6.6] | 3 (0.5) [0.1, 1.4] | 5 (4.4) [1.4,10.3] | 12 (1.3) [0.7, 2.3] | 0 [0.0, 2.7] | 12 (1.1) [0.6, 2.0] |
| Weight gain | 1(0.6) [0.0, 3.6] | 9 (1.4) [0.6, 2.6] | 0 [0.0, 3.2] | 10 (1.1) [0.5, 2.0] | 0 [0.0, 2.7] | 10 (0.9) [0.5, 1.7] |
| Antinuclear antibody positive | 0 [0.0, 2.4] | 4 (0.6) [0.2, 1.6] | 0 [0.0, 3.2] | 4 (0.4) [0.1, 1.1] | 0 [0.0, 2.7] | 4 (0.4) [0.1, 1.0] |
| Hypercholesterolemia | 3 (1.9) [0.4, 5.6] | 0 [0.0, 0.6] | 1 ( 0.9) [0.0, 4.9] | 4 (0.4) [0.1, 1.1] | 0 [0.0, 2.7] | 4 (0.4) [0.1, 1.0] |
| Liver enzymes elevated | 1 (0.6) [0.0, 3.6] | 3 (0.5) [0.1, 1.4] | 0 [0.0, 3.2] | 4 (0.4) [0.1, 1.1] | 0 [0.0, 2.7] | 4 (0.4) [0.1, 1.0] |
| Gamma-glutamyl transferase increased | 0 [0.0, 2.4] | 3 (0.5) [0.1, 1.4] | 0 [0.0, 3.2] | 3 (0.3) [0.1, 1.0] | 0 [0.0, 2.7] | 3 (0.3) [0.1, 0.9] |
| Low density lipoprotein increased | 0 [0.0, 2.4] | 1 (0.2) [0.0, 0.9] | 1 (0.9) [0.0, 4.9] | 2 (0.2) [0.0, 0.8] | 0 [0.0, 2.7] | 2 (0.2) [0.0, 0.7] |
| Alanine aminotransferase increased | 0 [0.0, 2.4] | 1 (0.2) [0.0, 0.9] | 0 [0.0, 3.2] | 1 (0.1) [0.0, 0.6] | 0 [0.0, 2.7] | 1 (0.1) [0.0, 0.5] |
| Aspartate aminotransferase increased | 0 [0.0, 2.4] | 1 (0.2) [0.0, 0.9] | 0 [0.0, 3.2] | 1 (0.1) [0.0, 0.6] | 0 [0.0, 2.7] | 1 (0.1) [0.0, 0.5] |
| Blood creatinine phosphokinase increased | 0 [0.0, 2.4] | 1 (0.2) [0.0, 0.9] | 0 [0.0, 3.2] | 1 (0.1) [0.0, 0.6] | 0 [0.0, 2.7] | 1 (0.1) [0.0, 0.5] |
| Blood glucose increased | 0 [0.0, 2.4] | 1 (0.2) [0.0, 0.9] | 0 [0.0, 3.2] | 1 (0.1) [0.0, 0.6] | 0 [0.0, 2.7] | 1 (0.1) [0.0, 0.5] |

| SOC, Preferred term, n (IR/ 100 PY) (95% CI) | FCAS N= 42 | MWS N=169 | NOMID N= 32 | All CAPS N=243 | Other* N= 42 | ALL N=285 |
|---|---|---|---|---|---|---|
| Creatinine renal clearance decreased | 0 [0.0, 2.4] | 1 (0.2) [0.0, 0.9] | 0 [0.0, 3.2] | 1 (0.1) [0.0, 0.6] | 0 [0.0, 2.7] | 1 (0.1) [0.0, 0.5] |
| Haemoglobin decreased | 0 [0.0, 2.4] | 1 (0.2) [0.0, 0.9] | 0 (0) [0.0, 3.2] | 1 (0.1) [0.0, 0.6] | 0 (0) [0.0, 2.7] | 1 (0.1) [0.0, 0.5] |
| Serum amyloid A protein increased | 0 [0.0, 2.4] | 0 [0.0, 0.6] | 0 [0.0, 3.2] | 0 [0.0, 0.4] | 1 (0.7) [0.0, 4.0] | 1 (0.1) [0.0, 0.5] |
| Urine protein/creatinine ratio increased | 0 [0.0, 2.4] | 1 (0.2) [0.0, 0.9] | 0 [0.0, 3.2] | 1 (0.1) [0.0, 0.6] | 0 [0.0, 2.7] | 1 (0.1) [0.0, 0.5] |
| **Ear and labyrinth disorders** | 2 (1.3) [0.2, 4.6] | 28 (4.3) [2.9, 6.2] | 12 (10.5) [5.5, 18.4] | 42 (4.6) [3.3, 6.2] | 4 (2.9) [0.8, 7.3] | 46 (4.3)[3.2, 5.8] |
| Vertigo | 1 (0.6) [0.0, 3.6] | 21 (3.2) [2.0, 5.0] | 9 ( 7.9) [3.6,15.0] | 31 (3.4) [2.3, 4.8] | 3 (2.2) [0.4, 6.3] | 34 (3.2)[2.2, 4.5] |
| Ear pain | 0 [0.0, 2.4] | 2 (0.3) [0.0, 1.1] | 0 [0.0, 3.2] | 2 (0.) [0.0, 0.8] | 0 [0.0, 2.7] | 2 (0.2) [0.0, 0.7] |
| Tinnitus | 1 (0.6) [0.0, 3.6] | 1 (0.2) [0.0, 0.9] | 0 [0.0, 3.2] | 2 (0.2) [0.0, 0.8] | 0 [0.0, 2.7] | 2 (0.2) [0.0, 0.7] |
| Tympanic membrane perforation | 0 [0.0, 2.4] | 1 (0.2) [0.0, 0.9] | 1 (0.9) [0.0, 4.9] | 2 (0.2) [0.0, 0.8] | 0 [0.0, 2.7] | 2 (0.2) [0.0, 0.7] |
| Deafness | 0 [0.0, 2.4] | 0 [0.0, 0.6] | 1 (0.9) [0.0, 4.9] | 1 (0.1) [0.0, 0.6] | 0 [0.0, 2.7] | 1 (0.1) [0.0, 0.5] |
| Deafness unilateral | 0 [0.0, 2.4] | 0 [0.0, 0.6] | 0 [0.0, 3.2] | 0 [0.0, 0.4] | 1 (0.7) [0.0, 4.0] | 1 (0.1) [0.0, 0.5] |
| Eustachian tube disorder | 0 [0.0, 2.4] | 1 (0.2) [0.0, 0.9] | 0 [0.0, 3.2] | 1 (0.1) [0.0, 0.6] | 0 (0) [0.0, 2.7] | 1 (0.1) [0.0, 0.5] |
| External ear inflammation | 0 [0.0, 2.4] | 1 (0.2) [0.0, 0.9] | 0 [0.0, 3.2] | 1 (0.1) [0.0, 0.6] | 0 (0) [0.0, 2.7] | 1 (0.1) [0.0, 0.5] |

| SOC, Preferred term, n (IR/ 100 PY) (95% CI) | FCAS N= 42 | MWS N=169 | NOMID N= 32 | All CAPS N=243 | Other* N= 42 | ALL N=285 |
|---|---|---|---|---|---|---|
| Hearing impaired | 0 [0.0, 2.4] | 0 [0.0, 0.6] | 1 (0.9) [0.0, 4.9] | 1 (0.1) [0.0, 0.6] | 0 [0.0, 2.7] | 1 (0.1) [0.0, 0.5] |
| Sudden hearing loss | 0 [0.0, 2.4] | 1 (0.2) [0.0, 0.9] | 0 [0.0, 3.2] | 1 (0.1) [0.0, 0.6] | 0 [0.0, 2.7] | 1 (0.1) [0.0, 0.5] |
| **Skin and subcutaneous tissue damage** | 6 (3.9) [1.4, 8.4] | 28 (4.3) [2.9, 6.2] | 8 (7.0) [3.0, 13.9] | 42 (4.6) [3.3, 6.2] | 4 (2.9) [0.8 7.3] | 46 (4.4) [3.2, 5.8] |
| Rash | 0 [0.0, 2.4] | 8 (1.2) [0.5, 2.4] | 1 ( 0.9) [0.0, 4.9] | 9 (1.0) [0.4, 1.9] | 2 (1.4) [0.2, 5.2] | 11 (1.0) [0.5, 1.9] |
| Urticaria | 2 (1.3) [0.2, 4.6] | 2 (0.3) [0.0, 1.1] | 1 (0.9) [0.0, 4.9] | 5 (0.5) [0.2, 1.3] | 0 [0.0, 2.7] | 5 (0.5) [0.2, 1.1] |
| Eczema | 0 [0.0, 2.4] | 3 (0.5) [0.1, 1.4] | 1 (0.9) [0.0, 4.9] | 4 (0.4) [0.1, 1.1] | 0 [0.0, 2.7] | 4 (0.4) [0.1, 1.0] |
| Dry skin | 0 [0.0, 2.4] | 1 (0.2) [0.0, 0.9] | 1 ( 0.9) [0.0, 4.9] | 2 (0.2) [0.0, 0.8] | 0 [0.0, 2.7] | 2 (0.2) [0.0, 0.7] |
| Erythema | 0 [0.0, 2.4] | 1 (0.2) [0.0, 0.9] | 1 ( 0.9) [0.0, 4.9] | 2 (0.2) [0.0, 0.8] | 0 [0.0, 2.7] | 2 (0.2) [0.0, 0.7] |
| Perivascular dermatitis | 0 [0.0, 2.4] | 2 (0.3) [0.0, 1.1] | 0 [0.0, 3.2] | 2 (0.2) [0.0, 0.8] | 0 [0.0, 2.7] | 2 (0.2) [0.0, 0.7] |
| Pruritus generalised | 0 [0.0, 2.4] | 1 (0.2) [0.0, 0.9] | 0 [0.0, 3.2] | 1 (0.1) [0.0, 0.6] | 1 (0.7) [0.0, 4.0] | 2 (0.2) [0.0, 0.7] |
| Rash erythematous | 0 [0.0, 2.4] | 0 [0.0, 0.6] | 2 (1.8) [0.2, 6.4] | 2 (0.2) [0.0, 0.8] | 0 [0.0, 2.7] | 2 (0.2) [0.0, 0.7] |
| Acne | 0 [0.0, 2.4] | 1 (0.2) [0.0, 0.9] | 0 [0.0, 3.2] | 1 (0.1) [0.0, 0.6] | 0 [0.0, 2.7] | 1 (0.1) [0.0, 0.5] |
| Alopecia | 0 [0.0, 2.4] | 1 (0.2) [0.0, 0.9] | 0 [0.0, 3.2] | 1 (0.1) [0.0, 0.6] | 0 [0.0, 2.7] | 1 (0.1) [0.0, 0.5] |
| Dermatitis atopic | 0 [0.0, 2.4] | 0 [0.0, 0.6] | 0 [0.0, 3.2] | 0 [0.0, 0.4] | 1 (0.7) [0.0, 4.0] | 1 (0.1) [0.0, 0.5] |
| Hyperhidrosis | 0 [0.0, 2.4] | 1 (0.2) [0.0, 0.9] | 0 [0.0, 3.2] | 1 (0.1) [0.0, 0.6] | 0 [0.0, 2.7] | 1 (0.1) [0.0, 0.5] |

| SOC, Preferred term, n (IR/ 100 PY) (95% CI) | FCAS N= 42 | MWS N=169 | NOMID N= 32 | All CAPS N=243 | Other* N= 42 | ALL N=285 |
|---|---|---|---|---|---|---|
| Night sweats | 0 [0.0, 2.4] | 1 (0.2) [0.0, 0.9] | 0 [0.0, 3.2] | 1 (0.1) [0.0, 0.6] | 0 [0.0, 2.7] | 1 (0.1) [0.0, 0.5] |
| Pigmentation disorder | 1 (0.6) [0.0, 3.6] | 0 [0.0, 0.6] | 0 [0.0, 3.2] | 1 (0.1) [0.0, 0.6] | 0 [0.0, 2.7] | 1 (0.1) [0.0, 0.5] |
| Pruritus | 0 [0.0, 2.4] | 0 [0.0, 0.6] | 1 (0.9) [0.0, 4.9] | 1 (0.1) [0.0, 0.6] | 0 [0.0, 2.7] | 1 (0.1) [0.0, 0.5] |
| Pruritus allergic | 0 [0.0, 2.4] | 1 (0.2) [0.0, 0.9] | 0 [0.0, 3.2] | 1 (0.1) [0.0, 0.6] | 0 [0.0, 2.7] | 1 (0.1) [0.0, 0.5] |
| Psoriasis | 0 [0.0, 2.4] | 1 (0.2) [0.0, 0.9] | 0 [0.0, 3.2] | 1 (0.1) [0.0, 0.6] | 0 [0.0, 2.7] | 1 (0.1) [0.0, 0.5] |
| Rash generalised | 0 [0.0, 2.4] | 1 (0.2) [0.0, 0.9] | 0 [0.0, 3.2] | 1 (0.1) [0.0, 0.6] | 0 [0.0, 2.7] | 1 (0.1) [0.0, 0.5] |
| Skin disorder | 1 (0.6) [0.0, 3.6] | 0 [0.0, 0.6] | 0 [0.0, 3.2] | 1 (0.1) [0.0, 0.6] | 0 [0.0, 2.7] | 1 (0.1) [0.0, 0.5] |
| Skin exfoliation | 0 [0.0, 2.4] | 1 (0.2) [0.0, 0.9] | 0 [0.0, 3.2] | 1 (0.1) [0.0, 0.6] | 0 [0.0, 2.7] | 1 (0.1) [0.0, 0.5] |
| Skin lesion | 0 [0.0, 2.4] | 1 (0.2) [0.0, 0.9] | 0 [0.0, 3.2] | 1 (0.1) [0.0, 0.6] | 0 [0.0, 2.7] | 1 (0.1) [0.0, 0.5] |
| Skin ulcer | 1 (0.6) [0.0, 3.6] | 0 [0.0, 0.6] | 0 [0.0, 3.2] | 1 (0.1) [0.0, 0.6] | 0 [0.0, 2.7] | 1 (0.1) [0.0, 0.5] |
| Swelling face | 0 [0.0, 2.4] | 1 (0.2) [0.0, 0.9] | 0 [0.0, 3.2] | 1 (0.1) [0.0, 0.6] | 0 [0.0, 2.7] | 1 (0.1) [0.0, 0.5] |
| Xanthelasma | 1 (0.6) [0.0, 3.6] | 0 [0.0, 0.6] | 0 [0.0, 3.2] | 1 (0.1) [0.0, 0.6] | 0 [0.0, 3.2] | 1 (0.1) [0.0, 0.5] |
| **Injury, poisoning and procedural complications** | 6 (3.8) [1.4, 8.4] | 29 (4.5) [3.0, 6.4] | 0 [0.0, 3.2] | 35 (3.8)[2.7, 5.3] | 8 (5.7)[2.5, 11.3] | 43 (4.1) [2.9, 5.5] |
| Fall | 3 (1.9) [0.4, 5.6] | 3 (0.5) [0.1, 1.4] | 0 [0.0, 3.2] | 6 (0.7) [0.2, 1.4] | 3 (2.1) [0.4, 6.3] | 9 (0.9) [0.4, 1.6] |
| Ligament sprain | 0 [0.0, 2.4] | 4 (0.6) [0.2, 1.6] | 0 [0.0, 3.2] | 4 (0.4) [0.1, 1.1] | 1 (0.7) [0.0, 4.0] | 5 (0.5) [0.2, 1.1] |

26

| **SOC, Preferred term, n (IR/ 100 PY) (95% CI)** | **FCAS N= 42** | **MWS N=169** | **NOMID N= 32** | **All CAPS N=243** | **Other* N= 42** | **ALL N=285** |
|---|---|---|---|---|---|---|
| Vaccination complication | 0 [0.0, 2.4] | 4 (0.6) [0.2, 1.6] | 0 [0.0, 3.2] | 4 (0.4) [0.1, 1.1] | 0 [0.0, 3.2] | 4 (0.4) [0.1, 0.1] |
| Procedural headache | 0 [0.0, 2.4] | 3 (0.5) [0.1, 1.4] | 0 [0.0, 3.2] | 3 (0.3) [0.1, 1.0] | 0 [0.0, 2.7] | 3 (0.3) [0.1, 0.8] |
| Procedural nausea | 0 [0.0, 2.4] | 3 (0.5) [0.1, 1.4 | 0 [0.0, 3.2] | 3 (0.3) [0.1, 1.0] | 0 [0.0, 2.7] | 3 (0.3) [0.1, 0.8] |
| Eschar | 0 [0.0, 2.4] | 0 [0.0, 0.6] | 0 [0.0, 3.2] | 0 [0.0, 0.4] | 2 (1.4) [0.2, 5.2] | 2 (0.2) [0.0, 0.7] |
| Ankle fracture | 0 [0.0, 2.4] | 1 (0.2) [0.0, 0.9] | 0 [0.0, 3.2] | 1 (0.1) [0.0, 0.6] | 0 [0.0, 2.7] | 1 (0.1) [0.0, 0.5] |
| Clavicle fracture | 0 [0.0, 2.4] | 1 (0.2) [0.0, 0.9] | 0 [0.0, 3.2] | 1 (0.1) [0.0, 0.6] | 0 [0.0, 2.7] | 1 (0.1) [0.0, 0.5] |
| Contusion | 0 [0.0, 2.4] | 1 (0.2) [0.0, 0.9] | 0 [0.0, 3.2] | 1 (0.1) [0.0, 0.6] | 0 [0.0, 2.7] | 1 (0.1) [0.0, 0.5] |
| Facial bones fracture | 0 [0.0, 2.4] | 1 (0.2) [0.0, 0.9] | 0 [0.0, 3.2] | 1 (0.1) [0.0, 0.6] | 0 [0.0, 2.7] | 1 (0.1) [0.0, 0.5] |
| Femur fracture | 0 [0.0, 2.4] | 0 [0.0, 0.6] | 0 [0.0, 3.2] | 0 [0.0, 0.4] | 1 (0.7) [0.0, 4.0] | 1 (0.1) [0.0, 0.5] |
| Forearm fracture | 0 [0.0, 2.4] | 1 (0.2) [0.0, 0.9] | 0 [0.0, 3.2] | 1 (0.1) [0.0, 0.6] | 0 [0.0, 2.7] | 1 (0.1) [0.0, 0.5] |
| Hand fracture | 0 [0.0, 2.4] | 1 (0.2) [0.0, 0.9] | 0 [0.0, 3.2] | 1 (0.1) [0.0, 0.6] | 0 [0.0, 2.7] | 1 (0.1) [0.0, 0.5] |
| Nail avulsion | 0 [0.0, 2.4] | 1 (0.2) [0.0, 0.9] | 0 [0.0, 3.2] | 1 (0.1) [0.0, 0.6] | 0 [0.0, 2.7] | 1 (0.09) [0.0, 0.5] |
| Overdose | 1 (0.6) [0.0, 3.6] | 0 [0.0, 0.6] | 0 [0.0, 3.2] | 1 (0.1) [0.0, 0.6] | 0 [0.0, 2.7] | 1 (0.1) [0.0, 0.5] |
| Post procedural complication | 0 [0.0, 2.4] | 1 (0.2) [0.0, 0.9] | 0 [0.0, 3.2] | 1 (0.1) [0.0, 0.6] | 0 [0.0, 2.7] | 1 (0.1) [0.0, 0.5] |
| Post procedural diarrhoea | 0 [0.0, 2.4] | 1 (0.2) [0.0, 0.9] | 0 [0.0, 3.2] | 1 (0.1) [0.0, 0.6] | 0 [0.0, 2.7] | 1 (0.1) [0.0, 0.5] |

| SOC, Preferred term, n (IR/ 100 PY) (95% CI) | FCAS N= 42 | MWS N=169 | NOMID N= 32 | All CAPS N=243 | Other* N= 42 | ALL N=285 |
|---|---|---|---|---|---|---|
| Post procedural haemorrhage | 0 [0.0, 2.4] | 1 (0.2) [0.0, 0.9] | 0 [0.0, 3.2] | 1 (0.1) [0.0, 0.6] | 0 [0.0, 2.7] | 1 (0.1) [0.0, 0.5] |
| Procedural pain | 0 [0.0, 2.4] | 1 (0.2) [0.00, 0.9] | 0 [0.0, 3.2] | 1 (0.1) [0.00, 0.6] | 0 [0.0, 2.7] | 1 (0.1) [0.0, 0.5] |
| Radius fracture | 1 (0.6) [0.0, 3.6] | 0 [0.0, 0.6] | 0 [0.0, 3.2] | 1 (0.1) [0.0, 0.6] | 0 [0.0, 2.7] | 1 (0.1) [0.0, 0.5] |
| Rib fracture | 0 [0.0, 2.4] | 1 (0.2) [0.00, 0.9] | 0 [0.0, 3.2] | 1 (0.1) [0.00, 0.6] | 0 [0.0, 2.7] | 1 (0.1) [0.0, 0.5] |
| Tibia fracture | 0 [0.0, 2.4] | 0 [0.0, 0.6] | 0 [0.0, 3.2] | 0 [0.0, 0.4] | 1 (0.7) [0.0, 4.0] | 1 (0.1) [0.0, 0.5] |
| Vaginal laceration | 1 (0.6) [0.0, 3.6] | 0 [0.0, 0.6] | 0 [0.0, 3.2] | 1 (0.1) [0.0, 0.6] | 0 [0.0, 2.7] | 1 (0.1) [0.0, 0.5] |
| **Eye disorders** | 4 (2.6) [0.7, 6.6] | 20 (3.1) [1.9, 4.8] | 5 (4.4) [1.4, 10.3] | 29 (3.2) [2.1, 4.5] | 5 (3.6) [1.2, 8.3] | 34 (3.2) [2.2, 4.5] |
| Cataract | 0 [0.0, 2.4] | 4 (0.6) [0.2, 1.6] | 0 [0.0, 3.2] | 4 (0.4) [0.1, 1.1] | 0 [0.0, 2.7] | 4 (0.4) [0.1, 0.2] |
| Iritis | 1 (0.6) [0.0, 3.6] | 2 (0.3) [0.0, 1.1] | 0 [0.0, 3.2] | 3 (0.3) [0.1, 1.0] | 0 [0.0, 2.7] | 3 (0.3) [0.1, 0.8] |
| Uveitis | 0 [0.0, 2.4] | 1 (0.2) [0.0, 0.9] | 1 ( 0.9) [0.0, 4.9] | 2 (0.2) [0.0, 0.8] | 1 (0.7) [0.0, 4.0] | 3 (0.3) [0.1, 0.8] |
| Eye inflammation | 0 [0.0, 2.4] | 1 (0.2) [0.0, 0.9] | 0 [0.0, 3.2] | 1 (0.1) [0.0, 0.6] | 1 (0.7) [0.0, 4.0] | 2 (0.2) [0.0, 0.7] |
| Eye pruritus | 0 [0.0, 2.4] | 1 (0.2) [0.0, 0.9] | 1 (0.9) [0.0, 5.0] | 2 (0.2) [0.0, 0.8] | 0 [0.0, 2.7] | 2 (0.2) [0.0, 0.7] |
| Glaucoma | 0 [0.0, 2.4] | 1 (0.2) [0.0, 0.9] | 1 (0.9) [0.0, 4.9] | 2 (0.2) [0.0, 0.8] | 0 [0.0, 2.7] | 2 (0.2) [0.0, 0.7] |
| Iridocyclitis | 2 (1.3) [0.2, 4.6] | 0 [0.0, 0.6] | 0 [0.0, 3.2] | 2 (0.2) [0.0, 0.8] | 0 [0.0, 2.7] | 2 (0.2) [0.0, 0.7] |
| Ocular hyperaemia | 0 [0.0, 2.4] | 1 (0.2) [0.0, 0.9] | 0 [0.0, 3.2] | 1 (0.1) [0.0, 0.6] | 1 (0.7) [0.0, 4.0] | 2 (0.2) [0.0, 0.7] |

| SOC, Preferred term, n (IR/ 100 PY) (95% CI) | FCAS N= 42 | MWS N=169 | NOMID N= 32 | All CAPS N=243 | Other* N= 42 | ALL N=285 |
|---|---|---|---|---|---|---|
| Papilloedema | 0 [0.0, 2.4] | 2 (0.3) [0.0, 1.1] | 0 [0.0, 3.2] | 2 (0.2) [0.0, 0.8] | 0 [0.0, 2.7] | 2 (0.2) [0.0, 0.7] |
| Arcus lipoides | 1 (0.6) [0.0, 3.6] | 0 [0.0, 0.6] | 0 [0.0, 3.2] | 1 (0.1) [0.0, 0.6] | 0 [0.0, 2.7] | 1 (0.1) [0.0, 0.5] |
| Blepharitis | 0 [0.0, 2.4] | 1 (0.2) [0.0, 0.9] | 0 [0.0, 3.2] | 1 (0.1) [0.0, 0.6] | 0 [0.0, 2.7] | 1 (0.1) [0.0, 0.5] |
| Blindness | 0 [0.0, 2.4] | 1 (0.2) [0.0, 0.9] | 0 [0.0, 3.2] | 1 (0.1) [0.0, 0.6] | 0 [0.0, 2.7] | 1 (0.1) [0.0, 0.5] |
| Conjunctival hyperaemia | 0 [0.0, 2.4] | 0 0 [0.0, 0.6] | 1 (0.9) [0.0, 4.9] | 1 (0.1) [0.0, 0.6] | 0 [0.0, 2.7] | 1 (0.1) [0.0, 0.5] |
| Diplopia | 0 [0.0, 2.4] | 0 0 [0.0, 0.6] | 1 (0.9) [0.0, 4.9] | 1 (0.1) [0.0, 0.6] | 0 [0.0, 2.7] | 1 (0.1) [0.0, 0.5] |
| Dry eye | 0 [0.0, 2.4] | 0 0 [0.0, 0.6] | 0 [0.0, 3.2] | 0 [0.0, 0.4] | 1 (0.72) [0.0, 4.0] | 1 (0.1) [0.0, 0.5] |
| Episcleritis | 0 [0.0, 2.4] | 1 (0.2) [0.0, 0.9] | 0 [0.0, 3.2] | 1 (0.1) [0.0, 0.61 | 0 [0.0, 2.7] | 1 (0.1) [0.0, 0.5] |
| Eye pain | 0 [0.0, 2.4] | 1 (0.2) [0.0, 0.9] | 0 [0.0, 3.2] | 1 (0.1) [0.0, 0.6] | 0 [0.0, 2.7] | 1 (0.1) [0.0, 0.5] |
| Eyelid rash | 0 [0.0, 2.4] | 0 0 [0.0, 0.6] | 0 [0.0, 3.2] | 0 [0.0, 0.4] | 1 (0.7) [0.0, 4.0] | 1 (0.1) [0.0, 0.5] |
| Lacrimation increased | 0 [0.0, 2.4] | 1 (0.2) [0.0, 0.9] | 0 [0.0, 3.2] | 1 (0.1) [0.0, 0.6] | 0 [0.0, 2.7] | 1 (0.1) [0.0, 0.5] |
| Photophobia | 0 [0.0, 2.4] | 1 (0.2) [0.0, 0.9] | 0 [0.0, 3.2] | 1 (0.1) [0.0, 0.6] | 0 [0.0, 2.7] | 1 (0.1) [0.0, 0.5] |
| Visual acuity reduced | 0 [0.0, 2.4] | 1 (0.2) [0.0, 0.9] | 0 [0.0, 3.2] | 1 (0.1) [0.0, 0.6] | 0 [0.0, 2.7] | 1 (0.1) [0.0, 0.5] |
| **Blood and lymphatic system disorders** | 0 [0.0, 2.4] | 9 (1.4) [0.6, 2.6] | 1(0.9) [0.0, 4.9] | 10 (1.1) [0.5, 2.0] | 5 (3.6) [1.2, 8.4] | 15 (1.4) [0.8, 2.3] |
| Anaemia | 0 [0.0, 2.4] | 3 (0.5) [0.1, 1.4] | 0 [0.0, 3.2] | 3 (0.3) [0.1, 1.0] | 0 [0.0, 2.7] | 3 (0.3) [0.1, 0.8] |
| Iron deficiency anaemia | 0 [0.0, 2.4] | 2 (0.3) [0.0, 1.1] | 0 [0.0, 3.2] | 2 (0.2) [0.0, 0.8] | 0 [0.0, 2.7] | 2 (0.2) [0.0, 0.7] |

| SOC, Preferred term, n (IR/ 100 PY) (95% CI) | FCAS N= 42 | MWS N=169 | NOMID N= 32 | All CAPS N=243 | Other* N= 42 | ALL N=285 |
|---|---|---|---|---|---|---|
| Lymphadenopathy | 0 [0.0, 2.4] | 2 (0.3) [0.0, 1.1] | 0 [0.0, 3.2] | 2 (0.2) [0.0, 0.8] | 0 [0.0, 2.7] | 2 (0.2) [0.0, 0.7] |
| Lymphopenia | 0 [0.0, 2.4] | 0 [0.0, 0.6] | 0 [0.0, 3.2] | 0 [0.0, 0.4] | 2 (1.4) [0.2, 5.2 | 2 (0.2) [0.0, 0.7] |
| Thrombocytopenia | 0 [0.0, 2.4] | 1 (0.2) [0.0, 0.9] | 0 [0.0, 3.2] | 1 (0.1) [0.0, 0.6] | 1 (0.7) [0.0, 4.0] | 2 (0.2) [0.0, 0.7] |
| Increased tendency to bruise | 0 [0.0, 2.4] | 0 [0.0, 0.6] | 1 (0.9) [0.0, 4.9] | 1 (0.1) [0.0, 0.6] | 0 [0.0, 2.7] | 1 (0.1) [0.0, 0.5] |
| Lymphocytic infiltration | 0 [0.0, 2.4] | 1 (0.2) [0.0, 0.9] | 0 [0.0, 3.2] | 1 (0.1) [0.0, 0.6] | 0 [0.0, 2.7] | 1 (0.1) [0.0, 0.5] |
| Microcytosis | 0 [0.0, 2.4] | 0 [0.0, 0.6] | 0 [0.0, 3.2] | 0 [0.0, 0.4] | 1 (0.7) [0.0, 4.0] | 1 (0.1) [0.0, 0.5] |
| Neutropenia | 0 [0.0, 2.4] | 0 [0.0, 0.6] | 0 [0.0, 3.2] | 0 [0.0, 0.4] | 1 (0.7) [0.0, 4.0] | 1 (0.1) [0.0, 0.5] |
| **Neoplasms benign, malignant and unspecified (incl cysts and polyps)** | 1 (0.6) [0.0, 3.6] | 13 (2.0) [1.1, 3.4] | 0 [0.0, 3.2] | 14 (1.5) [0.8, 2.6] | 1 (0.7) [0.0, 4.0] | 15 (1.4) [0.8, 2.3] |
| Rectal cancer | 0 [0.0, 2.4] | 3 (0.5) [0.1, 1.4] | 0 [0.0, 3.2] | 3 (0.3) [0.1, 1.0] | 0 [0.0, 2.7] | 3 (0.3) [0.1, 0.8] |
| Melanocyte naevus | 0 [0.0, 2.4] | 1 (0.2) [0.0, 0.9] | 0 [0.0, 3.2] | 1 (0.1) [0.0, 0.6] | 1 (0.7) [0.0, 4.0] | 2 (0.2) [0.0, 0.7] |
| Uterine leiomyoma | 0 [0.0, 2.4] | 2 (0.3) [0.0, 1.1] | 0 [0.0, 3.2] | 2 (0.2) [0.0, 0.8] | 0 [0.0, 2.7] | 2 (0.2) [0.0, 0.7] |
| Adenocarcinoma | 0 [0.0, 2.4] | 1 (0.2) [0.0, 0.9] | 0 [0.0, 3.2] | 1 (0.1) [0.0, 0.6] | 0 [0.0, 2.7] | 1 (0.1) [0.0, 0.5] |
| Basal cell carcinoma | 1 (0.6) [0.0, 3.6] | 0 [0.0, 0.6] | 0 [0.0, 3.2] | 1 (0.1) [0.0, 0.6] | 0 [0.0, 2.7] | 1 (0.1) [0.0, 0.5] |
| Gastrointestinal neoplasm | 0 [0.0, 2.4] | 1 (0.2) [0.0, 0.9] | 0 [0.0, 3.2] | 1 (0.1) [0.0, 0.6] | 0 [0.0, 2.7] | 1 (0.1) [0.0, 0.5] |

| SOC, Preferred term, n (IR/ 100 PY) (95% CI) | FCAS N= 42 | MWS N=169 | NOMID N= 32 | All CAPS N=243 | Other* N= 42 | ALL N=285 |
|---|---|---|---|---|---|---|
| Lipoma | 0 [0.0, 2.4] | 1 (0.2) [0.0, 0.9] | 0 [0.0, 3.2] | 1 (0.1) [0.0, 0.6] | 0 [0.0, 2.7] | 1 (0.1) [0.0, 0.5] |
| Meningioma | 0 [0.0, 2.4] | 1 (0.2) [0.0, 0.9] | 0 [0.0, 3.2] | 1 (0.1) [0.0, 0.6] | 0 [0.0, 2.7] | 1 (0.1) [0.0, 0.5] |
| Neurilemmoma benign | 0 [0.0, 2.4] | 1 (0.2) [0.0, 0.9] | 0 [0.0, 3.2] | 1 (0.1) [0.0, 0.6] | 0 [0.0, 2.7] | 1 (0.1) [0.0, 0.5] |
| Neurofibroma | 0 [0.0, 2.4] | 1 (0.2) [0.0, 0.9] | 0 [0.0, 3.2] | 1 (0.1) [0.0, 0.6] | 0 [0.0, 2.7] | 1 (0.1) [0.0, 0.5] |
| Prostate cancer | 0 [0.0, 2.4] | 1 (0.2) [0.0, 0.9] | 0 [0.0, 3.2] | 1 (0.1) [0.0, 0.6] | 0 [0.0, 2.7] | 1 (0.1) [0.0, 0.5] |
| **Cardiac disorders** | 0 [0.0, 2.4] | 11 (1.7) [0.9, 3.0] | 0 [0.0, 3.2] | 11 (1.2) [0.6, 2.1] | 3 (2.2) [0.4, 6.3] | 14 (1.3) [0.7, 2.2] |
| Angina pectoris | 0 [0.0, 2.4] | 5 (0.8) [0.3, 1.8] | 0 [0.0, 3.2] | 5 (0.5) [0.2, 1.3] | 0 [0.0, 2.7] | 5 (0.5) [0.2, 1.1] |
| Pericardial effusion | 0 [0.0, 2.4] | 1 (0.2) [0.0, 0.9] | 0 [0.0, 3.2] | 1 (0.1) [0.0, 0.6] | 1 (0.7) [0.02, 4.0] | 2 (0.2) [0.0, 0.7] |
| Coronary artery disease | 0 [0.0, 2.4] | 1 (0.2) [0.0, 0.9] | 0 [0.0, 3.2] | 1 (0.1) [0.0, 0.6] | 0 [0.0, 2.7] | 1 (0.1) [0.0, 0.5] |
| Extrasystoles | 0 [0.0, 2.4] | 1 (0.2) [0.0, 0.9] | 0 [0.0, 3.2] | 1 (0.1) [0.0, 0.6] | 0 [0.0, 2.7] | 1 (0.1) [0.0, 0.5] |
| Mitral valve incompetence | 0 [0.0, 2.4] | 1 (0.2) [0.0, 0.9] | 0 [0.0, 3.2] | 1 (0.1) [0.0, 0.6] | 0 [0.0, 2.7] | 1 (0.1) [0.0, 0.5] |
| Palpitations | 0 [0.0, 2.4] | 0 [0.0, 0.6] | 0 [0.0, 3.2] | 0 [0.0, 0.4] | 1 (0.7) [0.02, 4.0] | 1 (0.1) [0.0, 0.5] |
| Supraventricular extrasystoles | 0 [0.0, 2.4] | 1 (0.2) [0.0, 0.9] | 0 [0.0, 3.2] | 1 (0.1) [0.0, 0.6] | 0 [0.0, 2.7] | 1 (0.1) [0.0, 0.5] |
| Tachycardia | 0 [0.0, 2.4] | 1 (0.2) [0.0, 0.9] | 0 [0.0, 3.2] | 1 (0.1) [0.0, 0.6] | 0 [0.0, 2.7] | 1 (0.1) [0.0, 0.5] |
| Ventricular tachycardia | 0 [0.0, 2.4] | 0 [0.0, 0.6] | 0 [0.0, 3.2] | 0 [0.0, 0.4] | 1 (0.7) [0.0, 4.0] | 1 (0.1) [0.0, 0.5] |

| SOC, Preferred term, n (IR/ 100 PY) (95% CI) | FCAS N= 42 | MWS N=169 | NOMID N= 32 | All CAPS N=243 | Other* N= 42 | ALL N=285 |
|---|---|---|---|---|---|---|
| **Vascular disorders** | 1 (0.6) [0.0, 3.6] | 11 (1.7) [0.8, 3.0] | 0 [0.0, 3.2] | 12 (1.3) [0.7, 2.3] | 1 (0.7) [0.0, 4.0] | 13 (1.2) [0.7, 2.1] |
| Hypertension | 1 (0.6) [0.0, 3.6] | 2 (0.3) [0.0, 1.1] | 0 [0.0, 3.2] | 3 (0.3) [0.1, 1.0] | 0 [0.0, 2.7] | 3 (0.3) [0.1, 0.8] |
| Peripheral venous disease | 0 [0.0, 2.4] | 2 (0.3) [0.0, 1.1] | 0 [0.0, 3.2] | 2 (0.2) [0.0, 0.8] | 0 [0.0, 2.7] | 2 (0.2) [0.0, 0.7] |
| Circulatory collapse | 0 [0.0, 2.4] | 1 (0.2) [0.0, 0.9] | 0 [0.0, 3.2] | 1 (0.1) [0.0, 0.6] | 0 [0.0, 2.7] | 1 (0.1) [0.0, 0.5] |
| Haematoma | 0 [0.0, 2.4] | 0 [0.0, 0.6] | 0 [0.0, 3.2] | 0 [0.0, 0.4] | 1 (0.7) [0.0, 4.0] | 1 (0.1) [0.0, 0.5] |
| Hot flush | 0 [0.0, 2.4] | 1 (0.2) [0.0, 0.9] | 0 [0.0, 3.2] | 1 (0.1) [0.0, 0.6] | 0 [0.0, 2.7] | 1 (0.1) [0.0, 0.5] |
| Lymphoedema | 0 [0.0, 2.4] | 1 (0.2) [0.0, 0.9] | 0 [0.0, 3.2] | 1 (0.1) [0.0, 0.6] | 0 [0.0, 2.7] | 1 (0.1) [0.0, 0.5] |
| Peripheral arterial occlusive disease | 0 [0.0, 2.4] | 1 (0.2) [0.0, 0.9] | 0 [0.0, 3.2] | 1 (0.1) [0.0, 0.6] | 0 [0.0, 2.7] | 1 (0.1) [0.0, 0.5] |
| Phlebitis | 0 [0.0, 2.4] | 1 (0.2) [0.0, 0.9] | 0 [0.0, 3.2] | 1 (0.1) [0.0, 0.6] | 0 [0.0, 2.7] | 1 (0.1) [0.0, 0.5] |
| Post thrombotic syndrome | 0 [0.0, 2.4] | 1 (0.2) [0.0, 0.9] | 0 [0.0, 3.2] | 1 (0.1) [0.0, 0.6] | 0 [0.0, 2.7] | 1 (0.1) [0.0, 0.5] |
| Vein disorder | 0 [0.0, 2.4] | 1 (0.2) [0.0, 0.9] | 0 [0.0, 3.2] | 1 (0.1) [0.0, 0.6] | 0 [0.0, 2.7] | 1 (0.1) [0.0, 0.5] |
| **Psychiatric disorders** | 0 [0.0, 2.4] | 9 (1.4) [0.6, 2.6] | 1 (0.9) [0.0, 4.9] | 10 (1.1) [0.5, 2.0] | 2 (1.4) [0.2, 5.2] | 12 (1.1) [0.6, 2.0] |
| Depression | 0 [0.0, 2.4] | 4 (0.6) [0.2 1.6] | 1 (0.9) [0.0, 4.9] | 5 (0.5) [0.2 1.3] | 0 [0.0, 2.7] | 5 (0.5) [0.2, 1.1] |
| Insomnia | 0 [0.0, 2.4] | 2 (0.3) [0.0, 1.1] | 0 [0.0, 3.2] | 2 (0.2) [0.0, 0.8] | 0 [0.0, 2.7] | 2 (0.2) [0.0, 0.7] |
| Agitation | 0 [0.0, 2.4] | 1 (0.2) [0.0, 0.9] | 0 [0.0, 3.2] | 1 (0.1) [0.0, 0.6] | 0 [0.0, 2.7] | 1 (0.1) [0.0, 0.5] |

| SOC, Preferred term, n (IR/ 100 PY) (95% CI) | FCAS N= 42 | MWS N=169 | NOMID N= 32 | All CAPS N=243 | Other* N= 42 | ALL N=285 |
|---|---|---|---|---|---|---|
| Anxiety | 0 [0.0, 2.4] | 1 (0.2) [0.0, 0.9] | 0 [0.0, 3.2] | 1 (0.1) [0.0, 0.6] | 0 [0.0, 2.7] | 1 (0.1) [0.0, 0.5] |
| Fear of injection | 0 [0.0, 2.4] | 1 (0.2) [0.0, 0.9] | 0 [0.0, 3.2] | 1 (0.1) [0.0, 0.6] | 0 [0.0, 2.7] | 1 (0.1) [0.0, 0.5] |
| Nervousness | 0 [0.0, 2.4] | 0 [0.0, 0.6] | 0 [0.0, 3.2] | 0 [0.0, 0.4] | 1 (0.7) [0.0, 4.0] | 1 (1.0) [0.0, 0.5] |
| Somnambulism | 0 [0.0, 2.4] | 0 [0.0, 0.6] | 0 [0.0, 3.2] | 0 [0.0, 0.4] | 1 (0.7) [0.0, 4.0] | 1 (1.0) [0.0, 0.5] |
| **Congenital, familial and genetic disorders** | 0 [0.0, 2.4] | 4 (0.6) [0.2, 1.6] | 0 [0.0, 3.2] | 4 (0.4) [0.1, 1.1] | 6 (4.3) [1.6, 9.3] | 10 (0.9) [0.5, 1.7] |
| Cryopyrin associated periodic syndrome | 0 [0.0, 2.4] | 3 (0.5) [0.1, 1.4] | 0 [0.0, 3.2] | 3 (0.3) [0.1, 1.0] | 2 (1.4) [0.2, 5.2] | 5 (0.5) [0.2, 1.1] |
| Familial Mediterranean fever | 0 [0.0, 2.4] | 0 [0.0, 0.6] | 0 [0.0, 3.2] | 0 [0.0, 0.4] | 3 (2.2) [0.4, 6.3] | 3 (0.3) [0.1, 0.8] |
| Gilbert's syndrome | 0 [0.0, 2.4] | 0 [0.0, 0.6] | 0 [0.0, 3.2] | 0 [0.0, 0.4] | 1 (0.7) [0.0, 4.0] | 1 (0.1) [0.0, 0.5] |
| Hereditary neuropathy with liability to pressure palsies | 0 [0.0, 2.4] | 1 (0.2) [0.0, 0.9] | 0 [0.0, 3.2] | 1 (0.1) [0.0, 0.6] | 0 [0.0, 2.7] | 1 (0.1) [0.0, 0.5] |
| **Hepatobiliary disorders** | 0 [0.0, 2.4] | 5 (0.8) [0.3, 1.8] | 0 [0.0, 3.2] | 5 (0.5) [0.2, 1.3] | 4 (2.9) [0.8, 7.3] | 9 (0.9) [0.4, 1.6] |
| Cholelithiasis | 0 [0.0, 2.4] | 3 (0.5) [0.1, 1.4] | 0 [0.0, 3.2] | 3 (0.3) [0.1, 1.0] | 0 [0.0, 2.7] | 3 (0.3) [0.1, 0.8] |
| Autoimmune hepatitis | 0 [0.0, 2.4] | 0 [0.0, 0.6] | 0 [0.0, 3.2] | 0 [0.0, 0.4] | 1 (0.7) [0.0, 4.0] | 1 (0.1) [0.0, 0.5] |
| Cholecystitis | 0 [0.0, 2.4] | 1 (0.2) [0.0, 0.9] | 0 [0.0, 3.2] | 1 (0.1) [0.0, 0.6] | 0 [0.0, 2.7] | 1 (0.1) [0.0, 0.5] |

| SOC, Preferred term, n (IR/ 100 PY) (95% CI) | FCAS N= 42 | MWS N=169 | NOMID N= 32 | All CAPS N=243 | Other* N= 42 | ALL N=285 |
|---|---|---|---|---|---|---|
| Cholestasis | 0 [0.0, 2.4] | 0 [0.0, 0.6] | 0 [0.0, 3.2] | 0 [0.0, 0.4] | 1 (0.7) [0.0, 4.0] | 1 (0.1) [0.0, 0.5] |
| Hepatic steatosis | 0 [0.0, 2.4] | 1 (0.2) [0.0, 0.9] | 0 [0.0, 3.2] | 1 (0.1) [0.0, 0.6] | 0 [0.0, 2.7] | 1 (0.1) [0.0, 0.5] |
| Hepatitis | 0 [0.0, 2.4] | 0 [0.0, 0.6] | 0 [0.0, 3.2] | 0 [0.0, 0.4] | 1 (0.7) [0.0, 4.0] | 1 (0.1) [0.0, 0.5] |
| Hyperbilirubinaemia | 0 [0.0, 2.4] | 0 [0.0, 0.6] | 0 [0.0, 3.2] | 0 [0.0, 0.4] | 1 (0.7) [0.0, 4.0] | 1 (0.1) [0.0, 0.5] |
| **Immune system disorders** | 1 (0.6) [0.0, 3.6] | 4 (0.6) [0.2, 1.6] | 1 (0.9) [0.0, 4.9] | 6 (0.7) [0.2, 1.4] | 3 (2.1) [0.4, 6.3] | 9 (0.9) [0.4, 1.6] |
| Hypersensitivity | 0 [0.0, 2.4] | 3 (0.5) [0.1, 1.4] | 1 (0.9) [0.0, 4.9] | 4 (0.4) [0.1, 1.1] | 0 [0.0, 2.7] | 4 (0.4) [0.1, 1.0] |
| Seasonal allergy | 1 (0.6) [0.0, 3.6] | 1 (0.2) [0.0, 0.9] | 0 [0.0, 3.2] | 2 (0.2) [0.0, 0.8] | 1(0.7) [0.0, 4.0] | 3 (0.3) [0.1, 0.8] |
| Allergy to arthropod bite | 0 [0.0, 2.4] | 0 [0.0, 0.6] | 0 [0.0, 3.2] | 0 [0.0, 0.4] | 1 (0.7) [0.0, 4.0] | 1 (0.1) [0.0, 0.5] |
| Drug hypersensitivity | 0 [0.0, 2.4] | 0 [0.0, 0.6] | 0 [0.0, 3.2] | 0 [0.0, 0.4] | 1 (0.7) [0.0, 4.0] | 1 (1.0) [0.0, 0.5] |
| **Reproductive system and breast disorders** | 0 [0.0, 2.4] | 8 (1.2) [0.5, 2.4] | 0 [0.0, 3.2] | 8 (0.9) [0.4, 1.7] | 0 [0.0, 2.7] | 8 (0.8) [0.3, 1.5] |
| Menstruation irregular | 0 [0.0, 2.4] | 2 (0.3) [0.0, 1.1] | 0 [0.0, 3.2] | 2 (0.2) [0.0, 0.8] | 0 [0.0, 2.7] | 2 (0.2) [0.0, 0.7] |
| Breast mass | 0 [0.0, 2.4] | 1 (0.2) [0.0, 0.9] | 0 [0.0, 3.2] | 1 (0.1) [0.0, 0.6] | 0 [0.0, 2.7] | 1 (0.1) [0.0, 0.5] |
| Endometriosis | 0 [0.0, 2.4] | 1 (0.2) [0.0, 0.9] | 0 [0.0, 3.2] | 1 (0.1) [0.0, 0.6] | 0 [0.0, 2.7] | 1 (0.1) [0.0, 0.5] |
| Menopausal symptoms | 0 [0.0, 2.4] | 1 (0.2) [0.0, 0.9] | 0 [0.0, 3.2] | 1 (0.1) [0.0, 0.6] | 0 [0.0, 2.7] | 1 (0.1) [0.0, 0.5] |
| Prostatitis | 0 [0.0, 2.4] | 1 (0.2) [0.0, 0.9] | 0 [0.0, 3.2] | 1 (0.1) [0.0, 0.6] | 0 [0.0, 2.7] | 1 (0.1) [0.0, 0.5] |

| SOC, Preferred term, n (IR/ 100 PY) (95% CI) | FCAS N= 42 | MWS N=169 | NOMID N= 32 | All CAPS N=243 | Other* N= 42 | ALL N=285 |
|---|---|---|---|---|---|---|
| Uterine polyp | 0 [0.0, 2.4] | 1 (0.2) [0.0, 0.9] | 0 [0.0, 3.2] | 1 (0.1) [0.0, 0.6] | 0 [0.0, 2.7] | 1 (0.1) [0.0, 0.5] |
| Vulvovaginal dryness | 0 [0.0, 2.4] | 1 (0.2) [0.0, 0.9] | 0 [0.0, 3.2] | 1 (0.1) [0.0, 0.6] | 0 [0.0, 2.7] | 1 (0.1) [0.0, 0.5] |
| **Metabolism and nutritional disorders** | 1 (0.6) [0.0, 3.6] | 4 (0.6) [0.2, 1.6] | 0 [0.0, 3.2] | 5 (0.5) [0.2, 1.3] | 0 [0.0, 2.7] | 5 (0.5) [0.2, 1.1] |
| Diabetes mellitus | 0 [0.0, 2.4] | 2 (0.3) [0.0, 1.1] | 0 [0.0, 3.2] | 2 (0.2) [0.0, 0.8] | 0 [0.0, 2.7] | 2 (0.2) [0.0, 0.7] |
| Decreased appetite | 0 [0.0, 2.4] | 1 (0.2) [0.0, 0.9] | 0 [0.0, 3.2] | 1 (0.1) [0.0, 0.6] | 0 [0.0, 2.7] | 1 (0.1) [0.0, 0.5] |
| Increased appetite | 0 [0.0, 2.4] | 1 (0.2) [0.0, 0.9] | 0 [0.0, 3.2] | 1 (0.1) [0.0, 0.6] | 0 [0.0, 2.7] | 1 (0.1) [0.0, 0.5] |
| Type 2 diabetes mellitus | 1 (0.6) [0.0, 3.6] | 0 [0.0, 0.6] | 0 [0.0, 3.2] | 1 (0.1) [0.0, 0.6] | 0 [0.0, 2.7] | 1 (0.1) [0.0, 0.5] |
| **Pregnancy, puerperium and perinatal conditions** | 0 [0.0, 2.4] | 5 (0.8) [0.3, 1.8] | 0 [0.0, 3.2] | 5 (0.5) [0.2, 1.3] | 0 [0.0, 2.7] | 5 (0.5) [0.2, 1.1] |
| Abortion spontaneous | 0 [0.0, 2.4] | 3 (0.5) [0.1, 1.4] | 0 [0.0, 3.2] | 3 (0.3) [0.1, 1.0] | 0 [0.0, 2.7] | 3 (0.3) [0.1, 0.8] |
| Gestational diabetes | 0 [0.0, 2.4] | 1 (0.2) [0.0, 0.9] | 0 [0.0, 3.2] | 1 (0.1) [0.0, 0.6] | 0 [0.0, 2.7] | 1 (0.1) [0.0, 0.5] |
| Pregnancy | 0 [0.0, 2.4] | 1 (0.2) [0.0, 0.9] | 0 [0.0, 3.2] | 1 (0.1) [0.0, 0.6] | 0 [0.0, 2.7] | 1 (0.1) [0.0, 0.5] |
| **Endocrine disorders** | 1 (0.6) [0.0, 3.6] | 2 (0.3) [0.0, 1.1] | 0 [0.0, 3.2] | 3 (0.3) [0.1, 1.0] | 1 (0.7) [0.0, 4.0] | 4 (0.4) [0.1, 1.0] |
| Addison's disease | 1 (0.6) [0.0, 3.6] | 0 [0.0, 0.6] | 0 [0.0, 3.2] | 1 (0.1) [0.0, 0.6] | 0 [0.0, 2.7] | 1 (0.1) [0.0, 0.5] |
| Cushingoid | 0 [0.0, 2.4] | 0 [0.0, 0.6] | 0 [0.0, 3.2] | 0 [0.0, 0.4] | 1 (0.7) [0.0, 4.0] | 1 (0.1) [0.0, 0.5] |
| Hypothyroidism | 0 [0.0, 2.4] | 1 (0.2) [0.0, 0.9] | 0 [0.0, 3.2] | 1 (0.1) [0.0, 0.6] | 0 [0.0, 2.7] | 1 (0.1) [0.0, 0.5] |

| SOC, Preferred term, n (IR/ 100 PY) (95% CI) | FCAS N= 42 | MWS N=169 | NOMID N= 32 | All CAPS N=243 | Other* N= 42 | ALL N=285 |
|---|---|---|---|---|---|---|
| Thyroid cyst | 0 [0.0, 2.4] | 1 (0.2) [0.0, 0.9] | 0 [0.0, 3.2] | 1 (0.1) [0.0, 0.6] | 0 [0.0, 2.7] | 1 (0.1) [0.0, 0.5] |
| **Renal and urinary disorders** | 0 [0.0, 2.4] | 4 (0.6) [0.2, 1.6] | 0 [0.0, 3.2] | 4 (0.4) [0.1, 1.1] | 0 [0.0, 2.7] | 4 (0.4) [0.1,1.0] |
| Proteinuria | 0 [0.0, 2.4] | 2 (0.3) [0.0, 1.1] | 0 [0.0, 3.2] | 2 (0.2) [0.0, 0.8] | 0 [0.0, 2.7] | 2 (0.2) [0.0, 0.7] |
| Enuresis | 0 [0.0, 2.4] | 1 (0.2) [0.0, 0.9] | 0 [0.0, 3.2] | 1 (0.1) [0.0, 0.6] | 0 [0.0, 2.7] | 1 (0.1) [0.0, 0.5] |
| Renal failure | 0 [0.0, 2.4] | 1 (0.2) [0.0, 0.9] | 0 [0.0, 3.2] | 1 (0.1) [0.0, 0.6] | 0 [0.0, 2.7] | 1 (0.1) [0.0, 0.5] |

*Other includes atypical CAPS (n=18) and non-CAPS diagnoses (n=24); non-CAPS diagnoses include systemic juvenile idiopathic arthritis, unspecified autoinflammatory syndromes, familial Mediterranean fever, mevalonate kinase deficiency, adult-onset Still's disease, tumour necrosis factor-receptor associated periodic syndrome, Erdheim–Chester disease, Blau syndrome, and granulomatosis with polyangiitis.

AE, adverse events; CAPS, cryopyrin-associated periodic syndromes; FCAS, familial cold autoinflammatory syndrome; IL, interleukin; IR, incidence rates; MWS, Muckle–Wells syndrome; n, number of events; NOMID, neonatal-onset multisystem inflammatory disease; PY, patient-years; SOC, system organ class.

**Table S6** Frequency of adverse events by baseline canakinumab dose

| | FCAS (N=42) | MWS (N=169) | NOMID (N=32) | All CAPS (N=243) | Other* (N=42) | ALL (N=285) |
|---|---|---|---|---|---|---|
| **Adverse events, % (m/n)** | | | | | | |
| <2mg/kg | 25.0 (4/16) | 90.7 (39/43) | 50.0 (1/2) | 72.1 (44/61) | 77.8 (7/9) | 72.9 (51/70) |
| 2–<4mg/kg | 81.8 (18/22) | 78.3 (65/83) | 83.3 (15/18) | 79.7 (98/123) | 83.3 (20/24) | 80.3 (118/147) |
| 4–<8mg/kg | | 81.0 (17/21) | 87.5 (7/8) | 82.8 (24/29) | 100 (4/4) | 84.9 (28/33) |
| >8mg/kg | | 0 (0/1) | 100 (3/3) | 75.0 (3/4) | | 75.0 (3/4) |
| Missing | 100 (4/4) | 61.9 (13/21) | 100 (1/1) | 69.2 (18/26) | 100 (5/5) | 74.2 (23/31) |
| **Serious adverse events, % (m/n)** | | | | | | |
| <2mg/kg | 0 (0/16) | 39.5 (17/43) | 0 (0/2) | 27.9 (17/61) | 33.3 (3/9) | 28.6 (20/70) |
| 2–<4mg/kg | 13.6 (3/22) | 30.1 (25/83) | 33.3 (6/18) | 27.6 (34/123) | 29.2 (7/24) | 27.9 (41/147) |
| 4–<8mg/kg | | 33.3 (7/21) | 25.0 (2/8) | 31.0 (9/29) | 75.0 (3/4) | 36.4 (12/33) |
| >8mg/kg | | 0 (0/1) | 0 (0/3) | 0 (0/4) | | 0 (0/4) |
| Missing | 0 (0/4) | 33.3 (7/21) | 100 (1/1) | 30.8 (8/26) | 40.0 (2/5) | 32.3 (10/31) |

% values are calculated based on the number of patients within the respective dose category (m/n); *Other includes atypical CAPS (n=18) and non-CAPS diagnoses (n=24); non-CAPS diagnoses include systemic juvenile idiopathic arthritis, unspecified autoinflammatory syndromes, familial Mediterranean fever, mevalonate kinase deficiency, adult-onset Still's disease, tumour necrosis factor-receptor associated periodic syndrome, Erdheim–Chester disease, Blau syndrome, and granulomatosis with polyangiitis.



Walker UA, *et al. RMD Open* 2021; 7:e001663. doi: 10.1136/rmdopen-2021-001663

CAPS, cryopyrin-associated periodic syndromes; FCAS, familial cold autoinflammatory syndrome; m, number of patients who reported at least one event; MWS, Muckle–Wells syndrome; n, number of patients within the respective dose category data; NOMID, neonatal-onset multisystem inflammatory disease.

**Table S7** Most common adverse events related to canakinumab per investigator assessment

| **System organ class**<br>**Preferred term** | **All CAPS**<br>**(N=243)** | **All**<br>**(N=285)** |
|---|---|---|
| **Any AE, n (IR/ 100 PY) [95% CI]** | 305 (33.2) [29.6, 37.2] | 335 (31.7) [28.4, 35.2) |
| **Infections and infestations** | 126 (13.7) [11.4, 16.3] | 140 (13.2) [11.1, 15.6] |
| Nasopharyngitis | 15 (1.6) [0.9, 2.7] | 16 (1.5) [0.9, 2.5] |
| Lower respiratory tract infection | 13 (1.4) [0.8, 2.4] | 13 (1.2) [0.7, 2.1] |
| Urinary tract infection | 11 (1.2) [0.6, 2.1] | 11 (1.0) [0.5, 1.9] |
| Tonsillitis | 8 (0.9) [0.4, 1.7] | 10 (0.9) [0.5, 1.7] |
| Influenza | 9 (1.0) [0.4, 1.9] | 9 (0.9) [0.4, 1.6] |
| Bronchitis | 8 (0.9) [0.4, 1.7] | 8 (0.8) [0.3, 1.5] |
| Gastroenteritis | 4 (0.4) [0.1, 1.1] | 5 (0.5) [0.2, 1.1] |
| Pneumonia | 4 (0.4) [0.1, 1.1] | 5 (0.5) [0.2, 1.1] |
| Ear infection | 4 (0.4) [0.1, 1.1] | 4 (0.4) [0.1, 1.0] |
| Rhinitis | 4 (0.4) [0.1, 1.1] | 4 (0.4) [0.1, 1.0] |
| Viral infection | 2 (0.2) [0, 0.8] | 4 (0.4) [0.1, 1.0] |
| Cellulitis | 3 (0.3) [0.1, 1.0] | 3 (0.3) [0.1, 0.8] |
| Herpes zoster | 3 (0.3) [0.1, 1.0] | 3 (0.3) [0.1, 0.8] |
| Hordeolum | 3 (0.3) [0.1, 1.0] | 3 (0.3) [0.1, 0.8] |
| Laryngitis | 3 (0.3) [0.1, 1.0] | 3 (0.3) [0.1, 0.8] |
| Upper respiratory tract infection | 1 (0.1) [0.0, 0.6] | 3 (0.3) [0.1, 0.8) |
| **General disorders and administration site conditions** | 33 (3.6) [2.5, 5.0] | 37 (3.5) [2.5, 4.8] |
| Fatigue | 5 (0.5) [0.2, 1.3] | 6 (0.6) [0.2, 1.2] |
| Pyrexia | 5 (0.5) [0.2, 1.3] | 5 (0.5) [0.2, 1.1] |

| System organ class<br>Preferred term | All CAPS<br>(N=243) | All<br>(N=285) |
|---|---|---|
| Vaccination site inflammation | 4 (0.4) (0.1, 1.1] | 5 (0.5) [0.2, 1.1] |
| Condition aggravated | 3 (0.3) [0.1, 1.0] | 3 (0.3) [0.1, 0.8] |
| Injection site reaction | 3 (0.3) [0.1, 1.0] | 3 (0.3) [0.1, 0.8] |
| Malaise | 2 (0.2) [0.0, 0.8] | 3 (0.3) [0.1, 0.8] |
| **Ear and labyrinth disorders** | 20 (2.2) [1.3, 3.4] | 20 (1.9) [1.2, 2.9] |
| Vertigo | 16 (1.7) [1.0, 2.8] | 16 (1.5) [0.9, 2.5] |
| **Gastrointestinal disorders** | 18 (2.0) [1.2, 3.1] | 20 (1.9) [1.2, 2.9] |
| Abdominal pain upper | 5 (0.5) [0.2, 1.3] | 5 (0.5) [0.2, 1.1] |
| Diarrhea | 4 (0.4) [0.1, 1.1] | 4 (0.4) [0.1, 1.0] |
| Abdominal pain | 3 (0.3) [0.1, 1.0] | 3 (0.3) [0.1, 0.8) |
| **Skin and subcutaneous tissue disorders** | 17 (1.9) [1.1, 3.0] | 19 (1.8) [1.1, 2.8] |
| Rash | 3 (0.3) [0.1, 1.0] | 4 (0.4) [0.1, 1.0] |
| **Investigations** | 17 (1.9) [1.1, 3.0] | 17 (1.6) [0.9, 2.6] |
| Weight increased | 8 (0.9) [0.4, 1.7] | 8 (0.8) [0.3, 1.5] |
| **Injury, poisoning and procedural complications** | 16 (1.7) [1.0, 2.8] | 16 (1.5) [0.9, 2.5] |
| Vaccination complication | 4 (0.4) [0.1, 1.1] | 4 (0.4) [0.1, 1.0] |
| Procedural headache | 3 (0.3) [0.1, 1.0] | 3 (0.3) [0.1, 0.8] |
| Procedural nausea | 3 (0.3) [0.1, 1.0] | 3 (0.3) [0.1, 0.8] |
| **Nervous system disorders** | 16 (1.7) [1.0, 2.8] | 16 (1.5) [0.9, 2.5] |
| Headache | 7 (0.8) [0.3, 1.6] | 7 (0.7) [0.3, 1.4] |
| Syncope | 3 (0.3) [0.1, 1.0] | 3 (0.3) [0.1, 0.8] |

| System organ class<br>Preferred term | All CAPS<br>(N=243) | All<br>(N=285) |
|---|---|---|
| **Respiratory, thoracic and mediastinal disorders** | 13 (1.4) [0.8, 2.4] | 14 (1.3) [0.7, 2.2] |
| Cough | 5 (0.5) [0.2, 1.3] | 5 (0.5) [0.2, 1.1] |
| Oropharyngeal pain | 4 (0.4) [0.1, 1.1] | 5 (0.5) [0.2, 1.1] |
| **Eye disorders** | 7 (0.8) [0.3, 1.6] | 8 (0.8) [0.3, 1.5] |

Listed are study emergent events starting after baseline with an absolute frequency of ≥5 events per system organ class and ≥ 3 events per preferred term in the all registry patients group.

AE, adverse event; CAPS, cryopyrin-associated periodic syndromes; CI, confidence interval; IR, incidence rate; n, number of events; PY, patient-years.

**Table S8** Serious adverse events by preferred term by patient group (n=1 in all registry patients)

| **Preferred term**<br><br>**n (IR/ 100 PY)**<br><br>**(95% CI)** | **FCAS (n=42)** | **MWS (n=169)** | **NOMID (n=32)** | **All CAPS (n=243)** | **Other* (n=42)** | **All (n=285)** |
|---|---|---|---|---|---|---|
| **All events** | 4 (2.6) [0.7, 6.6] | 100 (15.4) [12.6, 18.8] | 24 (21.1) [13.5, 31.4] | 128 (14.0) [11.6,16.6] | 27 (19.3) [12.7, 28.1] | 155 (14.7) [12.4, 17.2] |
| Anaemia | 0 [0.0, 2.4] | 1 (0.2) [0.0, 0.9] | 0 [0.0, 3.2] | 1 (0.1) [0.0, 0.6] | 0 [0.0, 2.7] | 1 (0.1) [0.0, 0.5] |
| Abdominal pain upper | 0 [0.0, 2.4] | 1 (0.2) [0.0, 0.9] | 0 [0.0, 3.2] | 1 (0.1) [0.0, 0.6] | 0 [0.0, 2.7] | 1 (0.1) [0.00, 0.5] |
| Abscess limb | 0 [0.0, 2.4] | 1 (0.2) [0.0, 0.9] | 0 [0.0, 3.2] | 1 (0.1) [0.0, 0.6] | 0 [0.0, 2.7] | 1 (0.1) [0.0, 0.5] |
| Acute respiratory distress syndrome | 0 [0.0, 2.4] | 0 [0.0, 0.6] | 0 [0.0, 3.2] | 0 [0.0, 0.4] | 1 (0.7) [0.0, 4.0] | 1 (0.1) [0.0, 0.5] |
| Adenocarcinoma | 0 [0.0, 2.4] | 1 (0.2) [0.0, 0.9] | 0 [0.0, 3.2] | 1 (0.1) [0.0, 0.6] | 0 [0.0, 2.7] | 1 (0.1) [0.0, 0.5] |
| Appendicitis | 0 [0.0, 2.4] | 1 (0.2) [0.0, 0.9] | 0 [0.0, 3.2] | 1 (0.1) [0.0, 0.6] | 0 [0.0, 2.7] | 1 (0.1) [0.0, 0.5] |
| Arthralgia | 0 [0.0, 2.4] | 1 (0.2) [0.0, 0.9] | 0 [0.0, 3.2] | 1 (0.1) [0.0, 0.6] | 0 [0.0, 2.7] | 1 (0.1) [0.0, 0.5] |
| Autoimmune hepatitis | 0 [0.0, 2.4] | 0 [0.0, 0.6] | 0 [0.0, 3.2] | 0 [0.0, 0.4] | 1 (0.7) [0.0, 4.0] | 1 (0.1) [0.0, 0.5] |
| Bacterial tracheitis | 0 | 1 (0.2) | 0 | 1 (0.1) [0.0, 0.6] | 0 | 1 (0.1) [0.0, 0.5] |

| **Preferred term**<br><br>**n (IR/ 100 PY)**<br><br>**(95% CI)** | **FCAS (n=42)** | **MWS (n=169)** | **NOMID (n=32)** | **All CAPS (n=243)** | **Other* (n=42)** | **All (n=285)** |
|---|---|---|---|---|---|---|
| | [0.0, 2.4] | [0.0, 0.9] | [0.0, 3.2] | | [0.0, 2.7] | |
| Blindness | 0<br>[0.0, 2.4] | 1 (0.2)<br>[0.0, 0.9] | 0<br>[0.0, 3.2] | 1 (0.1)<br>[0.0, 0.6] | 0<br>[0.0, 2.7] | 1 (0.1)<br>[0.0, 0.5] |
| Brain mass | 0<br>[0.0, 2.4] | 1 (0.2)<br>[0.0, 0.9] | 0<br>[0.0, 3.2] | 1 (0.1)<br>[0.0, 0.6] | 0<br>[0.0, 2.7] | 1 (0.1)<br>[0.0, 0.5] |
| Bursitis | 0<br>[0.0, 2.4] | 0<br>[0.0, 0.6] | 0<br>[0.0, 3.2] | 0<br>[0.0, 0.4] | 1 (0.7)<br>[0.0, 4.0] | 1 (0.1)<br>[0.0, 0.5] |
| Cerebral infarction | 1 (0.6)<br>[0.0, 3.6] | 0<br>[0.0, 0.6] | 0<br>[0.0, 3.2] | 1 (0.1)<br>[0.0, 0.6] | 0<br>[0.0, 2.7] | 1 (0.1)<br>[0.0, 0.5] |
| Cholecystitis | 0<br>[0.0, 2.4] | 1 (0.2)<br>[0.0, 0.9] | 0<br>[0.0, 3.2] | 1 (0.1)<br>[0.0, 0.6] | 0<br>[0.0, 2.7] | 1 (0.1)<br>[0.0, 0.5] |
| Cholestasis | 0<br>[0.0, 2.4] | 0<br>[0.0, 0.6] | 0<br>[0.0, 3.2] | 0<br>[0.0, 0.4] | 1 (0.7)<br>[0.0, 4.0] | 1 (0.1)<br>[0.0, 0.5] |
| Chronic sinusitis | 0<br>[0.0, 2.4] | 1 (0.2)<br>[0.0, 0.9] | 0<br>[0.0, 3.2] | 1 (0.1)<br>[0.0, 0.6] | 0<br>[0.0, 2.7] | 1 (0.1)<br>[0.0, 0.5] |
| Circulatory collapse | 0<br>[0.0, 2.4] | 1 (0.2)<br>[0.0, 0.9] | 0<br>[0.0, 3.2] | 1 (0.1)<br>[0.0, 0.6] | 0<br>[0.0, 2.7] | 1 (0.1)<br>[0.0, 0.5] |
| Conjunctivitis | 0<br>[0.0, 2.4] | 1 (0.2)<br>[0.0, 0.9] | 0<br>[0.0, 3.2] | 1 (0.1)<br>[0.0, 0.6] | 0<br>[0.0, 2.7] | 1 (0.1)<br>[0.0, 0.5] |

| **Preferred term**<br><br>**n (IR/ 100 PY)**<br><br>**(95% CI)** | **FCAS (n=42)** | **MWS (n=169)** | **NOMID (n=32)** | **All CAPS (n=243)** | **Other* (n=42)** | **All (n=285)** |
|---|---|---|---|---|---|---|
| Constipation | 0 [0.0, 2.4] | 1 (0.2) [0.0, 0.9] | 0 [0.0, 3.2] | 1 (0.1) [0.0, 0.6] | 0 [0.0, 2.7] | 1 (0.1) [0.0, 0.5] |
| Coronary artery disease | 0 [0.0, 2.4] | 1 (0.2) [0.0, 0.9] | 0 [0.0, 3.2] | 1 (0.1) [0.0, 0.6] | 0 [0.0, 2.7] | 1 (0.1) [0.0, 0.5] |
| Cough | 0 [0.0, 2.4] | 1 (0.2) [0.0, 0.9] | 0 [0.0, 3.2] | 1 (0.1) [0.0, 0.6] | 0 [0.0, 2.7] | 1 (0.1) [0.0, 0.5] |
| Deafness | 0 [0.0, 2.4] | 0 [0.0, 0.6] | 1 (0.9) [0.0, 4.9] | 1 (0.1) [0.0, 0.6] | 0 [0.0, 2.7] | 1 (0.1) [0.0, 0.5] |
| Demyelination | 0 [0.0, 2.4] | 1 (0.2) [0.0, 0.9] | 0 [0.0, 3.2] | 1 (0.1) [0.0, 0.6] | 0 [0.0, 2.7] | 1 (0.1) [0.0, 0.5] |
| Diabetes mellitus | 0 [0.0, 2.4] | 1 (0.2) [0.0, 0.9] | 0 [0.0, 3.2] | 1 (0.1) [0.0, 0.6] | 0 [0.0, 2.7] | 1 (0.1) [0.0, 0.5] |
| Diplopia | 0 [0.0, 2.4] | 0 [0.0, 0.6] | 1 (0.9) [0.0, 4.9] | 1 (0.1) [0.0, 0.6] | 0 [0.0, 2.7] | 1 (0.1) [0.0, 0.5] |
| Diverticulitis | 0 [0.0, 2.4] | 1 (0.2) [0.0, 0.9] | 0 [0.0, 3.2] | 1 (0.1) [0.0, 0.6] | 0 [0.0, 2.7] | 1 (0.1) [0.0, 0.5] |
| Drug hypersensitivity | 0 [0.0, 2.4] | 0 [0.0, 0.6] | 0 [0.0, 3.2] | 0 [0.0, 0.4] | 1 (0.7) [0.0, 4.0] | 1 (0.1) [0.0, 0.5] |
| Dyspnoea | 0 [0.0, 2.4] | 0 [0.0, 0.6] | 0 [0.0, 3.2] | 0 [0.0, 0.4] | 1 (0.7) [0.0, 4.0] | 1 (0.1) [0.0, 0.5] |

| **Preferred term**<br><br>**n (IR/ 100 PY)**<br><br>**(95% CI)** | **FCAS (n=42)** | **MWS (n=169)** | **NOMID (n=32)** | **All CAPS (n=243)** | **Other* (n=42)** | **All (n=285)** |
|---|---|---|---|---|---|---|
| Endometriosis | 0 [0.0, 2.4] | 1 (0.2) [0.0, 0.9] | 0 [0.0, 3.2] | 1 (0.1) [0.0, 0.6] | 0 [0.0, 2.7] | 1 (0.1) [0.0, 0.5] |
| Enterocolitis | 0 [0.0, 2.4] | 0 [0.0, 0.6] | 0 [0.0, 3.2] | 0 [0.0, 0.4] | 1 (0.7) [0.0, 4.0] | 1 (0.1) [0.0, 0.5] |
| Epilepsy | 0 [0.0, 2.4] | 0 [0.0, 0.6] | 1 (0.9) [0.0, 4.9] | 1 (0.1) [0.0, 0.6] | 0 [0.0, 2.7] | 1 (0.1) [0.0, 0.5] |
| Epstein-Barr virus infection | 0 [0.0, 2.4] | 0 [0.0, 0.6] | 0 [0.0, 3.2] | 0 [0.0, 0.4] | 1 (0.7) [0.0, 4.0] | 1 (0.1) [0.0, 0.5] |
| Erysipelas | 0 [0.0, 2.4] | 0 [0.0, 0.6] | 1 (0.9) [0.0, 4.9] | 1 (0.1) [0.0, 0.6] | 0 [0.0, 2.7] | 1 (0.1) [0.0, 0.5] |
| Facial bone fracture | 0 [0.0, 2.4] | 1 (0.2) [0.0, 0.9] | 0 [0.0, 3.2] | 1 (0.1) [0.0, 0.6] | 0 [0.0, 2.7] | 1 (0.1) [0.0, 0.5] |
| Familial Mediterranean fever | 0 [0.0, 2.4] | 0 [0.0, 0.6] | 0 [0.0, 3.2] | 0 [0.0, 0.4] | 1 (0.7) [0.0, 4.0] | 1 (0.1) [0.0, 0.5] |
| Femur fracture | 0 [0.0, 2.4] | 0 [0.0, 0.6] | 0 [0.0, 3.2] | 0 [0.0, 0.4] | 1 (0.7) [0.0, 4.0] | 1 (0.1) [0.0, 0.5] |
| Gastroenteritis | 0 [0.0, 2.4] | 0 [0.0, 0.6] | 0 [0.0, 3.2] | 0 [0.0, 0.4] | 1 (0.7) [0.0, 4.0] | 1 (0.1) [0.0, 0.5] |
| Gastroenteritis rotavirus | 0 [0.0, 2.4] | 0 [0.0, 0.6] | 0 [0.0, 3.2] | 0 [0.0, 0.4] | 1 (0.7) [0.0, 4.0] | 1 (0.1) [0.0, 0.5] |

| **Preferred term**<br><br>**n (IR/ 100 PY)**<br><br>**(95% CI)** | **FCAS (n=42)** | **MWS (n=169)** | **NOMID (n=32)** | **All CAPS (n=243)** | **Other* (n=42)** | **All (n=285)** |
|---|---|---|---|---|---|---|
| Gastrointestinal neoplasm | 0 [0.0, 2.4] | 1 (0.2) [0.0, 0.9] | 0 [0.0, 3.2] | 1 (0.1) [0.0, 0.6] | 0 [0.0, 2.7] | 1 (0.1) [0.0, 0.5] |
| Gastrointestinal toxicity | 0 [0.0, 2.4] | 0 [0.0, 0.6] | 0 [0.0, 3.2] | 0 [0.0, 0.4] | 1 (0.7) [0.0, 4.0] | 1 (0.1) [0.0, 0.5] |
| Gastrooesophageal reflux disease | 0 [0.0, 2.4] | 1 (0.2) [0.0, 0.9] | 0 [0.0, 3.2] | 1 (0.1) [0.0, 0.6] | 0 [0.0, 2.7] | 1 (0.1) [0.0, 0.5] |
| Haemoglobin decreased | 0 [0.0, 2.4] | 1 (0.2) [0.0, 0.9] | 0 [0.0, 3.2] | 1 (0.1) [0.0, 0.6] | 0 [0.0, 2.7] | 1 (0.1) [0.0, 0.5] |
| Hepatitis | 0 [0.0, 2.4] | 0 [0.0, 0.6] | 0 [0.0, 3.2] | 0 [0.0, 0.4] | 1 (0.7) [0.0, 4.0] | 1 (0.1) [0.0, 0.5] |
| Herpes zoster | 0 [0.0, 2.4] | 1 (0.2) [0.0, 0.9] | 0 [0.0, 3.2] | 1 (0.1) [0.0, 0.6] | 0 [0.0, 2.7] | 1 (0.1) [0.0, 0.5] |
| Hyperhidrosis | 0 [0.0, 2.4] | 1 (0.2) [0.0, 0.9] | 0 [0.0, 3.2] | 1 (0.1) [0.0, 0.6] | 0 [0.0, 2.7] | 1 (0.1) [0.0, 0.5] |
| Impaired healing | 0 [0.0, 2.4] | 0 [0.0, 0.6] | 1 (0.9) [0.0, 4.9] | 1 (0.1) [0.0, 0.6] | 0 [0.0, 2.7] | 1 (0.1) [0.0, 0.5] |
| Infection susceptibility increased | 0 [0.0, 2.4] | 1 (0.2) [0.0, 0.9] | 0 [0.0, 3.2] | 1 (0.1) [0.0, 0.6] | 0 [0.0, 2.7] | 1 (0.1) [0.0, 0.5] |
| Influenza | 0 [0.0, 2.4] | 1 (0.2) [0.0, 0.9] | 0 [0.0, 3.2] | 1 (0.1) [0.0, 0.6] | 0 [0.0, 2.7] | 1 (0.1) [0.0, 0.5] |



Walker UA, *et al. RMD Open* 2021; 7:e001663. doi: 10.1136/rmdopen-2021-001663

| Preferred term<br><br>n (IR/ 100 PY)<br><br>(95% CI) | FCAS<br>(n=42) | MWS<br>(n=169) | NOMID<br>(n=32) | All CAPS<br>(n=243) | Other*<br>(n=42) | All<br>(n=285) |
|---|---|---|---|---|---|---|
| Inguinal hernia | 0<br>[0.0, 2.4] | 0<br>[0.0, 0.6] | 0<br>[0.0, 3.2] | 0<br>[0.0, 0.4] | 1 (0.7)<br>[0.0, 4.0] | 1 (0.1)<br>[0.0, 0.5] |
| Intervertebral disc protrusion | 0<br>[0.0, 2.4] | 1 (0.2)<br>[0.0, 0.9] | 0<br>[0.0, 3.2] | 1 (0.1)<br>[0.0, 0.6] | 0<br>[0.0, 2.7] | 1 (0.1)<br>[0.0, 0.5] |
| Localised oedema | 0<br>[0.0, 2.4] | 0<br>[0.0, 0.6] | 0<br>[0.0, 3.2] | 0<br>[0.0, 0.4] | 1 (0.7)<br>[0.0, 4.0] | 1 (0.1)<br>[0.0, 0.5] |
| Lumbar spinal stenosis | 0<br>[0.0, 2.4] | 1 (0.2)<br>[0.0, 0.9] | 0<br>[0.0, 3.2] | 1 (0.1)<br>[0.0, 0.6] | 0<br>[0.0, 2.7] | 1 (0.1)<br>[0.0, 0.5] |
| Lymphadenopathy | 0<br>[0.0, 2.4] | 1 (0.2)<br>[0.0, 0.9] | 0<br>[0.0, 3.2] | 1 (0.1)<br>[0.0, 0.6] | 0<br>[0.0, 2.7] | 1 (0.1)<br>[0.0, 0.5] |
| Malaise | 0<br>[0.0, 2.4] | 1 (0.2)<br>[0.0, 0.9] | 0<br>[0.0, 3.2] | 1 (0.1)<br>[0.0, 0.6] | 0<br>[0.0, 2.7] | 1 (0.1)<br>[0.0, 0.5] |
| Meningitis aseptic | 0<br>[0.0, 2.4] | 0<br>[0.0, 0.6] | 1 (0.9)<br>[0.0, 4.9] | 1 (0.1)<br>[0.0, 0.6] | 0<br>[0.0, 2.7] | 1 (0.1)<br>[0.0, 0.5] |
| Meningitis bacterial | 0<br>[0.0, 2.4] | 1 (0.2)<br>[0.0, 0.9] | 0<br>[0.0, 3.2] | 1 (0.1)<br>[0.0, 0.6] | 0<br>[0.0, 2.7] | 1 (0.1)<br>[0.0, 0.5] |
| Mitral valve incompetence | 0<br>[0.0, 2.4] | 1 (0.2)<br>[0.0, 0.9] | 0<br>[0.0, 3.2] | 1 (0.1)<br>[0.0, 0.6] | 0<br>[0.0, 2.7] | 1 (0.1)<br>[0.0, 0.5] |
| Myalgia | 0<br>[0.0, 2.4] | 0<br>[0.0, 0.6] | 0<br>[0.0, 3.2] | 0<br>[0.0, 0.4] | 1 (0.7)<br>[0.0, 4.0] | 1 (0.1)<br>[0.0, 0.5] |

| **Preferred term**<br><br>**n (IR/ 100 PY)**<br><br>**(95% CI)** | **FCAS (n=42)** | **MWS (n=169)** | **NOMID (n=32)** | **All CAPS (n=243)** | **Other* (n=42)** | **All (n=285)** |
|---|---|---|---|---|---|---|
| Nervous system disorder | 0<br>[0.0, 2.4] | 1 (0.2)<br>[0.0, 0.9] | 0<br>[0.0, 3.2] | 1 (0.1)<br>[0.0, 0.6] | 0<br>[0.0, 2.7] | 1 (0.1)<br>[0.0, 0.5] |
| Neurilemmoma benign | 0<br>[0.0, 2.4] | 1 (0.2)<br>[0.0, 0.9] | 0<br>[0.0, 3.2] | 1 (0.1)<br>[0.0, 0.6] | 0<br>[0.0, 2.7] | 1 (0.1)<br>[0.0, 0.5] |
| Oral herpes | 0<br>[0.0, 2.4] | 1 (0.2)<br>[0.0, 0.9] | 0<br>[0.0, 3.2] | 1 (0.1)<br>[0.0, 0.6] | 0<br>[0.0, 2.7] | 1 (0.1)<br>[0.0, 0.5] |
| Overdose | 1 (0.6)<br>[0.0, 3.6] | 0<br>[0.0, 0.6] | 0<br>[0.0, 3.2] | 1 (0.1)<br>[0.0, 0.6] | 0<br>[0.0, 2.7] | 1 (0.1)<br>[0.0, 0.5] |
| Parvovirus B19 infection | 0<br>[0.0, 2.4] | 0<br>[0.0, 0.6] | 0<br>[0.0, 3.2] | 0<br>[0.0, 0.4] | 1 (0.7)<br>[0.0, 4.0] | 1 (0.1)<br>[0.0, 0.5] |
| Pericardial effusion | 0<br>[0.0, 2.4] | 0<br>[0.0, 0.6] | 0<br>[0.0, 3.2] | 0<br>[0.0, 0.4] | 1 (0.7)<br>[0.0, 4.0] | 1 (0.1)<br>[0.0, 0.5] |
| Periorbital cellulitis | 0<br>[0.0, 2.4] | 1 (0.2)<br>[0.0, 0.9] | 0<br>[0.0, 3.2] | 1 (0.1)<br>[0.0, 0.6] | 0<br>[0.0, 2.7] | 1 (0.1)<br>[0.0, 0.5] |
| Peripheral arterial occlusive disease | 0<br>[0.0, 2.4] | 1 (0.2)<br>[0.0, 0.9] | 0<br>[0.0, 3.2] | 1 (0.1)<br>[0.0, 0.6] | 0<br>[0.0, 2.7] | 1 (0.1)<br>[0.0, 0.5] |
| Pharyngitis | 0<br>[0.0, 2.4] | 1 (0.2)<br>[0.0, 0.9] | 0<br>[0.0, 3.2] | 1 (0.1)<br>[0.0, 0.6] | 0<br>[0.0, 2.7] | 1 (0.1)<br>[0.0, 0.5] |
| Postoperative wound infection | 0<br>[0.0, 2.4] | 0<br>[0.0, 0.6] | 0<br>[0.0, 3.2] | 0<br>[0.0, 0.4] | 1 (0.7)<br>[0.0, 4.0] | 1 (0.1)<br>[0.0, 0.5] |

| **Preferred term** **n (IR/ 100 PY)** **(95% CI)** | **FCAS (n=42)** | **MWS (n=169)** | **NOMID (n=32)** | **All CAPS (n=243)** | **Other* (n=42)** | **All (n=285)** |
|---|---|---|---|---|---|---|
| Post procedural haemorrhage | 0 [0.0, 2.4] | 1 (0.2) [0.0, 0.9] | 0 [0.0, 3.2] | 1 (0.1) [0.0, 0.6] | 0 [0.0, 2.7] | 1 (0.1) [0.0, 0.5] |
| Pregnancy | 0 [0.0, 2.4] | 1 (0.2) [0.0, 0.9] | 0 [0.0, 3.2] | 1 (0.1) [0.0, 0.6] | 0 [0.0, 2.7] | 1 (0.1) [0.0, 0.5] |
| Prostate cancer | 0 [0.0, 2.4] | 1 (0.2) [0.0, 0.9] | 0 [0.0, 3.2] | 1 (0.1) [0.0, 0.6] | 0 [0.0, 2.7] | 1 (0.1) [0.0, 0.5] |
| Radius fracture | 1 (0.6) [0.0, 3.6] | 0 [0.0, 0.6] | 0 [0.0, 3.2] | 1 (0.1) [0.0, 0.6] | 0 [0.0, 2.7] | 1 (0.1) [0.0, 0.5] |
| Rash | 0 [0.0, 2.4] | 1 (0.2) [0.0, 0.9] | 0 [0.0, 3.2] | 1 (0.1) [0.0, 0.6] | 0 [0.0, 2.7] | 1 (0.1) [0.0, 0.5] |
| Rash generalised | 0 [0.0, 2.4] | 1 (0.2) [0.0, 0.9] | 0 [0.0, 3.2] | 1 (0.1) [0.0, 0.6] | 0 [0.0, 2.7] | 1 (0.1) [0.0, 0.5] |
| Rib fracture | 0 [0.0, 2.4] | 1 (0.2) [0.0, 0.9] | 0 [0.0, 3.2] | 1 (0.1) [0.0, 0.6] | 0 [0.0, 2.7] | 1 (0.1) [0.0, 0.5] |
| Seizure | 0 [0.0, 2.4] | 1 (0.2) [0.0, 0.9] | 0 [0.0, 3.2] | 1 (0.1) [0.0, 0.6] | 0 [0.0, 2.7] | 1 (0.1) [0.0, 0.5] |
| Subcutaneous abscess | 0 [0.0, 2.4] | 1 (0.2) [0.0, 0.9] | 0 [0.0, 3.2] | 1 (0.1) [0.0, 0.6] | 0 [0.0, 2.7] | 1 (0.1) [0.0, 0.5] |
| Sudden hearing loss | 0 [0.0, 2.4] | 1 (0.2) [0.0, 0.9] | 0 [0.0, 3.2] | 1 (0.1) [0.0, 0.6] | 0 [0.0, 2.7] | 1 (0.1) [0.0, 0.5] |

| **Preferred term**<br><br>**n (IR/ 100 PY)**<br><br>**(95% CI)** | **FCAS (n=42)** | **MWS (n=169)** | **NOMID (n=32)** | **All CAPS (n=243)** | **Other* (n=42)** | **All (n=285)** |
|---|---|---|---|---|---|---|
| Syncope | 0 [0.0, 2.4] | 1 (0.2) [0.0, 0.9] | 0 [0.0, 3.2] | 1 (0.1) [0.0, 0.6] | 0 [0.0, 2.7] | 1 (0.1) [0.0, 0.5] |
| Synovial cyst | 0 [0.0, 2.4] | 1 (0.2) [0.0, 0.9] | 0 [0.0, 3.2] | 1 (0.1) [0.0, 0.6] | 0 [0.0, 2.7] | 1 (0.1) [0.0, 0.5] |
| Upper respiratory tract infection | 0 [0.0, 2.4] | 0 [0.0, 0.6] | 0 [0.0, 3.2] | 0 [0.0, 0.4] | 1 (0.7) [0.0, 4.0] | 1 (0.1) [0.0, 0.5] |
| Uterine leiomyoma | 0 [0.0, 2.4] | 1 (0.2) [0.0, 0.9] | 0 [0.0, 3.2] | 1 (0.1) [0.0, 0.6] | 0 [0.0, 2.7] | 1 (0.1) [0.0, 0.5] |
| Vaccination complication | 0 [0.0, 2.4] | 1 (0.2) [0.0, 0.9] | 0 [0.0, 3.2] | 1 (0.1) [0.0, 0.6] | 0 [0.0, 2.7] | 1 (0.1) [0.0, 0.5] |
| Vaccination site cellulitis | 0 [0.0, 2.4] | 1 (0.2) [0.0, 0.9] | 0 [0.0, 3.2] | 1 (0.1) [0.0, 0.6] | 0 [0.0, 2.7] | 1 (0.1) [0.0, 0.5] |
| Ventricular tachycardia | 0 [0.0, 2.4] | 0 [0.0, 0.6] | 0 [0.0, 3.2] | 0 [0.0, 0.4] | 1 (0.7) [0.0, 4.0] | 1 (0.1) [0.0, 0.5] |
| Vomiting | 0 [0.0, 2.4] | 0 [0.0, 0.6] | 0 [0.0, 3.2] | 0 [0.0, 0.4] | 1 (0.7) [0.0, 4.0] | 1 (0.1) [0.0, 0.5] |
| Wound infection | 0 [0.0, 2.4] | 1 (0.2) [0.0, 0.9] | 0 [0.0, 3.2] | 1 (0.1) [0.0, 0.6] | 0 [0.0, 2.7] | 1 (0.1) [0.0, 0.5] |

| Preferred term<br>n (IR/ 100 PY)<br>(95% CI) | FCAS<br>(n=42) | MWS<br>(n=169) | NOMID<br>(n=32) | All CAPS<br>(n=243) | Other*<br>(n=42) | All<br>(n=285) |
|---|---|---|---|---|---|---|

Number of events ≥2 in all registry patients are presented in **Table 2** of the manuscript

*Other includes atypical CAPS (n=18) and non-CAPS diagnoses (n=24); non-CAPS diagnoses include systemic juvenile idiopathic arthritis, unspecified autoinflammatory syndromes, familial Mediterranean fever, mevalonate kinase deficiency, adult-onset Still's disease, tumour necrosis factor-receptor associated periodic syndrome, Erdheim–Chester disease, Blau syndrome, and granulomatosis with polyangiitis.

CAPS, cryopyrin-associated periodic syndromes; CI, confidence interval; FCAS, familial cold autoinflammatory syndrome; IR, incidence rate; MWS, Muckle–Wells syndrome; n, number of events, NOMID, neonatal-onset multisystem inflammatory disease; PY, patient-years.

**Figure S1** Physician's Global Assessment of autoinflammatory disease activity and CRP/SAA levels over time

**A.** CAPS phenotypes

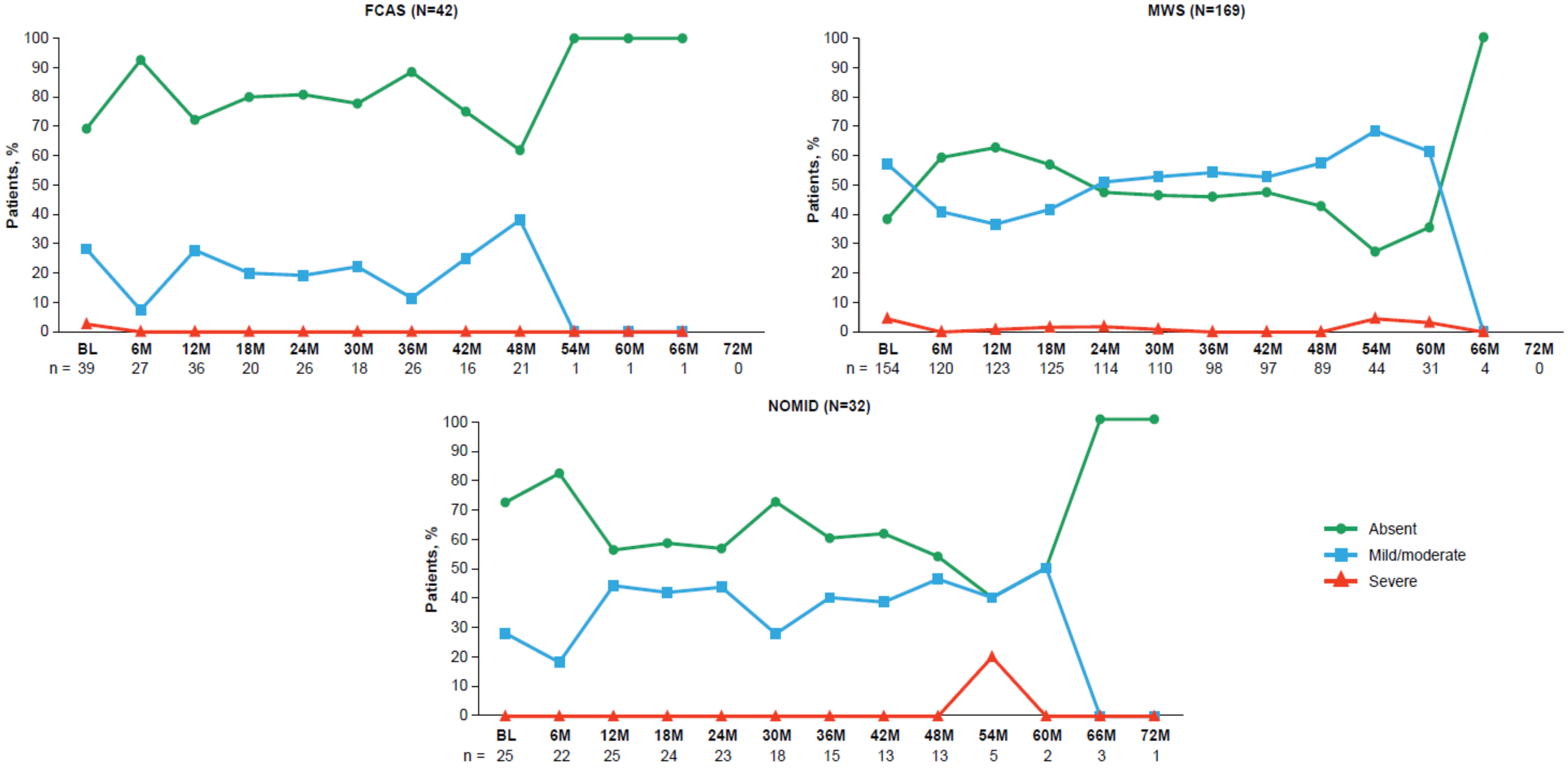

**B.** Exposure to anti–IL-1 treatment*

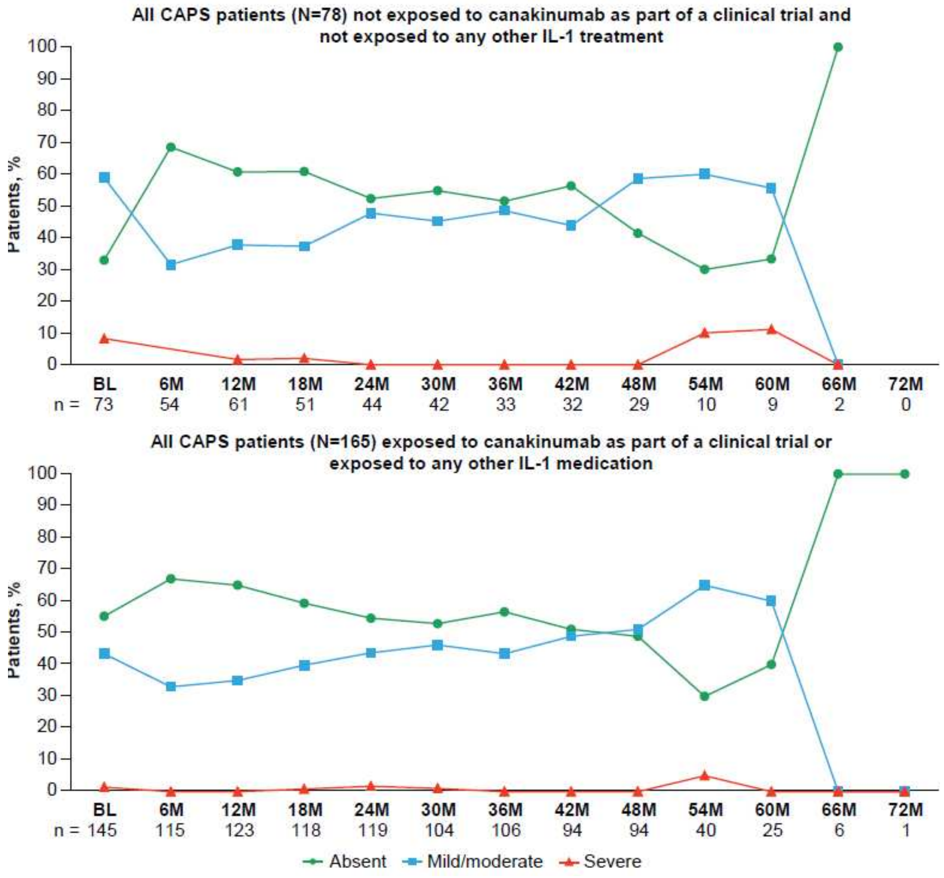

**C.** CRP and SAA levels over time

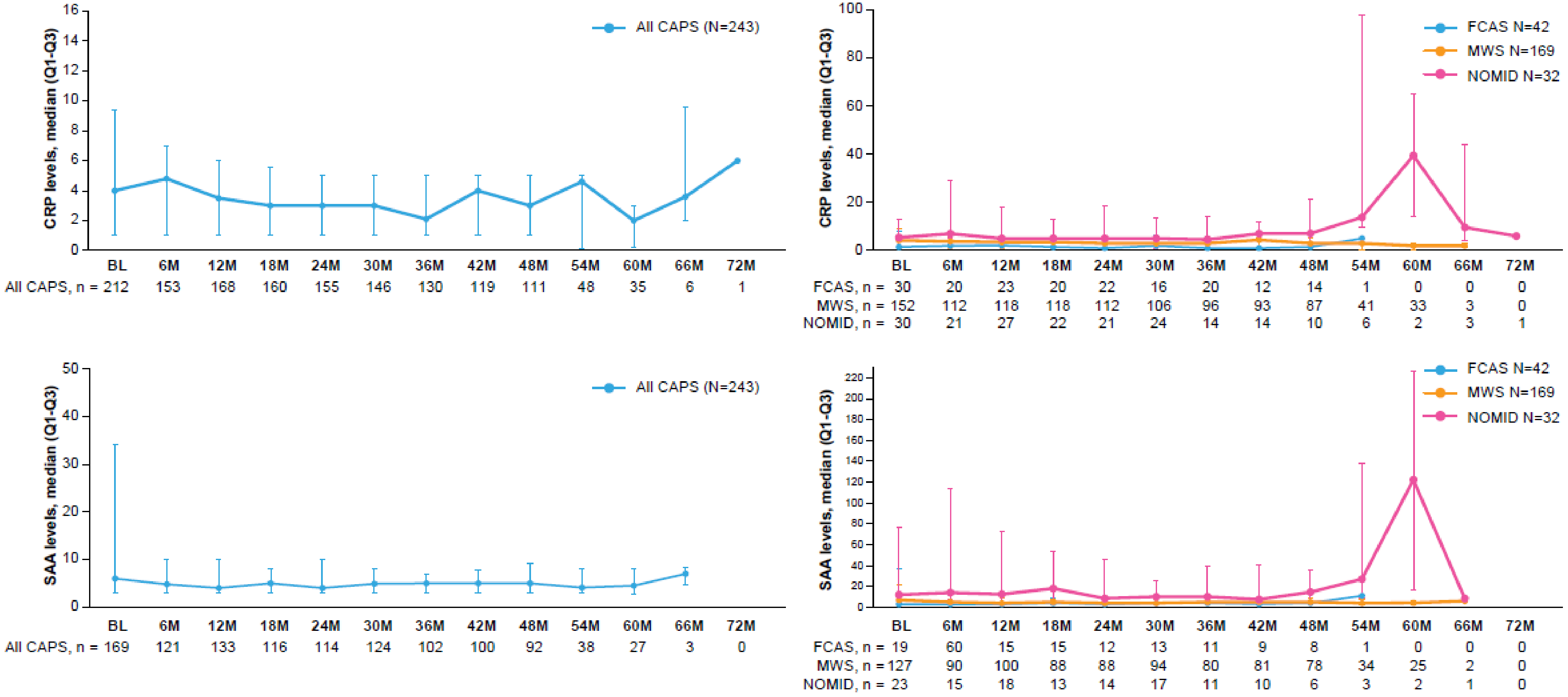


54

Panel A shows the PGA over time in FCAS, MWS or NOMID patients

Panel B shows PGA over time in All CAPS patients with or without exposure to anti–IL-1 treatment (including canakinumab) prior to enrolment

Panel C shows the CRP and SAA levels over time in ALL CAPS and 'Other' patients group

Percentages are based on the number of available patients at the respective time point

*Patients not exposed to canakinumab as part of a clinical trial and not exposed to any other IL-1 treatment might have started to use marketed canakinumab several weeks before the beginning of the registry.

N, number of evaluable patients; n, number of patients available for assessment at each time point; vertical bars represent Q1 and Q3

BL, baseline; CAPS, cryopyrin-associated periodic syndromes; CRP, C-reactive protein; FCAS, familial cold autoinflammatory syndrome; IL, interleukin; M, months; MWS, Muckle–Wells syndrome; N, number of evaluable patients; n, number of patients available for assessment at each time point; NOMID, neonatal-onset multisystem inflammatory disease; PGA, Physician's Global Assessment; Q, quartile; SAA, serum amyloid A.



Walker UA, *et al. RMD Open* 2021; 7:e001663. doi: 10.1136/rmdopen-2021-001663
